# An integrative phylogenomic approach to elucidate the evolutionary history and divergence times of Neuropterida (Insecta: Holometabola)

**DOI:** 10.1186/s12862-020-01631-6

**Published:** 2020-06-03

**Authors:** Alexandros Vasilikopoulos, Bernhard Misof, Karen Meusemann, Doria Lieberz, Tomáš Flouri, Rolf G. Beutel, Oliver Niehuis, Torsten Wappler, Jes Rust, Ralph S. Peters, Alexander Donath, Lars Podsiadlowski, Christoph Mayer, Daniela Bartel, Alexander Böhm, Shanlin Liu, Paschalia Kapli, Carola Greve, James E. Jepson, Xingyue Liu, Xin Zhou, Horst Aspöck, Ulrike Aspöck

**Affiliations:** 1 Centre for Molecular Biodiversity Research, Zoological Research Museum Alexander Koenig, 53113 Bonn, Germany; 2 Department of Evolutionary Biology and Ecology, Institute of Biology I (Zoology), Albert-Ludwigs-Universität Freiburg, 79104 Freiburg, Germany; 3 Australian National Insect Collection, National Research Collections Australia, Commonwealth Scientific and Industrial Research Organisation (CSIRO), Canberra, ACT 2601 Australia; 4 Department of Genetics, Evolution and Environment, University College London, London, WC1E 6BT UK; 5 Institut für Zoologie und Evolutionsforschung, Friedrich-Schiller-Universität Jena, 07743 Jena, Germany; 6 Natural History Department, Hessisches Landesmuseum Darmstadt, 64283 Darmstadt, Germany; 7 Steinmann-Institut für Geologie, Mineralogie und Paläontologie, Rheinische Friedrich-Wilhelms-Universität Bonn, 53115 Bonn, Germany; 8 Centre for Taxonomy and Evolutionary Research, Arthropoda Department, Zoological Research Museum Alexander Koenig, 53113 Bonn, Germany; 9 Department of Evolutionary Biology, University of Vienna, 1090 Vienna, Austria; 10 Department of Entomology, China Agricultural University, 100193 Beijing, People’s Republic of China; 11 LOEWE Centre for Translational Biodiversity Genomics (LOEWE-TBG), 60325 Frankfurt, Germany; 12 School of Biological, Earth and Environmental Sciences, University College Cork, Distillery Fields, North Mall, T23 N73K Cork, Ireland; 13 Institute of Specific Prophylaxis and Tropical Medicine, Medical Parasitology, Medical University of Vienna (MUW), 1090 Vienna, Austria; 14 Zoological Department II, Natural History Museum of Vienna, 1010 Vienna, Austria

**Keywords:** Megaloptera, Neuroptera, Raphidioptera, Endopterygota, Transcriptomics, RNA-seq, Multi-species coalescent, Supermatrices, Four-cluster likelihood mapping

## Abstract

**Background:**

The latest advancements in DNA sequencing technologies have facilitated the resolution of the phylogeny of insects, yet parts of the tree of Holometabola remain unresolved. The phylogeny of Neuropterida has been extensively studied, but no strong consensus exists concerning the phylogenetic relationships within the order Neuroptera. Here, we assembled a novel transcriptomic dataset to address previously unresolved issues in the phylogeny of Neuropterida and to infer divergence times within the group. We tested the robustness of our phylogenetic estimates by comparing summary coalescent and concatenation-based phylogenetic approaches and by employing different quartet-based measures of phylogenomic incongruence, combined with data permutations.

**Results:**

Our results suggest that the order Raphidioptera is sister to Neuroptera + Megaloptera. Coniopterygidae is inferred as sister to all remaining neuropteran families suggesting that larval cryptonephry could be a ground plan feature of Neuroptera. A clade that includes Nevrorthidae, Osmylidae, and Sisyridae (i.e. Osmyloidea) is inferred as sister to all other Neuroptera except Coniopterygidae, and Dilaridae is placed as sister to all remaining neuropteran families. Ithonidae is inferred as the sister group of monophyletic Myrmeleontiformia. The phylogenetic affinities of Chrysopidae and Hemerobiidae were dependent on the data type analyzed, and quartet-based analyses showed only weak support for the placement of Hemerobiidae as sister to Ithonidae + Myrmeleontiformia. Our molecular dating analyses suggest that most families of Neuropterida started to diversify in the Jurassic and our ancestral character state reconstructions suggest a primarily terrestrial environment of the larvae of Neuropterida and Neuroptera.

**Conclusion:**

Our extensive phylogenomic analyses consolidate several key aspects in the backbone phylogeny of Neuropterida, such as the basal placement of Coniopterygidae within Neuroptera and the monophyly of Osmyloidea. Furthermore, they provide new insights into the timing of diversification of Neuropterida. Despite the vast amount of analyzed molecular data, we found that certain nodes in the tree of Neuroptera are not robustly resolved. Therefore, we emphasize the importance of integrating the results of morphological analyses with those of sequence-based phylogenomics. We also suggest that comparative analyses of genomic meta-characters should be incorporated into future phylogenomic studies of Neuropterida.

## Background

The insect superorder Neuropterida contains more than 6500 described and extant species that are classified into three holometabolous insect orders: Megaloptera (alderflies, dobsonflies and fishflies), Neuroptera (lacewings, antlions and relatives) and Raphidioptera (snakeflies). Among these three, Neuroptera is by far the most species-rich order with 5917 species, in comparison to the much less diverse Megaloptera and Raphidioptera (386 and 253 species respectively) [1]. Within Holometabola, Neuropterida is considered the sister group of Coleopterida, and both together form the clade Neuropteroidea (or Neuropteriformia) [2–4]. Overall, the monophyly of Neuropterida is well established but morphological evidence in support of this monophyly is only based on a small number of inconspicuous characters (summarized by Aspöck, 2002 [5] and by Aspöck et al. 1980 [6]). The phylogenetic relationships of neuropterid insects have received considerable attention based on the analyses of different types of data such as the anatomy of adults [7–12], or the anatomy of larvae [9, 13–15]. Other studies have combined morphological and molecular evidence in a phylogenetic framework [16, 17], and recently several studies have analyzed genome-scale molecular datasets [18–23]. These phylogenomic studies have included analyses of different types of data such as hybrid enrichment data [20, 24], mitochondrial genome sequences [18, 19, 21, 22], and transcriptomic data [23]. Analyses of these types of data did not reach a full consensus on the phylogenetic relationships of Neuropterida, specifically concerning the backbone tree of Neuroptera. Here, we present the largest dataset of phylogenetically informative molecular characters compiled to date, across a large number of neuropterid and outgroup species, in an attempt to resolve the existing phylogenetic uncertainties in the phylogeny of Neuropterida and infer the temporal pattern of diversification within the group. A further important goal of this study is to identify sources of phylogenetic signal in the data and assess the effects of confounding factors on the phylogenetic reconstructions, in order to identify methodological problems behind open questions or conflicting phylogenetic results.

Recent phylogenetic investigations of Neuropterida have converged on the hypothesis that the order Raphidioptera is sister to a clade comprising Megaloptera and Neuroptera [11, 17, 19–21, 25–27]. Raphidioptera is a relict group of holometabolous insects with most of its species geographically distributed over small areas in the northern hemisphere (except eastern North America) [26, 28]. Owing to their distinctly higher species diversity in the Mesozoic, and their very limited morphological divergence since then, some authors refer to them as “living fossils” [20, 28–31]. The order is divided into two extant families: Raphidiidae (209 described extant species) and Inocelliidae (44 described extant species) [1]. The monophyly of Raphidioptera and of each raphidiopteran family is well established. However, previous phylogenomic analyses of Neuropterida have suffered from taxon-sampling limitations within the order [20, 21, 23]. Therefore, a comprehensive phylogenetic analysis of snakeflies based on the analysis of genomic sequence data has yet to be performed. The order Megaloptera comprises two extant families: Corydalidae (Corydalinae: dobsonflies and Chauliodinae: fishflies with 303 described extant species in total) and Sialidae (alderflies: 83 described extant species) [1]. This order includes the oldest known holometabolous insects with an aquatic lifestyle of the larvae [32]. The monophyly of Megaloptera has been questioned before [16, 17, 33, 34], as has been the monophyly of the family Corydalidae [35]. Nevertheless, recent morphological and molecular evidence suggests that Corydalidae and Sialidae are monophyletic sister taxa within the monophyletic Megaloptera [11, 20, 21, 36].

The order Neuroptera comprises 16 extant families. In comparison to the adults, the larvae of Neuroptera have evolved a very broad spectrum of morphological adaptations to very different habitats and lifestyles [17, 20]. Only two neuropteran families contain species with strictly aquatic larvae (i.e. Nevrorthidae, Sisyridae) [20, 37]. The larvae of Sisyridae (spongillaflies) use freshwater bryozoans and sponges as hosts, whereas the larvae of Nevrorthidae (mermaids) are generalist benthic predators [17, 37]. Other remarkable adaptations of the larvae within Neuroptera include predators of termites (some Berothidae) [38–40], parasitoids of bees and wasps (Mantispidae: some Symphrasinae) [41], predators of spider eggs (Mantispidae: Mantispinae) [42, 43], fossorial pit-trap builders (some Myrmeleontidae) [14, 17, 20, 44, 45], and possibly also phytophagous root suckers (Ithonidae, *Oliarces*) [46]. The monophyly of Neuroptera has never been questioned and is strongly supported by the unique and complex sucking tubes of the larvae [20, 29]. However, there is currently a lack of consensus on the phylogeny of neuropteran families mainly because analyses of different types of phylogenomic data have suggested conflicting topologies. In addition, the morphological characters of the adults are affected by homoplasy [7, 15] and although larval morphology yields important information, the phylogenetic signal from analyzing larval characters appears to be partly eroded [17, 20, 21], probably due to far-reaching specialization, especially in the case of the miniaturized Coniopterygidae (dustywings).

Concerning the phylogeny of neuropteran families, conflicting phylogenetic results have emerged both among different molecular studies [20, 21] as well as among different datasets or methods applied within the same study [20]. One example of conflicting hypotheses concerns the monophyly, or non-monophyly, of the suborder Myrmeleontiformia [20, 21]. Myrmeleontiformia contains the five families Ascalaphidae (owlflies), Myrmeleontidae (antlions), Nemopteridae (thread-winged lacewings), Nymphidae (split-footed lacewings) and Psychopsidae (silky lacewings). The family Psychopsidae is most likely the sister group to all remaining Myrmeleontiformia, as suggested by analyses of morphological characters [12, 15, 44, 47, 48]. It should, however, be noted that similar complex male genital sclerites of Psychopsidae and Nemopteridae have been interpreted as synapomorphies indicating a possible sister group relationship of these two families [11]. Recently, target DNA enrichment-based phylogenomic analyses suggested a clade of Ithonidae (moth lacewings) + Nymphidae, implying paraphyletic Myrmeleontiformia [20, 24]. In contrast, phylogenetic analyses of mitochondrial genomes did not corroborate this result but suggested monophyletic Myrmeleontiformia [21, 22]. Other conflicting hypotheses among previous phylogenomic studies include the disruption, or not, of a clade comprising Chrysopidae (green lacewings) and Hemerobiidae (brown lacewings) and the exact affinities of these two families to a clade of Ithonidae + Myrmeleontiformia [20–22]. A clade comprising Mantispidae (mantid lacewings), Berothidae (beaded lacewings), and Rhachiberothidae (thorny lacewings), collectively referred to as Mantispoidea [9, 20], was recovered in all previous phylogenomic studies, but the exact placement of this clade within Neuroptera remains elusive. Lastly, the inter-relationships of Osmylidae (lance lacewings), Nevrorthidae, and Sisyridae also remain unresolved. All previous phylogenomic studies suggested that these three families branch off close to the base of the neuropteran tree, but reconstructed different topologies among these groups [20–22].

Despite the above-outlined discrepancies among phylogenomic studies, some results seem to be robust across phylogenomic studies, but they are in conflict with the results of morphological studies. Such conflicts include the phylogenetic placement of Coniopterygidae as sister to the remaining families of Neuroptera, as suggested by previous analyses of genomic sequence data, but also by analyses of a small number of molecular markers [17], or by total evidence analyses [16]. Most cladistic analyses of morphological characters instead suggest that Nevrorthidae is the sister group to all other neuropteran families [9, 11, 12, 15, 47]. The family Sisyridae has also been proposed as sister to all other Neuroptera based on the analysis of morphological characters [10]. A consensus on the basal splitting patterns within Neuroptera is essential for inferring the ancestral lifestyle of the neuropteran larvae, and also for tracing morphological character evolution within the order [20]. Most importantly, the paraphyly of Myrmeleontiformia as suggested by target DNA enrichment-based phylogenomic studies, was a surprising result especially given the long-lasting [49] and strong support of morphological studies in favor of monophyletic Myrmeleontiformia. Hence, a reevaluation of the previously proposed paraphyly of Myrmeleontiformia based on other kinds of data or methods is needed [48].

Previous molecular studies of the phylogeny of Neuropterida have mostly relied on conventional measures of branch support, such as the non-parametric bootstrap [50] and the Bayesian posterior probabilities [51]. However, the usage of these measures alone has often proven insufficient for the purpose of estimating the robustness of the inferred molecular phylogenies [52–57], especially when the size of the dataset increases [58–62], or when overly simplified evolutionary models are used [63, 64]. A plethora of quartet-based approaches for estimating phylogenomic incongruence and node certainty in molecular phylogenies has been proposed lately [4, 52, 65–68]. These approaches rely on the calculation of phylogenetic signal from quartets of taxa and they can be used to identify conflicting signals and potentially inflated support for certain phylogenetic clades, but have not yet been applied to the phylogeny of Neuropterida. Given the putatively misleading nature of the existing branch support measures in a maximum likelihood or Bayesian phylogenetic framework, combined with the incongruent results of previous phylogenomic studies, a thorough evaluation of the conflicts in the phylogenetic tree of Neuropterida is currently needed.

The purpose of this study is to provide: 1) a phylogenomic framework and updated divergence time estimates of Neuropterida, 2) an evaluation of conflicting phylogenetic signals in the backbone phylogeny of the group, and 3) a discussion of the implications for morphological character evolution within Neuropterida based on the results of the present contribution and those of other studies. In an effort to resolve the existing incongruencies we assembled a novel transcriptomic dataset of Neuropterida and of suitable outgroup species, and assessed the robustness of our phylogenetic estimates with concatenation-based quartet approaches combined with data permutations and with gene tree-based quartet approaches. We additionally estimated divergence times of the major lineages of Neuropterida by using an approach that enables monitoring the effect of data selection on the Bayesian posterior divergence times of Neuropterida.

## Results

### Orthology assignment, alignment refinement, protein domain identification and supermatrix evaluation

On average, 3292 sequences per transcriptome or official gene set (OGS) passed the reciprocal best-hit criterion during the orthology assignment step (max. = 3909, min. = 1935). We excluded a total number of 21 transcriptomes and OGSs from our dataset because we found too few target genes (orthologs) within them (Additional file 1: Table S1). The majority of the excluded transcriptomes and OGSs refer to outgroup taxa (17 outgroup and four ingroup species). Alignment masking resulted in removal of a total number of 1,307,572 alignment sites at the amino-acid sequence level (~ 45% of alignment sites). Concatenation of the masked amino-acid sequence alignments resulted in a supermatrix composed of 6869 domain-based partitions spanning more than 1.5 million amino-acid alignment sites (supermatrix A, Table 1). Supermatrices E and F did not significantly differ in their overall completeness, data coverage in terms of presence/absence of partitions (i.e. saturation, Table 1), information content and deviation from stationary, (time-) reversible and homogeneous (SRH) conditions (Table 1). We selected supermatrix E for downstream analyses due to its larger size in terms of total alignment length and number of partitions, (see Additional file 2). The optimization of the partitioning scheme of supermatrix E with the software PartitionFinder resulted in a total number of 1825 meta-partitions.

**Table 1:** Descriptive statistics for each of the analyzed amino-acid supermatrices that were partitioned according to protein-domain clans, protein families and to single protein domains. Information content calculated with the software MARE is a relative measure of phylogenetic informativeness and data coverage. Completeness scores calculated with AliStat indicate the proportion of non-ambiguous characters

Amino-acid supermatrix	No. of alignment sites	No. of domain-based partitions or meta-partitions	No. of species	Information content (MARE)	Saturation (MARE)	Completeness score (C_**a**_) (AliStat)	Median pairwise p-value for the Bowker’s test (SymTest)
**A**	1,550,004	6869 partitions	121	0.432	0.804	0.628	2.22e-141
**B**	1,087,525	4261 partitions	119	0.636	0.909	0.659	8.22e-092
**C**	1,506,256	5353 partitions	121	0.554	0.820	0.628	4.46e-137
**D**	1,506,256	5353 partitions	119	0.557	0.826	0.635	8.68e-137
**E**	931,450	3635 partitions	119	0.667	0.923	0.657	8.13e-068
**F**	920,182	3603 partitions	119	0.669	0.923	0.657	1.40e-066
**E (RCFV-corrected)**	383,656	314 (meta-partitions)	119	0.662	0.997	0.713	9.33e-018
**E (Decisive)**	228,933	209 (meta-partitions)	119	0.619	1.000	0.796	3.29e-013

### Phylogeny of Neuropterida: concatenation-based and summary coalescent phylogenetic analyses

**Fig. 1:**
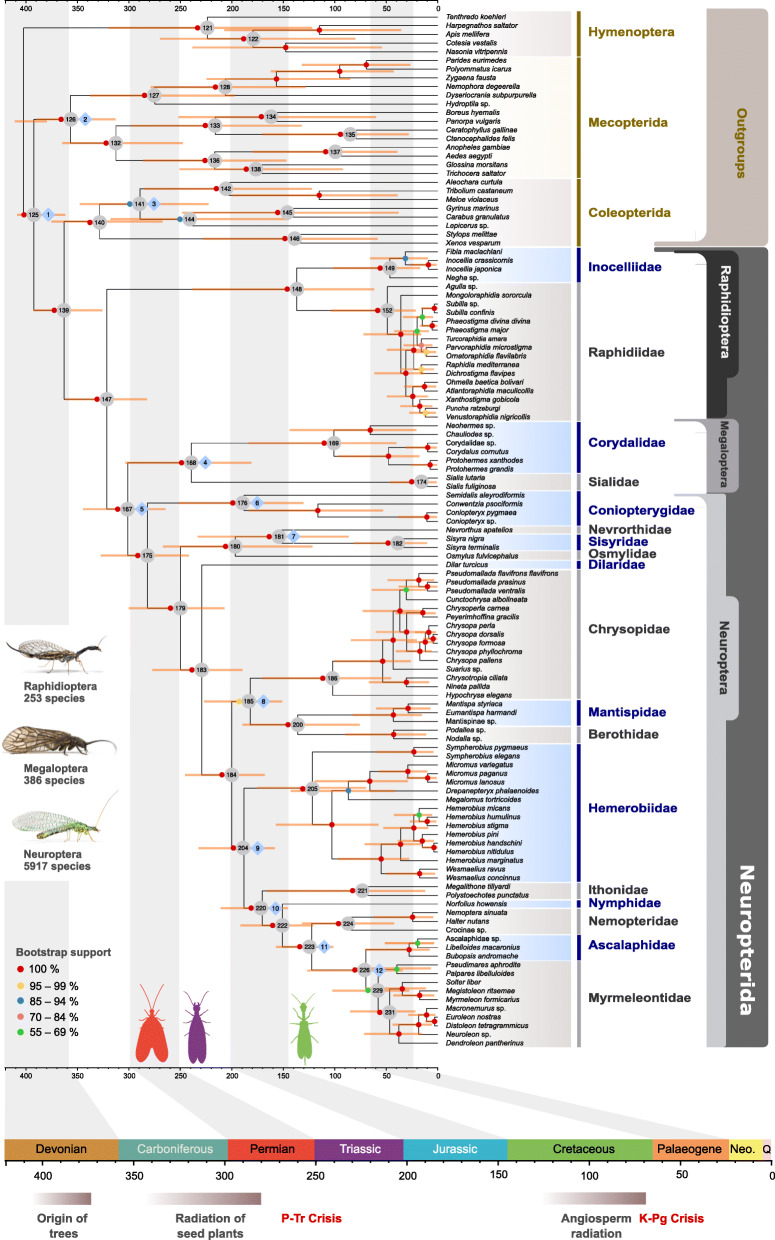
Phylogenetic relationships of Neuropterida based on the analyses of the concatenated amino-acid sequence data of supermatrix E. Colored circles depict phylogenetic branch support values based on 100 non-parametric bootstrap replicates. Bars on the individual nodes show the 95% confidence intervals (equal-tail CI) of the posterior divergence time estimates. Blue squares indicate the time-calibrated nodes. Divergence time estimates were calculated from a single summarized MCMC chain (first independent analysis, run 1) that included all parameter values from each individual meta-partition analysis when including all fossil calibrations. Insect photos from top to bottom: *Dichrostigma flavipes*, *Sialis lutaria*, *Chrysopa perla* (all photos by O. Niehuis)

Phylogenetic analyses of the domain-based partitioned amino-acid sequence data yielded congruent topologies (with respect to the phylogenetic relationships of major lineages) with those obtained when analyzing the second codon positions of the nucleotide sequence data (Fig. 1, Additional file 3: Figures S1–S5). In addition, the phylogenetic trees yielded by the analyses of the reduced amino-acid supermatrices (decisive and RCFV-corrected versions of supermatrix E, Table 1) are topologically congruent with trees that resulted from the analyses of the above-mentioned datasets, concerning the phylogenetic relationships within Neuropterida (Additional file 3: Figures S6–S9). Analyses with the site-heterogeneous mixture models also delivered topologies congruent to the analyses of the above-mentioned datasets (Additional file 3: Figures S10–S14). All these analyses support Coleopterida (Coleoptera + Strepsiptera) as sister to Neuropterida, the monophyly of all neuropterid orders and families, and the sister group relationship between Raphidioptera and Megaloptera + Neuroptera (Fig. 1, Additional file 3: Figures S1–S14).

**Fig. 2:**
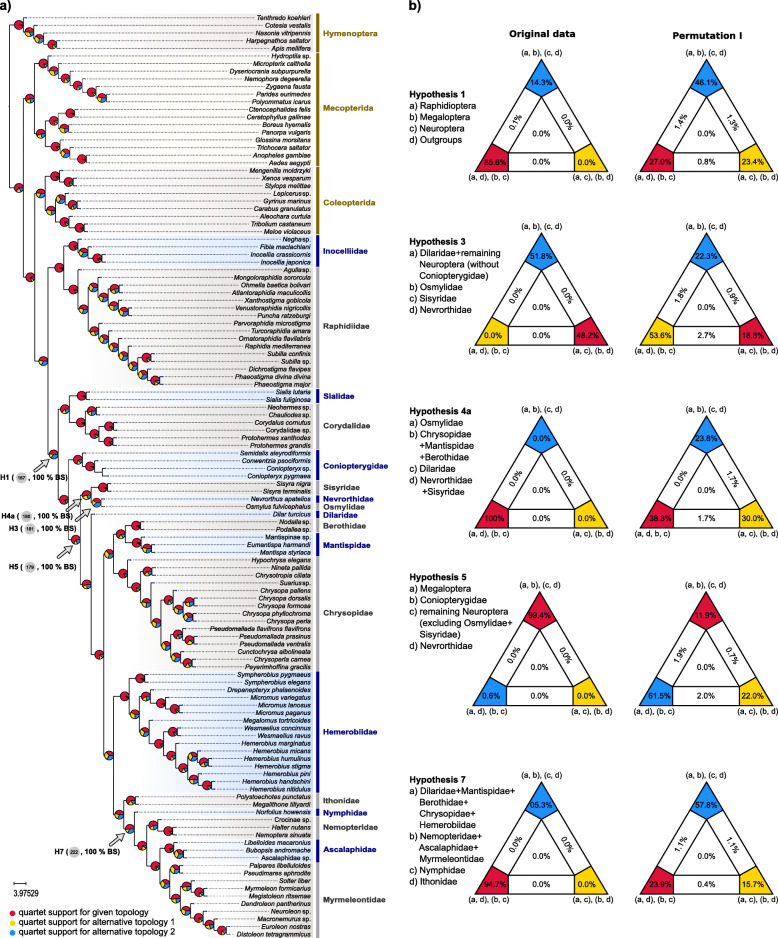
Gene tree-based and concatenation-based quartet analyses of the phylogenetic relationships of Neuropterida. **a** Phylogenetic relationships of Neuropterida, as they resulted from the summary coalescent phylogenetic analysis with ASTRAL, when analyzing the full set of gene trees (3983 gene trees inferred at the amino-acid sequence level). Pie charts on branches show ASTRAL quartet support (quartet-based frequencies of alternative quadripartition topologies around a given internode). Arrows indicate the numbers of the corresponding tree nodes in Fig. 1, and the corresponding hypotheses in the FcLM analyses. **b** Results of FcLM analyses for a selection of phylogenetic hypotheses applied at the amino-acid sequence level (supermatrix E). The first column shows the results of FcLM when the original data of supermatrix E were analyzed. The second column shows the results of FcLM after phylogenetic signal had been eliminated from supermatrix E (i.e. permutation no. I, see Additional file 2)

The inferred relationships within Raphidioptera suggest the monophyly of the family Raphidiidae, and the placement of the Nearctic genus *Agulla* as sister to a clade comprising all the Palearctic Raphidiidae. These relationships received maximum bootstrap and maximum bootstrap by transfer (TBE) support (Fig. 1, Additional file 3: Figure S2). Within the Palearctic Raphidiidae the genus *Mongoloraphidia* was inferred as the sister taxon to all remaining Raphidiidae. Within Neuroptera, a sister group relationship between Coniopterygidae and all remaining neuropteran families received maximum bootstrap and maximum TBE support (Fig. 1, Additional file 3: Figure S2). A clade comprising Osmylidae, Sisyridae, and Nevrorthidae (i.e. Osmyloidea [20]) was inferred as sister to all neuropteran families except Coniopterygidae. Dilaridae was placed as the sister group to all other Neuroptera except Coniopterygidae and Osmyloidea. A clade comprising Mantispidae and Berothidae (i.e. Mantispoidea excluding Rhachiberothidae for which transcriptomic data were not available) received high statistical branch support in all analyses of the above-mentioned analyzed datasets (Fig. 1, Additional file 3: Figures S1–S5). A sister group relationship between Ithonidae and Myrmeleontiformia (excluding Psychopsidae for which transcriptomic data were not available) was inferred with maximum bootstrap and maximum TBE support. Furthermore, analyses of concatenated domain-partitioned amino-acid data and those of second codon positions suggest Chrysopidae as sister to Mantispidae + Berothidae, and Hemerobiidae as the sister group of Ithonidae + Myrmeleontiformia (Fig. 1, Additional file 3: Figures S1–S5). Within Myrmeleontiformia, Nemopteridae is placed as sister to a clade of Ascalaphidae + Myrmeleontidae. Even though non-parametric bootstrap and TBE support for the monophyly of Myrmeleontidae + Ascalaphidae is high, non-parametric bootstrap support for the monophyly of Myrmeleontidae is very low (Fig. 1). These results were congruent with the results of the summary coalescent analyses of gene partitions at the amino-acid sequence level, except for the sister group relationship of *Mongoloraphidia* to the remaining Palearctic Raphidiidae (Fig. 2a, Additional file 3: Figures S15–S17, see also Additional file 2). Within Neuroptera, the results of the phylogenetic analyses of domain-based partitioned amino-acid sequence data are also congruent with the concatenation-based analyses of genes at the amino-acid sequence level, except for the disruption of the clade Mantispoidea + Chrysopidae in the concatenated analyses of unmasked gene alignments with increased species coverage (Additional file 3: Figures S18–S21).

The summary coalescent analyses and the concatenation-based analyses of gene partitions when analyzing codon-based nucleotide sequence data (with all codon positions included) suggest different topologies concerning the inter-familiar phylogenetic relationships of Neuroptera (Additional file 3: Figures S22–S29, see also Additional file 2). Specifically, analyses of the codon-based nucleotide sequence data with both methods yielded paraphyletic Myrmeleontiformia and further suggest a sister group relationship of Chrysopidae with a clade of Ithonidae + paraphyletic Myrmeleontiformia (Additional file 3: Figures S22–S29). Additional topological differences concern the inferred relationships within Osmyloidea depending on the method and the data type analyzed (e.g. Figures 1 and 2 and Additional file 3: Figures S1–S29, see also Additional file 2). Overall, the topological differences inferred from the different analyses mainly concern the inter-relationships of the four monophyletic groups: Chrysopidae, Hemerobiidae, Mantispoidea, Ithonidae + Myrmeleontiformia. The different hypotheses concerning the relationships of these four groups (e.g. Hemerobiidae vs. Chrysopidae as sister to Ithonidae + Myrmeleontiformia), are characteristic of the different types of data that were analyzed (i.e. amino-acid vs. codon-based nucleotide sequence data with all codon positions included, see Additional file 2). The family Hemerobiidae was inferred as sister to Ithonidae + monophyletic Myrmeleontiformia when analyzing amino-acid sequences or second-codon positions of nucleotide sequences, irrespective of the applied phylogenetic method (i.e. concatenation vs. summary coalescent phylogenetic analysis, Figs. 1 and 2, Additional file 3: Figures S1–S14, S15, S18), or partitioning strategy (i.e. domain-based partitioning vs. gene-based partitioning, Additional file 3: Figures S1–2, S10–14, S18–S21).

### Tests for the presence of confounding signal via four-cluster likelihood mapping and data permutations

The four-cluster likelihood mapping (FcLM) approach delivered strong statistical support for most inferred phylogenetic relationships (Additional file 1: Table S2). For example, a clade Megaloptera + Neuroptera is strongly supported by the FcLM analyses with no detectable confounding signal (Fig. 2b, Hypothesis 1: 85.60% of quartets). Support for Coniopterygidae instead of Nevrorthidae as the sister group to the remaining Neuroptera also received strong FcLM support without detectable confounding signal (Fig. 2b, Hypothesis 5: 99.40% of quartets). The monophyly of Osmyloidea is also strongly supported without detectable confounding signal (99.70% of quartets, Hypothesis 8, Additional file 1: Table S2, see also Hypothesis 4a). A potential sister group relationship of Osmylidae and Chrysopidae, as suggested by some previous morphological studies, is not supported by the FcLM branch support tests (Hypotheses 4a and 4b, Fig. 2b and Additional file 1: Table S2). The monophyly of Myrmeleontiformia (Nymphidae, Nemopteridae, Ascalaphidae, Myrmeleontidae) is strongly supported by our FcLM tests without detectable confounding signal (Fig. 2b, Hypothesis 7: 94.70% of quartets).

Nevertheless, the results of FcLM analyses showed conflicting signal for some splits in the backbone tree of Neuroptera (Fig. 2b, Additional file 1: Table S2). For example, the FcLM analyses do not unequivocally support the sister group relationship of Sisyridae and Nevrorthidae (i.e. 51.80% of quartets support Nevrorthidae + Sisyridae, Fig. 2b, Additional file 1: Table S2, Hypotheses 2 and 3). Moreover, FcLM analyses do not unequivocally support a clade Mantispoidea + Chrysopidae (46.10% of quartets, Hypothesis 9, Additional file 1: Table S2). The sister group relationship of Hemerobiidae to Ithonidae + Myrmeleontiformia received only moderate support in FcLM analyses (72.40% of quartets in Hypothesis 6a). FcLM analyses on the permuted matrices showed that there was no substantial contribution of confounding factors for this sister group relationship, although there exists some weak signal (43.30% of quartets) possibly originating from non-random distribution of missing data in support of the results of tree reconstructions (Hypothesis 6a, permutations I and II, Additional file 1: Table S2). When using a different definition of groups of taxa, the placement of Hemerobiidae as sister to Ithonidae + Myrmeleontiformia was supported by only 36.60% of the analyzed quartets (Hypothesis 6b, Additional file 1: Table S2).

### Divergence times of Neuropterida

**Fig. 3:**
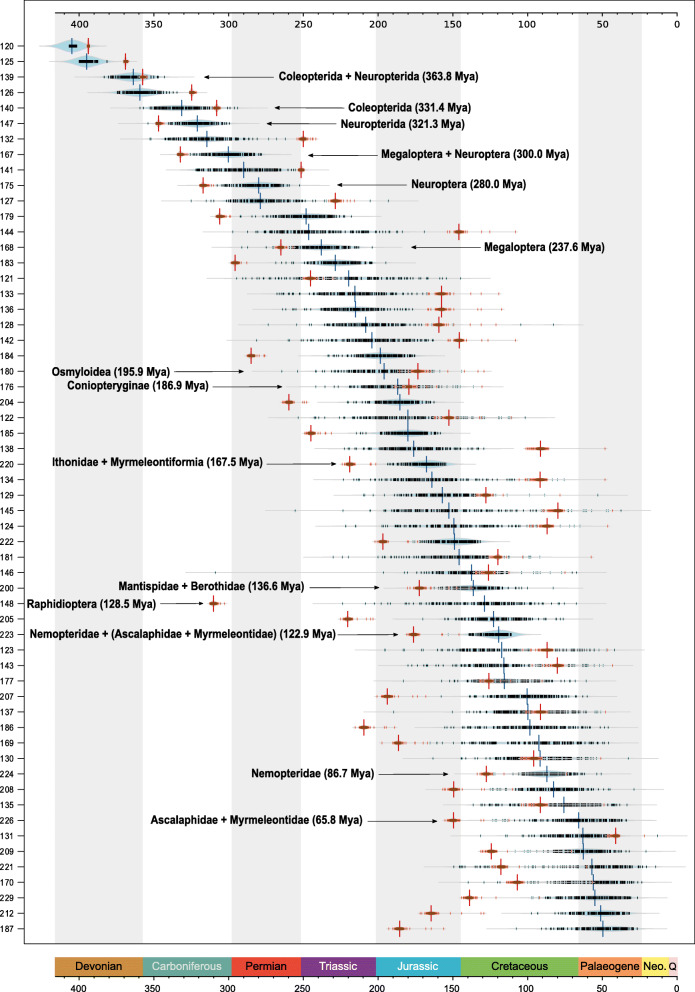
Distribution of the median posterior node ages among the different meta-partitions. Arrows indicate the corresponding crown groups of Neuropterida and outgroups. Numbers on x-axis correspond to the node number ids of the tree in Fig. 1. The distribution of the median posterior age estimates of the individual meta-partitions from the first independent dating analysis (α = 2, run 1) is shown in blue. The distribution of the median age estimates when running the analyses without data (i.e. marginal prior) is shown in red

Our molecular-dating analyses illustrate that most meta-partitions contained enough signal to overrule the prior assumptions (i.e. marginal prior distributions) on the divergence times of Neuropterida (Fig. 3), except for the ancient splits within the outgroup taxa. Given a fixed topology and node-age calibrations, the distribution of median posterior divergence times among meta-partitions when compared with the distribution of the median values of the marginal prior distributions, constitutes evidence for the dominant influence of signal in the datasets (Fig. 3). It does however also show extensive variation in signal among meta-partitions. This variation in signal is more prominent for certain nodes (e.g. crown Raphidioptera, Fig. 3), whereas the individual median posterior age estimates are less dispersed compared to the overall median for others (e.g. crown Ithonidae + Myrmeleontiformia).

The combined dating analysis of the meta-partitions from the first run in MCMCTree (Fig. 1, Additional file 1: Table S3) suggests that the phylogenetic split between Coleopterida and Neuropterida (i.e. Neuropteroidea) occurred in the end of the Devonian period (median = 364.3 Mya, CI = 392.9–325.9, Additional file 1: Tables S3, S4). Crown Neuropterida started to diversify in the middle of Carboniferous (median = 321.7 Mya, CI = 362.0–282.4 Mya). Although Raphidioptera was inferred as the earliest branching lineage within Neuropterida, the most recent common ancestor of crown Raphidioptera was estimated to have lived at the beginning of the Cretaceous period (median = 132.1, CI = 238.2–61.7 Mya). There is extensive variation in signal among meta-partitions for this particular split (Fig. 3) that is reflected in the very wide confidence intervals (95% equal-tail and 95% higher posterior density CI, Fig. 1, Additional file 1: Tables S3, S4). The split between the Nearctic *Agulla* and all remaining Raphidiidae in the dataset was estimated to have occurred in the middle of the Eocene (median = 44.1, CI = 103.6–21.1 Mya). The split of crown Megaloptera was estimated to have occurred at the beginning of the Triassic period (median = 238.9, CI = 303.4–180.8 Mya), while crown Neuroptera started to diversify much earlier at the beginning of the Permian (median = 280.8, CI = 327.4–241.7 Mya). The crown group of Osmyloidea started to diversify at the beginning of the Jurassic (median = 197.4, CI = 266.7–121.7 Mya). Many consecutive deep splits in the phylogeny of Neuroptera (e.g. crown Osmyloidea, crown Coniopteryginae, and the split between Hemerobiidae, Mantispoidea, Chrysopidae, and Myrmeleontiformia) were estimated to have occurred at the end of the Triassic or the beginning of the Jurassic (Figs. 1 and 3). Lastly, most crown groups of the different neuropterid families (e.g. the crown groups of Chrysopidae, Hemerobiidae, Nemopteridae, Ithonidae, and the common ancestor of Ascalaphidae + Myrmeleontidae) started to diversify during the Cretaceous (Fig. 1). Posterior node-age estimates and confidence intervals that resulted from the combined analysis of the second independent run (run 2) with MCMCTree are very similar (Additional file 1: Table S4), which suggests that the two independent chains (each composed of the combined parameter values of the individual meta-partitions) have converged to very similar posterior node-age estimates (Additional file 3: Figures S30, S31).

### Evolution of larval characters and lifestyles within Neuropterida

**Fig. 4:**
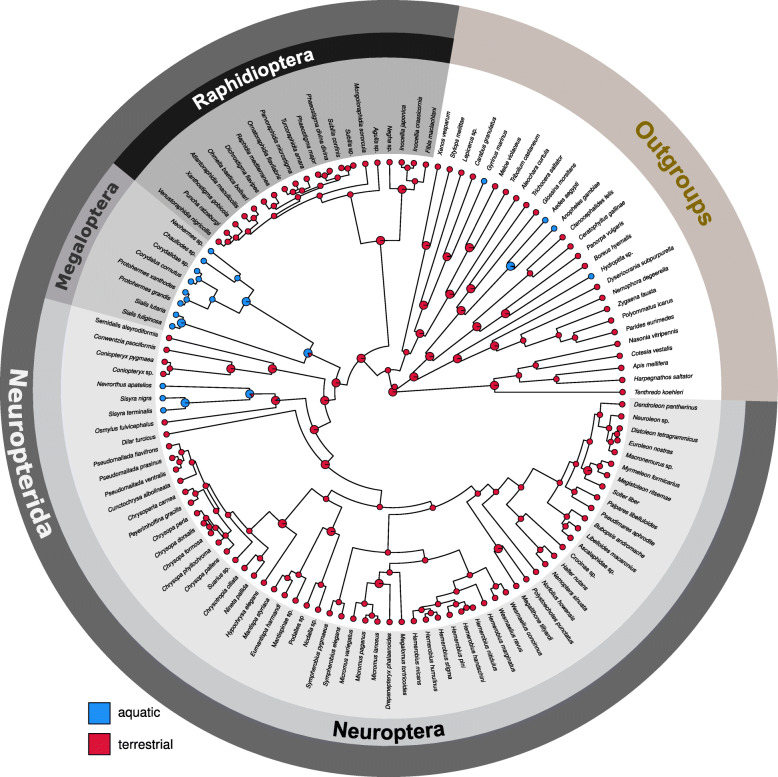
Summarized results of stochastic character mapping analyses (SCM) for the evolution of larval ecologies based on 10,000 sampled character histories. Stochastic character maps were generated under the ER model and by using the topology and branch lengths of the chronogram of Fig. 1. Colored circles at the tips show the coded state for each species. Pie charts on internal tree nodes show posterior probabilities of states at each node under the model used. Internal nodes with a posterior probability lower than 1.00 are depicted in larger size (note: for the SCM analyses we assumed that larval ecologies remain constant within the same family)

We traced the evolution of larval characters within Neuroptera based on the best topology (overall best maximum likelihood tree, ML tree, Fig. 1) that resulted from the analysis of domain-based partitioned amino-acid sequence data. The implications for the evolution of larval characters in Neuroptera under parsimony are outlined in Additional file 1: Table S5. Autapomorphies of Neuroptera, Myrmeleontiformia and Coniopterygidae (two terminals included in the studies by Beutel et al. 2010 [15] and Jandausch et al. 2018 [47]) are not affected by the phylogenetic pattern obtained in the present study. With the parsimony approach the reconstruction of ancestral states remained ambiguous with respect to the larval habitat of Neuroptera (terrestrial versus aquatic, Additional file 1: Table S5). In contrast, our Bayesian stochastic character mapping (SCM) analyses suggest a primarily terrestrial larval habitat in the last common ancestor of Neuroptera but also in the last common ancestor of the entire Neuropterida (Fig. 4). This result is recovered irrespective of the inferred relationships within Osmyloidea (Additional file 3: Figures S32–S34). Additionally, the parsimony-based analysis remained ambiguous with respect to the ancestral character state of the larval gula in Neuroptera. A large posterior sclerotized plate as it is present in Nevrorthidae (and also in Raphidioptera and Megaloptera) may be ancestral, with a small posterior rectangular sclerite preserved as vestige in Polystoechotinae, and a small anteromedian triangular sclerite as a de novo formation in Myrmeleontiformia. Following the principle of parsimony, the “maxillary head” as defined by Aspöck et al. (2001) [9] (i.e. the complete absence of a gula) could be a ground plan apomorphy of Neuroptera, and the secondary gain of a gula consequently an apomorphy of Nevrorthidae, Polystoechotinae and Myrmeleontiformia. The specialized terminal seta of the flagellum is interpreted as secondarily absent in Nevrorthidae on the one hand, and in Ithonidae and Myrmeleontiformia on the other, in the latter case as a potentially synapomorphic feature of these two groups. The poison channel and the intrinsic musculature of the maxillary stylets are secondarily absent in Sisyridae [47]. The trumpet-shaped empodium is likely an apomorphy of Neuroptera excluding Coniopterygidae and Osmyloidea, and the secondary loss of this feature is a synapomorphy of Ithonidae and Myrmeleontiformia [47]. The ground plan of Neuroptera with respect to the larval cryptonephry is ambivalent. This feature could represent an apomorphy of Neuroptera (Additional file 1: Table S5).

## Discussion

### Statistical robustness of phylogenomic results and potential pitfalls in phylogenetic reconstructions

Previously published phylogenomic analyses have suggested robustly resolved backbone trees of Neuropterida [17, 20–22] that were in part incongruent to inferred phylogenetic relationships based on analyses of morphological characters. The most recent molecular analyses at odds with morphological analyses were based on extensive genomic data [20, 21, 24] and therefore the incongruences between these molecular and morphological phylogenies cannot be easily dismissed. Since the accumulation and characterization of extensive genomic data is now the standard procedure in phylogenetics, as it is also true for the analyses of the phylogeny of Neuropterida, the evaluation of statistical robustness of the inferred phylogenies is becoming a complex yet essential task [69]. It is obvious that conventional analyses of statistical robustness, in most cases performed with the classical non-parametric bootstrap, might not scale well with the quantity of the data [59, 70–72]. This is because bootstrap support values provide an assessment of the sampling effects and repeatability of the analyses but cannot assess the accuracy of the inferred phylogenetic trees [71]. Alternative or complementary measures of phylogenomic incongruence are warranted to identify phylogenetic relationships with potentially inflated support [52, 53, 68, 73]. In order to identify potentially inflated branch support of the inferred relationships within Neuropterida, we have used a combination of gene tree-based and concatenation-based quartet methods and compared results with those of the classical non-parametric bootstrapping approach and with those of the newly described bootstrap by transfer support measure (TBE). We observed that a few seemingly well supported phylogenetic relationships assessed by bootstrapping are in fact inflated due to potentially confounding factors in the data. In most instances, concatenation-based and gene tree-based quartet methods deliver congruent pictures, that are in several cases in stark contrast to the classical resampling approaches. We conclude from these observations that at least parts of the backbone tree of Neuropterida should still not be considered robustly resolved. Below we discuss two examples from the backbone tree of Neuroptera that do not receive unequivocal support from our quartet analyses:

#### Phylogenetic relationships within Osmyloidea

We observed incongruent topologies between concatenation and the summary coalescent phylogenetic analyses concerning the splits within Osmyloidea. Summary coalescent phylogenetic analyses at the amino-acid sequence level suggest a clade of Sisyridae + (Osmylidae + Nevrorthidae), whereas all concatenated analyses of amino-acid sequence data suggested a clade of Osmylidae + (Nevrorthidae + Sisyridae). This incongruence between methods was only present when analyzing amino-acid sequence alignments. The analyses of the codon-based nucleotide sequence alignments (with all codon positions included) resulted in phylogenetic relationships congruent to the summary coalescent approach. Despite the high bootstrap and high TBE support from the concatenated analyses of amino-acid sequence data for a sister group relationship of Sisyridae and Nevrorthidae, our FcLM analyses do not unequivocally support the inferred phylogenetic relationships within Osmyloidea. Specifically, quartet support calculated with ASTRAL and FcLM analyses show almost equal proportions of quartets supporting each of the two above-mentioned prevalent phylogenetic hypotheses. Moreover, the FcLM analyses suggest substantial influence from taxon sampling and possibly from non-random distribution of missing data for this particular phylogenetic relationship. Putting the results of the concatenation-based, summary coalescent and FcLM analyses together, we conclude that the phylogenetic relationships of the three families in Osmyloidea should be considered for now unresolved.

#### Phylogenetic position of Hemerobiidae

Our analyses of amino-acid sequence data and those of second codon positions of the nucleotide sequence data, suggest Hemerobiidae as sister to Ithonidae + monophyletic Myrmeleontiformia, whereas analyses of the complete codon-based nucleotide sequence alignments suggest Chrysopidae as sister to Ithonidae + paraphyletic Myrmeleontiformia. These incongruencies again warrant a detailed examination of potentially confounding signals. The FcLM analyses do not unequivocally support Hemerobiidae as sister to Ithonidae + Myrmeleontiformia (72.40 and 36.60% of quartets), despite the maximum bootstrap and maximum TBE support for this relationship (100%). The FcLM analyses also show some weak putatively misleading signal in support of this relationship that possibly originates from non-random distribution of missing data. Since the FcLM and ASTRAL quartet analyses do not unequivocally support Hemerobiidae as sister to Ithonidae + Myrmeleontiformia, we consider this part of the neuropteran tree as statistically not robustly resolved.

### Different data types and not different tree-inference methods are responsible for some of the phylogenomic incongruences

Although many previous phylogenomic studies have focused on the biological causes of incongruence that results from analyzing the data with coalescent-based or concatenation-based phylogenetic methods [59, 74–76], little attention has been given to the effects of the different analyzed data types on phylogenetic inference [77]. Such data-type effects have been discussed before either in the context of analyzing different genomic regions, such as analyzing introns vs. analyzing coding sequences [78], or in the context of analyzing the same coding regions at different levels (i.e. nucleotides vs. amino acids) [77, 79, 80]. Here, we find that some of the inferred relationships within Neuroptera (i.e. the monophyly of Myrmeleontiformia and the position of Chrysopidae, Hemerobiidae and Mantispoidea) are characteristic of the data type that was analyzed (i.e. amino acids vs. codon-based nucleotide sequences with all codon positions included) irrespective of the tree-inference method. Given sufficient phylogenetic signal, the expectation is that the analyses of the same genomic regions at the nucleotide sequence level and the translational level should reflect the same evolutionary history. If the analyses of different data types result in discrepancies, this is most likely due to the failure of the applied substitution models to accommodate the evolutionary history in the analyzed data. Thus, the above-mentioned data-type effects probably stem from violations of the model assumptions by the analyzed data. Additionally, the observation that these data-type effects are quite robust across different tree-inference methods further suggests that both concatenation and summary coalescent methods are sensitive to these violations of model assumptions. An important open question is why some branches in the tree of Neuroptera may be more prone to data-type effects than others. Ancient rapid radiations have been proposed as candidates for such data-driven effects in phylogenetic reconstructions [78]⁠.

### Implications of our phylogenetic reconstructions concerning the evolution of Neuropterida

#### Inter-ordinal phylogenetic affinities of Neuroptera, Megaloptera, and Raphidioptera

Within holometabolous insects, Neuropterida is inferred as the sister group to Coleopterida, a phylogenetic hypothesis that is in accordance with the latest views on the phylogeny of Holometabola [2, 4]. The monophyly of Neuropteroidea (Coleopterida + Neuropterida) is supported by the presence of a prognathous or slightly inclined head in the adults of this group [2]. We estimated the most recent common ancestor of Neuropteroidea to have lived in the late Devonian (~ 363 Mya), an estimate that is earlier than what has been suggested [4, 81], and during a time interval that coincides with the appearance of the first tetrapod vertebrates and the formation of the first land forests.

In our study, the order Raphidioptera is placed as sister to Megaloptera + Neuroptera, in agreement with the results of most previous molecular studies [2, 4, 18–21, 82]. The notion that Megaloptera is the sister group to Neuroptera was first introduced by Boudreaux (1979) [83], on the premise of common wing venation characters. This idea was revived later with the argument that aquatic larvae represent a synapomorphic feature for Neuroptera and Megaloptera, with secondary terrestrialization in Neuroptera [84]. Our phylogenetic results and FcLM analyses are in agreement with the results of those morphological studies and with recent phylogenomic analyses of mitochondrial genomes or target DNA enrichment data concerning the inter-ordinal relationships of Neuropterida [18, 20, 21]. Hence, the traditional hypothesis that Neuroptera is the sister group to Megaloptera + Raphidioptera [33, 85–89], that was suggested by a few studies based on the analyses of a few genes [3, 90–92], is highly unlikely. We inferred the first split among the crown Neuropterida to have occurred in the middle of the Carboniferous (~ 321 Mya). This node-age estimate is slightly older than the age inferred in previously published phylogenomic studies, that proposed a common origin of the extant Neuropterida in the late Carboniferous or the early Permian [20, 21].

#### Evolutionary history of Raphidioptera

Within Raphidioptera, both Raphidiidae and Inocelliidae are recovered as monophyletic in all of our analyses and with high statistical support. We estimated the common ancestor of extant Raphidioptera to have lived during the early Cretaceous (~ 136 Mya), although it is evident from the fossil record that stem lineages of Raphidioptera were distinctly diverse much earlier in the Mesozoic [29]. Our results suggest the placement of the Nearctic genus *Agulla* as sister to the Palearctic Raphidiidae. Although the Nearctic genus *Alena* is not included in our analyses, the above-mentioned relationship suggests the monophyly of the Palearctic Raphidiidae and corroborates previous molecular phylogenetic analyses of Raphidiidae [26]. Furthermore, the results of the analyses of domain-based partitioned data are in agreement with previous molecular phylogenetic analyses of the Raphidiidae, that suggested the division of the Palearctic Raphidiidae into an Eastern Palearctic (*Mongoloraphidia* clade) and a Western Palearctic (*Ohmella*, *Puncha* and *Phaeostigma* clades) radiation [26]. Biogeographical aspects of the phylogeny of extant Raphidioptera are discussed in more detail by Aspöck et al. (2012) [93].

#### Evolutionary history of Megaloptera

The order Megaloptera is inferred as monophyletic in all analyses and the family Corydalidae is also inferred as monophyletic. These results are congruent with the results of target DNA enrichment-based phylogenomic analyses of Neuropterida [20]. In addition, these results are in agreement with morphological analyses of genital and non-genital characters and with most morphology-based phylogenies of Neuropterida [9, 11, 27]. There are only few morphological autapomorphies of Megaloptera such as the shift of the bases of the male gonocoxites 9 to the base of tergum 9 [36]. Morphological characters supporting the monophyly of Corydalidae are scarce and they concern mostly genital characters and wing-base structures [27, 36]. Our taxon sampling does not allow further assessment of the monophyly of the corydalid subfamilies Corydalinae and Chauliodinae, but recent phylogenetic investigations have shown that the current taxonomic classification is supported by the analyses of molecular or morphological characters [20, 27, 36]. We estimated the common ancestor of extant Megaloptera to have lived in the early Triassic (~ 239 Mya), an estimate that is younger than estimates derived from analyses of target DNA enrichment data [20], but in agreement to the results of analyses of mitochondrial genomes [21].

#### Evolutionary history of Neuroptera

The order Neuroptera is inferred as monophyletic and our divergence time estimates suggest that its members started to diverge in the end of the Carboniferous (~ 301 Mya), while the common ancestor of the extant Neuroptera is estimated to have lived in the early Permian (~ 281 Mya). Our inferred phylogenetic trees corroborate the results of previous phylogenomic studies that suggested the family Coniopterygidae as sister to all remaining neuropteran families [20–22]. The idea that the dustywings are the sister group of the remaining families of Neuroptera is very old [94] and was originally based on a number of characters that this family shares with Megaloptera, such as the reduced number of Malpighian tubules (six in Coniopterygidae instead of eight in other Neuroptera) and the reduced number of abdominal ganglia of their larvae [94]. However, it should be noted that these features could be the result of miniaturization in the dustywings. Moreover, the alternative character states would be plesiomorphic, and therefore they constitute no arguments for monophyletic Neuroptera excluding Coniopterygidae. In our study Coniopterygidae is inferred as an ancient lacewing group that started to diversify in the middle of the Permian (~ 281 Mya). This result is in agreement with the findings of recent molecular dating analyses of Neuropterida [20, 21].

The phylogenetic placement of Coniopterygidae as sister to all remaining Neuroptera is in contrast with the majority of morphological analyses that have instead suggested Nevrorthidae as the most ancient lineage within the order [9, 11, 15]. The monophyly of Neuroptera with the exclusion of Nevrorthidae is morphologically supported by the formation of an undivided postmentum, the far-reaching modification or loss of the larval gula and the presence of cryptonephric Malpighian tubules of the larvae [15]. Specifically, in all terrestrial neuropteran larvae (including Coniopterygidae) the distal parts of the Malpighian tubules are connected with the colon, a phenomenon referred to as larval cryptonephry. In the aquatic larva of *Nevrorthus* all Malpighian tubules are free, while the aquatic larvae of Sisyridae have one cryptonephric tubule. The phenomenon of cryptonephry results in an improved water re-absorption mechanism and is apparently an adaptation to terrestrial environment, especially to a more exposed lifestyle and life in drier habitats. The original idea concerning the evolution of cryptonephry within Neuroptera is in contrast with the herewith presented phylogenetic relationships and with other molecular phylogenies [17, 20, 21], that suggest cryptonephry might be an apomorphic feature of Neuroptera with a putative secondary loss in Nevrothidae and secondary modification in Sisyridae. Despite the lack of morphological autapomorphies for a clade comprising Neuroptera excluding Coniopterygidae, this robust result across molecular analyses and methods suggests that a sister group relationship of Nevrorthidae to all other neuropteran families is unlikely.

A clade of Nevrorthidae, Sisyridae and Osmylidae (i.e. Osmyloidea) is inferred as sister to all remaining neuropteran families except Coniopterygidae and this clade is stable across analyses of different datasets and methods. This clade was also strongly supported in all quartet analyses, which in turn suggests that the placement of these three families in a monophyletic group is robust. This result is also in agreement with the results of analyses of target DNA enrichment data [20]. Potential synapomorphies of Osmyloidea are the semi-aquatic or aquatic larval ecologies and the secondarily multi-segmented antennae of the larvae [95]. Within Osmyloidea, a sister group relationship of Nevrorthidae and Sisyridae is congruent with the analyses of mitochondrial genomes [21] and with older studies based on the analysis of a few genes [17]. Moreover, a single shift to an aquatic lifestyle conforms to a branching pattern of Nevrorthidae and Sisyridae as sister clades. It should, however, be noted that the larvae of Nevrorthidae and Sisyridae have very different breathing and feeding adaptations, an observation that contrasts their sister group relationship [95]. The recent discovery of a complex submental gland with a multiporous opening in adults of *Nevrorthus* and *Osmylus* [8] could corroborate the monophyly of Osmylidae + Nevrorthidae as revealed by our summary coalescent analyses and by previous analyses of target DNA enrichment sequence data [20]. In the context of our best ML tree (Fig. 1), either the stem species of Neuroptera must have evolved this gland, with subsequent multiple losses, or it must have evolved in the stem species of Osmylidae + (Nevrorthidae + Sisyridae) and was then secondarily lost in Sisyridae. A clade of Osmylidae + Nevrorthidae has been presented elsewhere: e.g. by Zwick (1967) [96] (based on macrochaete of the neck, and the size of the palps), by Yang et al. (2012) [16] (mainly based on fossils), and in the recent target DNA enrichment-based phylogenomic study of Neuropterida [20]. Another interesting observation in this context is that the adults of Osmylidae are the only neuropterans with ocelli. Given that the possession of ocelli is most likely a plesiomorphic feature, as they are present in the adults of Raphidiidae and Corydalidae, we can hypothesize that the median eyes must have been reduced several times independently within Neuroptera, with possible vestiges still preserved in several groups.

A robust inference of the most archaic phylogenetic events within Neuroptera is essential for deciphering the evolution of lifestyle transitions of their larvae. Aquatic versus terrestrial habits of ancestral neuropteran larvae as well as a possible ancestral aquatic larvae of Neuropterida have been discussed in detail by authors of previous studies [20, 21]. Specifically, previous ancestral character state reconstructions (ACSR) of the larval ecologies of Neuropterida have suggested that the common ancestor of Neuroptera might have had aquatic larvae [20, 21]. Under the scenario of primarily aquatic neuropteran larvae, the results of our transcriptomic analysis would imply that the larvae of Coniopterygidae acquired terrestrial habits secondarily. In a second step Osmylidae must also have acquired terrestrial larvae independently, and finally in a third step the stem species of the remaining Neuroptera must also have acquired terrestrial larvae. Although three independent transitions to terrestrial lifestyle within Neuroptera is a possible scenario, it is not the most parsimonious. In an alternative scenario, with the stem species of Neuroptera being primarily terrestrial in the larval stages, the larvae of Sisyridae and Nevrorthidae would be secondarily aquatic as assumed by Gaumont (1976) [97]. Our parsimony-based ACSR of larval ecologies do not provide unequivocal support for either aquatic or terrestrial larvae in the last common ancestor of Neuroptera. In contrast, our SCM analyses unequivocally support primarily terrestrial larvae of Neuroptera and Neuropterida. However, it should be noted that parsimony-based ACSRs suffer from a number of limitations [98, 99] and that our parsimony-based analysis is based on a less extensive taxon sampling [95]. For these reasons we consider the estimates of SCM analyses as more reliable. The hypothesis of primarily terrestrial larvae of Neuropterida and Neuroptera suggests either two or three independent shifts to aquatic larval lifestyles within Neuropterida depending on the inferred topology within Osmyloidea. Interestingly, this hypothesis implies that the stem species of Megaloptera + Neuroptera had terrestrial larvae and that the larvae of Megaloptera are secondarily aquatic. We conclude from these observations that at least two shifts to aquatic habitats must have occurred in the early evolution of Neuropterida.

The family Dilaridae (pleasing lacewings) has been traditionally considered to form a clade with the families Mantispidae, Berothidae and Rhachiberothidae. The unofficial term “dilarid clade” has been used to describe this phylogenetic assemblage [9, 12, 15, 47]. We could not corroborate a clade that includes these four families as suggested by other authors [8, 47]. All analyses place Dilaridae as sister to all remaining Neuroptera except Coniopterygidae and Osmyloidea. This result is in accordance with previous sequenced-based phylogenomic analyses [20, 21]. Most importantly, the monophyly of the neuropteran families except Coniopterygidae and Osmyloidea is strongly supported by previous analyses of mitochondrial genomic rearrangements [18, 21].

Mantispidae and Berothidae were recovered as sister taxa with strong statistical branch support in all phylogenetic analyses, but the placement of this clade within Neuroptera is not robustly resolved. Concatenation-based and summary coalescent phylogenetic analyses of amino-acid sequences suggest a sister group relationship of Mantispoidea with Chysopidae. However, the different quartet analyses did not unequivocally support this sister group relationship. Our results corroborate previous views suggesting a close phylogenetic affinity of Berothidae and Mantispidae [9, 47]. Despite the fact that the family Rhachiberothidae is not included in our analyses, the monophyly of Mantispoidea is strongly supported by the presence of overlapping scales on antennae and maxillae, the presence of thoracic “trichobothria”, and by their hypermetamorphic development [9, 47]. The phylogenetic relationships within Mantispoidea, as well as the monophyly of Mantispidae, have remained unresolved [20], yet our taxon sampling does not allow testing any hypothesis concerning the phylogeny of Mantispoidea.

A clade Chrysopidae + Hemerobiidae, suggested by analyses of mitochondrial genomes [21, 22] and morphological characters [11], is not corroborated in our study. The conflicting phylogenetic hypotheses between the analyses of different data types presented here corroborate the results of Winterton et al. (2018) [20] concerning the affinities of Chrysopidae and Hemerobiidae. In their analyses of amino-acid sequence alignments Mantispoidea was inferred as sister to Chrysopidae, while Hemerobiidae was inferred as sister to Ithonidae + Myrmeleontiformia. These results are identical to our own results based on analyses of amino-acid sequence data. However, it should be noted that there is presently no morphological support in favor of these phylogenetic relationships. Morphological apomorphies shared by Hemerobiidae and Chrysopidae [11, 21] and the results of our quartet-based analyses show that the above-mentioned relationships require further scrutiny. The previously suggested clade Chrysopidae + Osmylidae that was based on analyses of larval head characters [9] is also not supported by our FcLM analyses. The main argument for this sister group relationship was based on length of the cardines, and the possession of special prothoracic glands [100]. However, varying lengths of the cardines are gradual modifications rather than discrete character states. Additionally, data on the prothoracic glands are missing for most neuropteran families. Therefore the arguments for a clade Chrysopidae + Osmylidae are not convincing.

The family Ithonidae is inferred as monophyletic and sister to monophyletic Myrmeleontiformia. The monophyly of Myrmeleontiformia is also strongly supported by our FcLM analyses and by previous analyses of morphological characters [14, 48]. The synapomorphies supporting the monophyly of Myrmeleontiformia, including the Psychopsidae, have already been documented by MacLeod (1964) [13], by Beutel et al. (2010) [15], and more recently by Badano et al. (2017) [14]. Overall, the larval cephalic morphology of Myrmeleontiformia differs profoundly from that of other groups of Neuroptera [15, 47], including among others the anterior shift of the tentorium and the greatly enlarged muscles of the paired mouthparts to handle the huge sucking tubes. Although Psychopsidae is not included in our study, we expected that if there is phylogenetic signal supporting a clade Ithonidae + Nymphidae, as suggested by other authors [20], the FcLM analyses would support this clade. Our phylogenetic analyses of amino-acid sequence alignments are in contrast with the results of the analyses of target DNA enrichment data that suggested paraphyletic Myrmeleontiformia in relation to Ithonidae [20, 24]. Interestingly, when we analyzed codon-based nucleotide sequences with all three codon positions included, Myrmeleontiformia was rendered paraphyletic in relation to Ithonidae similarly to the results of Winterton et al. (2018) [20]. The study of Winterton et al. (2018) [20] was the first molecular study to challenge the clade Myrmeleontiformia. In contrast, we received high statistical support in most phylogenetic analyses and in FcLM analyses in favor of the monophyly of this group.

Within Myrmeleontiformia (excluding Psychopsidae), Nymphidae is inferred as the earliest diverging lineage. Larval synapomorphies of Myrmeleontiformia excluding Psychopsidae are the conspicuously raised ocular region, a sensory pit on the apical labial palpomere, a strongly developed mid-dorsal cervical apodeme, a distinctly widened body posterior to the prothorax, and a compact and laterally rounded abdomen [15, 47]. The monophyly of the family Nemopteridae has been questioned before [101], but has been corroborated later [14]. We inferred Nemopteridae as monophyletic with strong statistical support and sister to a clade of Ascalaphidae + monophyletic Myrmeleontidae. These results are congruent with those of most recent cladistic analyses of Myrmeleontiformia based on analyses of larval characters [48]. However, non-parametric bootstrap support for the monophyly of Myrmeleontidae in the analyses of amino-acid sequence alignments was very low, and the same applies for the gene tree-based quartet support for this particular phylogenetic relationship. Previous phylogenomic analyses of the owlflies and antlions have suggested that Myrmeleontidae are polyphyletic with respect to Ascalaphidae [20, 24]. Based on that premise, it has been suggested that Ascalaphidae should be placed in a subfamily of Myrmeleontidae together with the antlion tribes Palparini, Dimarini and Stilbopterygini [24]. Since we did not recover Ascalaphidae nested within Myrmeleontidae, we retain the taxonomic status of Ascalaphidae as a separate family. The monophyly of the Myrmeleontidae has been corroborated based on several fossorial habits of their larvae and specific features linked with them [14, 48].

It is essential to mention that the different phylogenetic relationships of neuropteran families presented here corroborate previous results on the evolution of the larval gula-like sclerite within Neuroptera [20]. Winterton et al. (2018) [20] interpreted a pattern of evolution of the larval gula in Neuropterida according the results of their analyses. The result showed that the presence of gula is the ancestral state of the entire Neuropterida clade. As such, the presence of gula in the larvae of Nevrorthidae, Ithonidae, and Myrmeleontiformia could be formed either by numerous multiple losses in other lacewings, or could have at least two independent gains in these groups. When considering the larval gula in Myrmeleontiformia, this sclerite is usually reduced to a narrow sclerite medially dividing the two greatly enlarged genal sclerites, a structure that appears different from the gula in Megaloptera and Raphidioptera. Accordingly, the gula of Neuroptera is called “gula-like sclerite” by Winterton et al. (2018) [20] due to its likely non-homologous origin but contrary to the hypothesis of its homologous origin within Neuropterida implied by Aspöck (2002) [5]. Our parsimony-based character mapping analysis suggested an independent gain of the gula-like sclerite in the members of Ithonidae and Myrmeleontiformia similarly to the suggestion by Winterton et al. (2018) [20]. Because the herewith presented phylogenetic incongruencies mainly concern the phylogenetic position of Hemerobiidae, Chrysopidae and Mantispoidea and because the larvae of these groups lack a gula-like sclerite, the previously suggested pattern for the evolution of this morphological feature is unaffected by our results. Hence, an independent gain or reinvention of this gula-like sclerite in Ithonidae and in Myrmeleontiformia appears very likely.

## Conclusions

We draw four major conclusions from our analyses: (1) Part of the backbone tree of Neuropterida receives strong statistical support in several independent phylogenetic analyses and should be considered for now the most likely scenario of neuropterid evolution. One such scenario is the early split between Raphidioptera and Megaloptera + Neuroptera. Within Neuroptera, all analyses support an early split between Coniopterygidae and the remaining Neuroptera which cannot be corroborated with morphological analyses. The families Nevrorthidae, Sisyridae and Osmylidae form a monophyletic group sister to all other Neuroptera except Coniopterygidae. The family Dilaridae is the sister group to all remaining Neuroptera except Coniopterygidae and Osmyloidea. Despite these seemingly robust phylogenetic results, the phylogenetic relationships between the most species rich groups of Neuroptera (i.e. Chrysopidae, Ithonidae + Myrmeleontiformia, Hemerobiidae, Mantispoidea) are still not robustly resolved. For several branches in the neuropteran tree, the seemingly high branch support appears to be inflated and should be taken with caution. (2) Comparing concatenation versus summary coalescent approaches, and additional quartet-based measures of phylogenomic incongruence such as the FcLM approach, illustrates the potential of inflated branch support particularly derived from non-parametric resampling methods. Scientists are therefore advised to critically evaluate branch support in phylogenomic analyses and assume a conservative position. (3) The analyses of neuropterid relationships have received a lot of attention in the past and an extensive amount of phylogenomic data has been generated. However, parts of the backbone tree of Neuropterida can still not be robustly resolved which is disappointing, but reflecting a picture seen in other analyses of ancient phylogenetic splits as well. It will be necessary to invest molecular data beyond primary gene sequence information, for example structural genomic data [59, 102]. (4) Without an interplay of molecular and detailed morphological analyses, we will not be able to spot the major problems in biased results of any kind. Morphological analyses are critically needed to deliver a complete picture of the evolution of Neuropterida.

## Methods

### Taxon sampling

We sequenced and de novo assembled 88 whole-body transcriptomes of 85 species of Neuropterida (Raphidioptera: 18 species, Megaloptera: seven species, Neuroptera: 60 species, Additional file 1: Table S6), comprising representatives of all extant families of Neuropterida except Rhachiberothidae and Psychopsidae. For the species *Parvoraphidia microstigma*, *Palpares libelluloides*, *Peyerimhoffina gracilis*, two transcript libraries of separate specimens were generated respectively, sequenced and assembled (Suppl Table 1). RNA isolation, RNA library preparation, transcriptome sequencing, transcriptome assembly, and transcriptome quality assessment were performed according to the procedures described by Misof et al. (2014) [4] and by Peters et al. (2017) [103] (see Additional file 2). We complemented our dataset with publicly available transcriptomic and genomic (official gene sets, OGS) sequence data of eight neuropterid and 41 outgroup species, representing all currently recognized holometabolous insect orders (Additional file 1: Table S7). In total, our sampling comprised 96 transcriptomes of Neuropterida (from 92 species) and 45 transcriptomes and official gene-sets of non-neuropterid insects (from 41 species, see Additional file 2).

### Orthology assignment, multiple sequence alignment, alignment refinement and alignment masking

We identified a set of 3983 clusters of orthologous single-copy genes (COGs) at the hierarchical level “Endopterygota” (i.e. Holometabola), based on a custom profile query in OrthoDB7 [104] (see Additional file 2 for details). The custom query allowed COGs only to be included in the ortholog set if single-copy genes of all selected reference taxa were present in a given COG. As reference genomes, we selected *Acromyrmex echinatior* v. 3.8 [105], *Tribolium castaneum* v. 3.0 [106], *Bombyx mori* v. 2.0 [107], and *Drosophila melanogaster* v. 5.51 [108] (see Additional file 1: Table S8).

Mapping of putative orthologous transcripts to each COG, at the translational (amino-acid, aaCOGs) and at the transcriptional level (nucleotide, nCOGs), was performed with the software package Orthograph v. 0.5 [109] (see Additional file 2). Subsequently, we selected a subset of outgroup and ingroup species with a high number of assigned orthologs for downstream analyses (Additional file 1: Table S1). Specifically, if more than one transcriptome/OGS were processed from the same outgroup or ingroup species, the dataset with the highest number of identified orthologs was included in downstream analyses. We did not exclude ingroup taxa based on their completeness (measured by the number of assigned orthologs), except in those cases in which more than one transcriptome from the same species were used in the orthology assignment step. Overall, we considered transcriptomes of the outgroup species to be of high completeness when putative orthologous transcripts from these datasets were assigned to at least 3000 COGs (Additional file 1: Table S1, with the exception of *Mengenilla moldrzyki*). The filtered dataset consisted of 124 species (92 neuropterid species and 32 outgroup species) including the four reference species of the ortholog set.

Orthologous amino-acid sequences were aligned with MAFFT v. 7.123 [110] and by applying the L-INS-i algorithm. We followed the procedures outlined by Misof et al. (2014) [4] for identifying potentially non-orthologous and misaligned sequences. Details on the applied alignment-refinement procedure, the removal of putative outliers, and the generation of codon-based alignments (corresponding to the amino-acid alignments) are given in Additional file 2. Based on the rationale of previous phylogenomic studies employing various alignment masking (i.e. alignment-column filtering) methods [4, 61, 111–118] we used ALISCORE v. 1.2 [119, 120], to identify and mask putatively randomly similar aligned sections at the amino-acid sequence level and also masked the corresponding nucleotide sequence codons.

### Concatenation of supermatrices

We combined the results of alignment masking and protein-domain identification (see Suppl. Text 1) to generate amino-acid and nucleotide sequence supermatrices partitioned according to protein-domain clans, families and single domains following the procedure described by Misof et al. (2014) [4]. Subsequently, we generated subsets of the original concatenated supermatrix to improve data coverage and information content, and to assess any putative effects of violations of the SRH conditions assumed by the substitution models in our phylogenetic analyses (Table 1, Additional file 2). For each amino-acid supermatrix, we calculated the overall alignment completeness scores and generated heatmaps of pairwise completeness scores with AliStat v. 1.6 (current version available from: https://github.com/thomaskf/AliStat) [121]. Overall deviation from SRH conditions within each supermatrix [122] was measured with the Bowker’s test of symmetry [123] and by generating heatmaps as implemented in SymTest v. 2.0.47 (current version available from: https://github.com/ottmi/symtest, see Misof et al. 2014 [4]).

### Phylogenetic analyses of amino-acid sequence data partitioned according to protein-domain clans and families, and to single protein domains

We selected the amino-acid supermatrix E (Table 1, Additional file 1: Table S9, details in Additional file 2) for downstream analyses, because it showed increased phylogenetic information content and data coverage compared to the supermatrices A, B, C, and D, while being only slightly less informative and larger than supermatrix F (Table 1) [121, 124]. We used PartitionFinder v. 2.0.0-pre11 [125] to identify the optimal combination of partitions into meta-partitions, and to infer the respective amino-acid substitution models for each meta-partition prior to tree reconstructions (Additional file 2). The resulting partitioning scheme with the best AICc and the accompanying selected models for each meta-partition were used as input for IQ-TREE v. 1.3.13 [126] to conduct 100 independent maximum likelihood tree searches (see Additional file 2). We selected the tree with the highest log-likelihood score among all tree searches as the maximum likelihood tree (best ML tree).

Based on the best ML tree, we calculated branch support from 100 non-parametric bootstrap replicates as well as from 10,000 replicates of the SH-like approximate likelihood ratio test (SH-aLRT) [127] with IQ-TREE v. 1.3.13. We assessed whether or not the number of bootstrap replicates was sufficient to accurately infer branch support by running the a posteriori bootstop test in RAxML v. 8.2.8 [128, 129] and by doing ten independent tests with different random seeds (see Additional file 2). We calculated an additional branch-support metric by applying the bootstrap by transfer support measure based on our calculated bootstrap trees [130]. We also tested for the presence of rogue taxa in our dataset with RogueNaRok v. 1.0 [131]. Finally, we rooted the presented tree (Fig. 1) by selecting the split between Hymenoptera and all remaining holometabolous taxa using the software Seaview v. 4.5.4 [132].

Modeling site-heterogeneous processes of amino-acid substitutions by incorporating site specific amino-acid profiles into phylogenetic reconstruction can potentially alleviate phylogenetic artifacts due to model miss-specification [133–135]. We therefore performed an additional tree search on supermatrix E with the PMSF mixture model implemented in IQ-TREE v. 1.5.5 [136] (Additional file 2) and compared results of this phylogenetic reconstruction with those described above. In order to control for the effects of missing data, we generated two reduced versions of supermatrix E by keeping only those alignment sites with at least 90% or 95% of the total number of species present (207,582 and 110,708 amino-acid alignment sites respectively). For each of these two reduced matrices, we conducted two additional tree searches with the rapid approximation to the PMSF model in IQ-TREE v. 1.5.5 (see Additional file 2 for details).

Heterogeneous amino-acid composition among species in the dataset can severely bias phylogenetic reconstructions due to violation of substitution model assumptions [80, 122, 137–140]. We therefore controlled for among-species compositional heterogeneity in the analyzed amino-acid supermatrix E by masking subsets with a relative composition frequency variation (RCFV) value greater than or equal to 0.1 [113, 141], calculated with BaCoCa v. 1.01 [142]. We monitored the effect of this masking by applying the Bowker’s symmetry tests across taxa with SymTest v. 2.0.47. With this RCFV-corrected dataset we conducted five ML tree searches with IQ-TREE v. 1.6.6 by specifying the previously estimated most-fitted substitution models for each meta-partition. We calculated 1000 ultrafast bootstraps (UFB) [143] and 10,000 SH-aLRT replicates for the RCFV-corrected dataset with IQ-TREE v. 1.6.6 (see Additional file 2).

We studied the effect of potentially confounding signal, like non-random distribution of data coverage and violations of SRH conditions, on our phylogenetic reconstructions with the FcLM approach [144] as described by Misof et al. (2014) [4]. We formulated nine phylogenetic hypotheses, that are in part based on the results of our tree reconstructions and partly on published alternative phylogenetic hypotheses. For each of the nine tested hypotheses (Additional file 1: Table S2), we used a permutation approach to assess signal originating from non-random distribution of data coverage and violations of SRH conditions in supermatrix E. Accompanying the FcLM approach, we generated a decisive subset of supermatrix E (Table 1) [61], and which included only meta-partitions with 1) data for all species, 2) less than 30% ambiguous sites (< 30% of X/−), and 3) an alignment length of at least 500 amino-acid sites. The selected meta-partitions were concatenated into a decisive supermatrix (209 meta-partitions, 228,933 aligned amino-acid sites) with FASconCAT-G v. 1.02 [145]. The phylogenetic analyses of this decisive supermatrix followed the scheme of the previous analyses (Additional file 2).

### Concatenation-based phylogenetic analyses of the second codon positions

We compared the results of tree reconstructions based on data at the amino-acid and nucleotide sequence levels. Substitutions at the nucleotide sequence level follow different processes than substitutions at the amino-acid sequence level, and thus the analyses at the nucleotide level can be considered an independent test of the results based on the amino-acid sequence data. Published investigations have consistently demonstrated that the base composition of second codon positions of protein-coding nucleotide sequences are the most homogeneous across taxa and thus least violate assumptions of the applied nucleotide substitution models [4, 80, 146]. We therefore selected the nucleotide supermatrix corresponding to the amino-acid supermatrix D (Table 1) and evaluated the degree of deviation from SRH conditions on different subsets of this matrix [137, 138]. We performed the pairwise symmetry tests of homogeneity, by selecting the Bowker’s test in SymTest v. 2.0.47, on the following datasets: 1) the entire nucleotide supermatrix, 2) only first codon positions of the nucleotide supermatrix 3) only third codon positions of the nucleotide supermatrix, and 4) only second codon positions of the nucleotide sequence supermatrix. Since the second codon positions showed the least deviation from the SRH conditions, we masked all first and third codon positions and further proceeded by analyzing a dataset composed exclusively of second codon positions. We calculated the most appropriate partitioning scheme to analyze the second codon positions of supermatrix D, with the *k*-means algorithm [147] in PartitionFinder v. 2.0.0-pre11, and conducted 100 independent maximum likelihood searches with IQ-TREE v. 1.3.13 (details in Additional file 2). We calculated branch support values from 100 non-parametric bootstraps and 100 TBE replicates and mapped them onto the tree with the highest log-likelihood among all tree searches.

### Concatenation-based vs. summary coalescent phylogenetic analyses of gene partitions

The concatenation approach has been criticized for being ignorant against gene tree discordance due to ILS and thus for being susceptible to tree reconstruction biases caused by these effects [74, 76, 148, 149]. Currently it is unclear which approach delivers the most reliable topological estimates when analyzing empirical data [76, 149–156]. To explore the sensitivity of our supermatrix-based analyses to the putative effects of gene tree discordance we used the 3983 alignments of COGs to conduct summary coalescent analyses with ASTRAL III v. 5.6.1 [157]. We first removed ambiguous-only sites (X, N, −) from each amino-acid and nucleotide sequence alignment. Subsequently, we used ModelFinder in IQ-TREE v. 1.6.3 [158] to infer the best fitting substitution model for each gene separately at the translational level and the transcriptional level (see Additional file 2 for details) based on the BIC criterion. We considered all combinations of modelling ASRV. At the nucleotide sequence level all three codon positions for each gene were included in the phylogenetic analyses. We performed ten independent ML tree searches for each gene with the respective best fitting model and selected the best ML gene tree among these searches to be used for the summary coalescent analyses. Coalescent-based species trees were inferred separately at the amino-acid and the nucleotide sequence levels. The resulting species trees were then scored and annotated by comparing the gene trees with the inferred species tree [66]. We considered the quartet support values of the summary coalescent analyses (q1, q2, q3) complementary to our FcLM analyses for assessing the conflict in our dataset (Fig. 2a; note that the coalescent method does not test for putative confounding signal per se). It has been suggested that low data coverage may have a negative impact on summary coalescent methods [152]. In order to account for this negative effect, we selected only these gene partitions with at least 95% species coverage (min. = 115 leaf terminals, 2083 genes) and repeated coalescent species tree analyses both at the amino-acid and nucleotide sequence levels. Finally, results of the different coalescent analyses were compared to those based on domain-based-partitioned and gene-based partitioned concatenated supermatrices (see Additional file 2 for details). We used ETE v. 3.0 [159] to visualize quartet support, as an indication of gene tree conflict, on the species trees that were inferred with ASTRAL (e.g. Fig. 2a).

### Estimation of divergence times of Neuropterida

We used 129 meta-partitions of the decisive amino-acid supermatrix (supermatrix E-Decisive, see Additional file 2 and Table 1) to estimate the divergence times of the major lineages of Neuropterida based on 12 fossil calibrations (Additional file 1: Table S10). The fossil calibrations were selected according to the criteria described by Parham et al. (2012) [160] (see Additional file 2). We extracted the 129 meta-partitions from the decisive supermatrix and re-estimated the most suitable substitution models for each individual meta-partition using IQ-TREE v. 1.6.6 (with the AICc criterion), by restricting model selection to a set of amino-acid substitution matrices available in the PAML package [161] (JTT + G, LG + G, WAG + G, DAYHOFF + G, JTTDCMUT + G, DCMUT + G) and by using the fixed topology of the best ML tree. Subsequently, substitution rates per time unit for each meta-partition were estimated with codeml v. 4.9e (part of the PAML software suite) under the assumption of a strict clock (clock = 1), and by using the fixed topology of the best ML tree and the above-selected substitution models. The age of the root was fixed at 362.35 million years ago (Mya) in each ML analysis. This root age was derived as the average between the oldest known hexapod fossils at 411 Mya and the minimum age 313.7 Mya for Aparaglossata [162] (i.e. Holometabola without Hymenoptera, see Peters et al. 2014 [2]). The purpose of these analyses was to calculate a rough estimate of the mean rate prior for each meta-partition to be used for estimating the divergence times in MCMCTree v. 4.9e (part of the PAML software suite [161]).

Calculation of the Hessian matrices followed the standard procedure, applying the fitted substitution models (+ G with four rate categories) for each meta-partition (Additional file 2). Similarly to the approach proposed by Misof et al. (2014) [4], divergence time estimation was performed for each of the 129 meta-partitions separately with the approximate likelihood method [163]. We used the same set of calibration points (Additional file 1: Table S10), the independent-rates model [164] and the topology of the best ML tree for each separate analysis. The estimated substitution rate of each meta-partition was used as the mean (μ) of the Dirichlet-gamma prior (rgene_gamma) in MCMCTree v. 4.9e. We specified a hard maximum bound for the age of the root at 411 Mya in all analyses and ran each MCMCTree chain for 550,000 generations, sampling every 10th generation and discarding the first 50,000 samples as a burn-in (Additional file 2). For each meta-partition, three different analyses were performed:

1. Two independent analyses (run 1 and run 2) with the same calibrations and diffuse rate priors (α = 2) to check for repeatability of the analyses (Additional file 2).
2. One calibration without data (usedata = 0) to assess whether or not the results without data were significantly different, implying that the data harbor sufficient information for reliably estimating divergence times.

For each of the three separate analyses (two analyses with data and one without) parameter outputs of the separate analyses of the meta-partitions were combined in a single MCMC summarized file. We mapped the posterior mean node ages and 95% confident intervals (equal-tail CI) on the overall best ML tree (Fig. 1). The branch lengths of the resulting chronogram were calculated as the posterior mean node-age difference between two nodes. The posterior node-age estimates from the 129 meta-partitions were used to calculate median posterior node-age estimates in R v. 3.4.3 [165] (Fig. 3, Additional file 2). The datasets used for estimation of divergence times and all the analyzed supermatrices are deposited in the Dryad repository [166].

### Tracing the evolution of larval characters within Neuropterida

In addition to the informal discussion of implications of the proposed phylogeny for our understanding of the evolution of neuropterid insects in general, we also formally analyzed character transformations (Fig. 1) with Mesquite v. 3.2 [167]. For this analysis we selected a data matrix comprising 86 larval characters from a previously published morphological study with focus on Neuroptera [95] (see also Beutel et al. 2010 [15] and Jandausch et al. 2018 [47]). We analyzed this character matrix under the constrained topology of our best ML tree (Fig. 1) using maximum parsimony (see Additional file 4). A summary of the interpretation of results for the most important characters is provided in Additional file 1: Table S5.

Previous ACSRs of the larval ecologies of Neuropterida have suggested that ancestral Neuropterida most likely had an aquatic larva [20, 21]. However, a clade of Nevrorthidae + Sisyridae as sister to Osmylidae has not been inferred in previous phylogenomic studies, and the taxon sampling of outgroup species was not as extensive as in our study [20, 21]. Therefore, we additionally used a Bayesian approach to reconstruct the ancestral states of larval ecologies of Neuropterida. Specifically, we used the stochastic character mapping method (SCM) [98, 99], as implemented in the R package phytools v. 0.6.99 [168] (see Additional file 2 for details and additional sensitivity analyses). We simulated 10,000 character histories conditioned on the topology and branch lengths of the best ML tree (Fig. 1), and by using the best fitted model of character evolution. The results of the SCM analyses were visualized using ape v. 5.3 [169].

## Supplementary information

**Additional file 1: Supplementary Tables S1–S20.** The supplementary tables include: 1) descriptive statistics of the results of orthology assignment, 2) results of FcLM analyses, 3) summarized results of the divergence times estimates as resulted from the different runs (MCMC chains) and fossil calibrations (see also Additional file 2), 4) results of the character mapping analysis under the transcriptomic pattern of phylogeny, 5) descriptive statistics of newly sequenced and previously published transcriptomic and genomic data, 6) overview of the official gene sets used in the orthology assignment step, 7) descriptions of analyzed supermatrices (see Additional file 2), 8) list of fossil calibrations used for divergence time estimation, 9) summarized statistics of the results of the alignment refinement, 10) branch support statistics inferred from the summary coalescent phylogenetic analyses, 11) results of model-selection for the analyses of evolution of larval ecologies.

**Additional file 2: Supplementary experimental procedures and results**. This file contains supplementary methodological procedures and results that are not provided in detail in the “Results” and “Methods” sections of the research article.

**Additional file 3: Supplementary Figures S1–S56.** The supplementary figures include: 1) all phylogenetic trees inferred from the analyses of different datasets and tree-inference methods, 2) results of additional ACSR analyses under different parameters, 3) heatmaps visualizing the pairwise alignment completeness scores of all analyzed supermatrices, 4) heatmaps visualizing the pairwise deviation from SRH conditions in each analyzed supermatrix, 5) scatter plot of the mean posterior node-age estimates from run 1 plotted against the mean posterior node-age estimates from run 2 when using all fossil calibrations, 6) beanplots of median posterior node-age estimates from run 1 and from run 2 when using all fossil calibrations, 7) scatter plots of the mean posterior node-age estimates plotted against the 95% higher posterior density CI-width of each node when running the dating analyses with or without data.

**Additional file 4.** Character matrix with coded states for 86 larval characters of Neuroptera. This is the nexus-formatted character matrix that was used for the parsimony-based analysis of the evolution of larval characters. Taxon sampling is the same as in Jandausch et al. (2018, 2019) [47, 95].

## Data Availability

The datasets and additional information supporting the conclusions of this article are available in the Dryad digital repository, 10.5061/dryad.1jwstqjrs.

## Abbreviations

AICc: Corrected Akaike information criterion
ACSR: Ancestral character state reconstruction,
ASRV: Among-site rate variation
BIC: Bayesian information criterion
COG: Cluster of orthologous single-copy genes
ER: Equal-rates model
FcLM: Four-cluster likelihood mapping
ILS: Incomplete lineage sorting
MCMC: Markov chain Monte Carlo
ML: Maximum likelihood
Mya: Million years ago
OGS: Official gene set
PMSF: Posterior mean site frequency profile
RCFV: Relative composition frequency variation
SCM: Stochastic character mapping
SH-aLRT: SH-like approximate likelihood ratio test
SRH: Global stationary (time-) reversible and homogeneous conditions
TBE: Bootstrap support by transfer
UFB: Ultrafast bootstrap
