# Correction to: An integrative phylogenomic approach to elucidate the evolutionary history and divergence times of Neuropterida (Insecta: Holometabola)

**DOI:** 10.1186/s12862-020-01695-4

**Published:** 2020-10-15

**Authors:** Alexandros Vasilikopoulos, Bernhard Misof, Karen Meusemann, Doria Lieberz, Tomáš Flouri, Rolf G. Beutel, Oliver Niehuis, Torsten Wappler, Jes Rust, Ralph S. Peters, Alexander Donath, Lars Podsiadlowski, Christoph Mayer, Daniela Bartel, Alexander Böhm, Shanlin Liu, Paschalia Kapli, Carola Greve, James E. Jepson, Xingyue Liu, Xin Zhou, Horst Aspöck, Ulrike Aspöck

**Affiliations:** 1 Centre for Molecular Biodiversity Research, Zoological Research Museum Alexander Koenig, 53113 Bonn, Germany; 2 Department of Evolutionary Biology and Ecology, Institute of Biology I (Zoology), Albert-Ludwigs-Universität Freiburg, 79104 Freiburg, Germany; 3 Australian National Insect Collection, National Research Collections Australia, Commonwealth Scientific and Industrial Research Organisation (CSIRO), Canberra, ACT 2601 Australia; 4 Department of Genetics, Evolution and Environment, University College London, London, WC1E 6BT UK; 5 Institut für Zoologie und Evolutionsforschung, Friedrich-Schiller-Universität Jena, 07743 Jena, Germany; 6 Natural History Department, Hessisches Landesmuseum Darmstadt, 64283 Darmstadt, Germany; 7 Steinmann-Institut für Geologie, Mineralogie und Paläontologie, Rheinische Friedrich-Wilhelms-Universität Bonn, 53115 Bonn, Germany; 8 Centre for Taxonomy and Evolutionary Research, Arthropoda Department, Zoological Research Museum Alexander Koenig, 53113 Bonn, Germany; 9 Department of Evolutionary Biology, University of Vienna, 1090 Vienna, Austria; 10 Department of Entomology, China Agricultural University, 100193 Beijing, People’s Republic of China; 11 LOEWE Centre for Translational Biodiversity Genomics (LOEWE-TBG), 60325 Frankfurt, Germany; 12 School of Biological, Earth and Environmental Sciences, University College Cork, Distillery Fields, North Mall, T23 N73K Cork, Ireland; 13 Institute of Specific Prophylaxis and Tropical Medicine, Medical Parasitology, Medical University of Vienna (MUW), 1090 Vienna, Austria; 14 Zoological Department II, Natural History Museum of Vienna, 1010 Vienna, Austria

**Correction to: BMC Evol Biol 20, 64 (2020)**

**https://doi.org/10.1186/s12862-020-01631-6**

Following publication of the original article [1], the authors discovered that some pie charts had been misplaced in the tree of Fig. 2a, and in the trees of supplementary figures S16, S22, S24 (Additional file 3) due to incorrect visualization of the output of ASTRAL [2]. These quartet support values are, however, correctly provided in supplementary tables S16–S19 of the original publication. Thus, the original publication is inconsistent on a couple of support values for specific phylogenetic relationships. We provide now a corrected Fig. 2 and an updated Additional file 3 with corrected Figures S16, S22, S24. The changes in Fig. 2a do not affect the controversial branches discussed in the original publication (e.g. relationships within Osmyloidea, phylogenetic affinities of Chrysopidae and Hemerobiidae, monophyly of Myrmeleontiformia and Myrmeleontidae). The corrected Fig. 2a shows the correct (increased) quartet support for the monophyly of Neuropterida (q1 = 0.798, q2 = 0.098, q3 = 0.103) and the correct (lower) quartet support for the monophyly of Neuroptera (q1 = 0.476, q2 = 0.168, q3 = 0.357). Quartet support for the monophyly of Neuroptera (q1) is still higher in comparison to the other two topologies (q2, q3) around this specific internode. Therefore these changes do not alter the conclusions of [1]. The authors would like to apologize for any inconvenience caused.

**Fig. 2:**
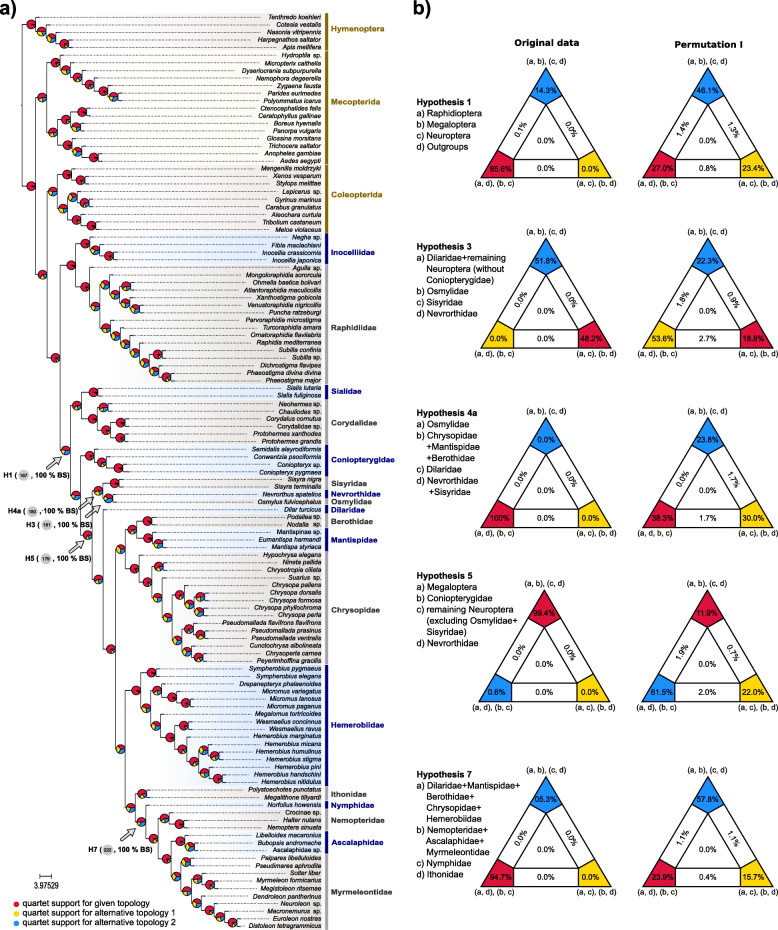
Gene tree-based and concatenation-based quartet analyses of the phylogenetic relationships of Neuropterida. **a** Phylogenetic relationships of Neuropterida, as they resulted from the summary coalescent phylogenetic analysis with ASTRAL, when analyzing the full set of gene trees (3983 gene trees inferred at the amino-acid sequence level). Pie charts on branches show ASTRAL quartet support (quartet-based frequencies of alternative quadripartition topologies around a given internode). Arrows indicate the numbers of the corresponding tree nodes in Fig. 1, and the corresponding hypotheses in the FcLM analyses. **b** Results of FcLM analyses for a selection of phylogenetic hypotheses applied at the amino-acid sequence level (supermatrix E). The first column shows the results of FcLM when the original data of supermatrix E were analyzed. The second column shows the results of FcLM after phylogenetic signal had been eliminated from supermatrix E (i.e. permutation no. I, see Additional file 2)

## Supplementary information

**Additional file 3: Supplementary Figures S1–S56.** The supplementary figures include: 1) all phylogenetic trees inferred from the analyses of different datasets and tree-inference methods, 2) results of additional ACSR analyses under different parameters, 3) heatmaps visualizing the pairwise alignment completeness scores of all analyzed supermatrices, 4) heatmaps visualizing the pairwise deviation from SRH conditions in each analyzed supermatrix, 5) scatter plot of the mean posterior node-age estimates from run 1 plotted against the mean posterior node-age estimates from run 2 when using all fossil calibrations, 6) beanplots of median posterior node-age estimates from run 1 and from run 2 when using all fossil calibrations, 7) scatter plots of the mean posterior node-age estimates plotted against the 95% higher posterior density CI-width of each node when running the dating analyses with or without data.

## Abbreviations

*ACSR*: Ancestral character state reconstruction
FcLM: Four-cluster likelihood mapping
SRH: Global stationary (time-) reversible and homogeneous conditions
