# Supplementary material for: An integrative phylogenomic approach to elucidate the evolutionary history and divergence times of Neuropterida (Insecta: Holometabola)
Source: BMC Evol Biol. 2020 Jun 3;20:64. doi: 10.1186/s12862-020-01631-6 (PMC7268685; doi:10.1186/s12862-020-01631-6)
Supplement: Supplementary file 2 — Additional file 2: Supplementary experimental procedures and results. This file contains supplementary methodological procedures and results that are not provided in detail in the “Results” and “Methods” sections of the research article. [file 12862_2020_1631_MOESM2_ESM.pdf]

Additional file 2

Supplementary experimental procedures and results to the research article:

An integrative phylogenomic approach to elucidate the evolutionary history and divergence times of Neuropterida (Insecta: Holometabola)

Alexandros Vasilikopoulos, Bernhard Misof, Karen Meusemann, Doria Lieberz, Tomáš Flouri, Rolf G. Beutel, Oliver Niehuis, Torsten Wappler, Jes Rust, Ralph S. Peters, Alexander Donath, Lars Podsiadlowski, Christoph Mayer, Daniela Bartel, Alexander Böhm, Shanlin Liu, Paschalia Kapli, Carola Greve, James E. Jepson, Xingyue Liu, Xin Zhou, Horst Aspöck, Ulrike Aspöck

1. Supplementary experimental procedures

1.1 Specimen collection and preservation, transcriptome sequencing, transcriptome assembly, Univec and cross-contamination screening

Specimen collection and preservation was performed according to the procedures described by Peters et al. (2017) [1]. Specimen vouchers, if available, were deposited at ZFMK (Bonn, Germany). We followed the procedures described by Misof et al. (2014) [2] and by Peters et al. (2017) [1] for RNA extraction and RNA library preparation. The generated transcript libraries were paired-end sequenced with the Illumina HiSeq technology (2000 or 4000, see the Additional file 1: Table S6 for details). Transcriptome assembly followed the procedures described by Peters et al. (2017) [1]. In brief, the raw reads from each sequenced transcriptome library were quality filtered and trimmed, and subsequently used for *de novo* transcriptome assembly. Transcript raw reads were assembled in BGI-Shenzhen (China) using SOAPdenovo-Trans-31kmer (v. 1.01) [3]. Subsequently, we followed the procedures described by Peters et al. (2017) [1] for removing vector and adapter contaminations and for identifying cross-contamination from other 1KITE (http://www.1kite.org/) transcriptomes. Information on how many transcripts were removed from each transcript library is provided in Additional file 1: Table S6. Raw data were submitted to the NCBI Short Read Archive (SRA) and the filtered assemblies were submitted to the NCBI-TSA database and were screened for further contamination by NCBI. Detailed assembly statistics and Gene-bank accession numbers of the *de novo* sequenced transcriptomes are given in Additional file 1: Table S6.

1.2 Exploitation of previously published transcriptomic and genomic datasets

We complemented our newly sequenced transcriptomes with previously published transcriptomic and genomic data. Specifically, seven neuropterid transcriptomes previously

published by the 1KITE consortium [2] (updated 1KITE assemblies, for details see Peters et al. 2017 [1]), and one non-1KITE neuropterid transcriptome (referring to the species *Sialis lutaria*) were added to the initial dataset (Additional file 1: Table S7). We furthermore included 45 previously published transcriptomes and/or official gene sets (OGSs) from 41 outgroup species covering every holometabolous insect order (Additional file 1: Table S7) [2, 4–6]. Note that we used two different OGS versions of the species *Nasonia vitripennis* and *Aedes aegypti* in the orthology assignment step (see section 1.4), in order to be able to select among different datasets on the basis of their completeness for downstream analyses (Additional file 1: Table S7). For the same reason two different datasets of the species *Glossina morsitans* were used in the orthology assignment step: one published transcriptome and one nucleotide OGS (nucleotide OGS with coding sequences, Additional file 1: Table S1).

1.3 Preparation of the ortholog gene sets

We searched OrthoDB v. 7 [7] (http://cegg.unige.ch/orthodb7, access: April 2015) for genes that are single-copy and orthologous across holometabolous insects. Specifically we selected the "Endopterygota" (i.e. Holometabola) node and demanded the presence of only a single gene per ortholog group in the in the genomes of each of the four reference species with well-sequenced and annotated genomes: *Acromyrmex echinatior* v. 3.8 [8], *Tribolium castaneum* v. 3.0 [9], *Bombyx mori* v. 2.0 [10], and *Drosophila melanogaster* v. 5.51 [11]. We did not restrict the number of gene copies in the remaining holometabolous genomes. The search for the above mentioned criteria returned 3,983 ortholog groups (i.e. clusters of orthologous and single-copy genes, COGs [12]), which were downloaded from OrthoDB v. 7. We subsequently obtained the official gene sets (OGSs) of the reference species (same versions as the ones used by OrthoDB) at the amino-acid and the

nucleotide sequence level from the respective genome databases (see Additional file 1: Table S8, [6, 13–15]. The OGS at the protein level of *Drosophila melanogaster* was downloaded from the OrthoDB repository. The file with the coding sequences (CDS file) was then adjusted to the protein OGS file, by removing all additional sequences in the CDS file with no corresponding ID in the OrthoDB protein file.

All OGSs were modified by the use of custom-made Perl scripts so that: (1) no duplicate headers exist in the OGSs files (2) for those genes that were part of the COGs, only one isoform/splice variant of the same gene was present in the OGSs files (we retained the isoform that was used by OrthoDB for inferring *de novo* orthology) (3) amino-acid sequences corresponded perfectly to the nucleotide sequences, (4) 5' and 3' untranslated regions are not part of the transcripts. Sequences that could not be modified in a way so that they correspond between nucleotide and protein OGS files were excluded from both OGSs. The OrthoDB list (COGs) was filtered so that it contained only entries of the reference species. All information with respect to reference taxa, and the no. of sequences in each reference taxon OGS file is provided in Additional file 1: Table S8.

1.4 Transcript orthology assignment and taxon filtering for downstream analyses

We used Orthograph v. 0.5 (May 2015 or later) [16] to assign coding sequences from each transcriptome (or OGS) to the 3,983 ortholog groups with the following settings: strict-search=0, max-blast-searches = 100, max-blast-hits = 100, orf-overlap-minimum = 0.5, extend-orf = 1, minimum-transcript-length = 30, substitute-u-with = X, extend-orf = 1, frameshift-correction = 1. Following the orthology assignment procedure, we filtered taxa in our dataset based on the total number of orthologs assigned to each transcriptome (see methods of the main text). Filtering of the outgroup taxa was done in way so that the

retained outgroup species cover all major lineages within Holometabola. Although, a comparatively small number of transcripts of *Agulla* sp., *Halter nutans*, *Semidalis aleyrodiformis,* and *Coniopteryx pygmaea* were assigned to the 3,983 ortholog groups (i.e. less than 3,000, see Additional file 1: Table S1), we included these species in our phylogenetic analyses, because we deemed these taxa important for inferring the interfamiliar phylogenetic relationships of Neuropterida. A summary of the removed taxa after the filtering step is given in the column "not included" in Additional file 1: Table S1.

1.5 Alignment refinement, alignment masking and protein domain identification

All transcript sequences that were still identified as outliers after the alignment refinement step (see methods of the main text) were finally removed from the amino-acid multiple sequence alignments and their corresponding nucleotide sequence files. After this step we also removed all sequences of the reference species *Drosophila melanogaster*, *Bombyx mori*, and *Acromyrmex echinatior,* reducing the total number of species for downstream analyses to 121. For generating corresponding codon-based nucleotide alignments, we used a modified version of PAL2NAL [17] as described by Misof et al. (2014) [2] with the amino-acid multiple sequence alignments as blueprint. Identification of alignment sections of random similarity and noise was performed with ALISCORE v. 1.2 (options: -r 10^{27} for comparing all possible pairs of sequences, -e for gappy RNAseq data, and otherwise default settings) [18, 19] for each amino-acid multiple sequence alignment separately. We then generated corresponding lists for nucleotide alignments using custom-made Perl scripts.

Based on the rationale that domain-based partitioning of amino-acid sequence data delivers partitioning schemes with higher AICc scores than partitioning according to genes

[2], we used Pfam v. 27 [20] and the script Pfam_Scan.pl (v. 1.5, ftp://ftp.ebi.ac.uk/pub/databases/Pfam/Tools/, downloaded in December 2015) to identify protein domain clans, families, single domains and void regions (i.e. not annotated regions) within each amino-acid sequence alignment. We then generated a protein-domain partitioned amino-acid supermatrix (supermatrix A, Additional file 1: Table S9) as well as the corresponding codon-based nucleotide supermatrix with custom-made Perl scripts (by merging results of protein-domain identification and alignment masking; the procedures for merging these results are described by Misof et al. (2014) [2]).

1.6 Supermatrix concatenation, evaluation and optimization

1.6.1 Concatenation of amino-acid sequence datasets

Starting with supermatrix A, we generated six different amino-acid supermatrices optimized by applying different strategies and evaluated their suitability for phylogenetic tree inference (see Table 1 of the main text, Additional file 1: Table S9). We evaluated (1) the overall information content of these supermatrices, as implemented in the software MARE [21], (2) each matrix's saturation in terms of presence and absence of partitions [21], and (3) the overall alignment completeness scores calculated with AliStat v. 1.6 (see Misof et al. 2014 [2] and Wong et al. 2020 [22]). Moreover, we tested whether or not amino-acid and corresponding nucleotide sequences had evolved under stationary, (time-) reversible and homogeneous (SRH) conditions (see below). All descriptive statistics of the domain-based partitioned amino-acid supermatrices are given in Table 1 (main text) and in the Additional file 3: Fig. S35–S54. We used the masked supermatrix A (i.e. the supermatrix after removal of ambiguously aligned sections) as an input for the software MARE v. 0.1.2-rc [21]. We subsequently examined results of various strategies meant to optimize the amino-acid supermatrix:

(i) domain-based partitions and taxa with lowest information content excluded from supermatrix A (achieved by MARE with default settings): supermatrix B (a selected optimal subset of supermatrix A, SOS). Note that the species *Mengenilla moldrzyki* and *Micropterix calthella* were automatically dropped by MARE.

(ii) domain-based partitions with zero information content (IC=0) excluded from supermatrix A with the help of custom-made Perl scripts: supermatrix C.

(iii) taking supermatrix C as input and subsequently removing the species *Mengenilla moldrzyki* and *Micropterix calthella* (lowest pairwise sequence completeness scores C_a calculated with AliStat). After removal, still other representatives of the insect orders Strepsiptera and Lepidoptera (used as outgroups) were present: supermatrix D, 119 taxa.

(iv) supermatrix C used as an input for generating a SOS subset with MARE (default settings): supermatrix E.

We finally decided for supermatrix E at the amino-acid sequence level for all subsequent downstream analyses e.g., optimization of the partition-scheme, maximum likelihood (ML) tree inferences, and examine results for conflicting and confounding signal via four-cluster likelihood mapping (FcLM) and data permutations (see below).

In order to enable a direct comparison of the results of the multi-species coalescent approach with those of the concatenation approach, we additionally concatenated respective unmasked gene partitions at the amino-acid sequence level into two supermatrices: (1) a dataset comprising all gene partitions (supermatrix G), and (2) a reduced dataset in which we retained only those partitions that have a species coverage of more than or equal to 95 % of the total number of species in the dataset (supermatrix H). Moreover, we generated two reduced amino-acids supermatrices by keeping only these

sites in supermatrix E with at least 95 % or 90 % species coverage (supermatrix E-95 and supermatrix E-90 respectively, Additional file 1: Table S9).

1.6.2 Concatenation of nucleotide sequence datasets

We generated a nucleotide supermatrix corresponding to supermatrix D. This supermatrix was checked for violations of the assumption of evolution under SRH conditions with the application of Bowker's test (implemented in the software SymTest, see below), and subsequently 1st and 3rd codon were removed before final analyses (supermatrix D-nt, Additional file 1: Table S14).

We sought to enable a direct comparison of the results of the multi-species coalescent approach with the concatenation approach at the nucleotide sequence level, as well as with the results of the gene partitioned analyses at the amino-acid sequence level. For this purpose, we additionally generated two nucleotide sequence supermatrices according to gene-partitions (codon-based nucleotide sequence datasets with all three codon positions included): (1) a concatenated dataset comprising all gene partitions (supermatrix G-nt, Additional file 1: Table S14), and (2) a concatenated dataset with more than or equal to 95% species coverage for each gene partition (supermatrix H-nt, Additional file 1: Table S14).

1.6.3 Exploration of patterns of missing data and calculation of completeness scores for supermatrices

We calculated completeness summary statistics, i.e. (i) the overall completeness scores, C_a scores (based on sites), (ii) the completeness scores for sequence pairs (C_{ij}) with corresponding heatmaps using the software AliStat v. 1.6 (current version at

https://github.com/thomaskf/AliStat) of each of the amino-acid supermatrices A, B, C, D, E and F as well as for the decisive and RCFV-corrected versions of supermatrix E (command used: ./AliStat supermatrix.fas 2 -t4 -r both -i 2) [22]. This also allowed us to identify taxa with very low pairwise completeness scores. Low pairwise completeness scores calculated by AliStat can be due to missing data and/or ambiguous characters between the sequences of respective species, because ambiguous characters and gaps are treated in the same way. The inferred heatmaps help to identify patterns of non-random data coverage that might affect tree inferences. Similar completeness score statistics were calculated for the corresponding nucleotide matrix of supermatrix D consisting only of second codon positions (supermatrix D-nt, Additional file 1: Table S14). We further explored any potential effects of missing data on the phylogenetic inferences of amino-acid sequence data by using four-cluster likelihood mapping approach (FcLM) [23] with data permutations (see below).

1.6.4 Tests for violations of SRH conditions

Most Markov models of molecular evolution assume that sequences have evolved under SRH conditions [24–26]. The software SymTest, v. 2.0.47 (current version at https://github.com/ottmi/symtest) was used to test whether the amino-acid or nucleotide sequences in the supermatrices had evolved under SRH conditions. The Bowker's matched-pairs test of symmetry [25, 27] was selected as the most appropriate test for checking evolution assuming SRH processes in each supermatrix separately. The window size for the pairwise comparisons of sequences was automatically adjusted to the alignment length of the concatenated supermatrices. Based on p-values for each pairwise comparison between all terminal taxa, respective heatmaps were generated automatically. P-values lower than the significance level of the test (0.05) indicate deviation from SRH

conditions for a pairwise sequence comparison.

At the amino-acid sequence level we generated heatmaps of pairwise deviations from SRH conditions for supermatrices A, B, C, D, E, F and for the decisive and RCFV-corrected versions of supermatrix E (Table 1 of the main text, Additional file 1: Table S9). These heatmaps are useful for visualizing the degree of deviation from SRH processes for each taxon pair in the analyzed amino-acid or nucleotide datasets. At the nucleotide sequence level, we applied the same symmetry test to check whether or not SRH conditions are violated in the corresponding nucleotide dataset of supermatrix D. Specifically, we performed three separate analyses for this supermatrix: analysis of only 1) the first, 2) only the second, or 3) only the third codon positions. Since the second codon positions showed remarkably less violations of SRH conditions than the first and third codon positions, we kept only the second codon positions (supermatrix D-nt) for subsequent downstream analyses with the concatenation approach (i.e. optimization of the partitioning scheme and ML phylogenetic inference). We further explored any potential effects of non-stationary processes across taxa on the phylogenetic inferences of amino-acid sequence data by using FcLM tests with data permutations (see below).

1.6.5 Search for optimal meta-partitions and substitution models

We used PartitionFinder v. 2.0.0-pre11 (https://github.com/brettc/partitionfinder/releases/tag/v2.0.0-pre11, last access 02-04-2020) [28] with the option --raxml [29] and the rcluster algorithm [30] (options: --rcluster-percent 100 --weights 1,1,0,1 --all-states --min-subset-size 100) to optimize the domain-based partitioning scheme of supermatrix E for downstream phylogenetic analyses. The --rcluster-max option was adjusted to 10,905 (three times the number of domain-based

partitions). We restricted model selection to the following set of amino-acid substitution models in our PartitionFinder analysis: LG+G, LG+G+F [31], WAG+G, WAG+G+F [32], DCMUT+G, DCMUT+G+F [33], JTT+G, JTT+G+F [34], BLOSUM62+G, BLOSUM62+G+F [35]. Among site rate heterogeneity (ASRH) was modeled with a discrete GAMMA distribution with four rate categories [36] with or without empirical state frequencies (+G and +G+F). We additionally included the amino-acid model LG4X which is a mixture model using 4 free rate categories each associated to a different substitution matrix [37]. The selection of the best fitting substitution models was based on the AICc criterion [38, 39]. The branch-lengths option was set to 'linked' to relax the assumption of equal rates but assuming proportional branch lengths among meta-partitions. We conducted two analyses in PartitionFinder: (1) one by using a maximum likelihood tree as a starting tree (option: ml-tree) and (2) one without starting from a maximum likelihood tree (i.e. a maximum parsimony starting tree is used when the option –raxml is specified and the option ml-tree is omitted). We selected the partitioning scheme with the best AICc score between the two analyses for phylogenetic inference.

At the nucleotide sequence level, we used the same version of PartitionFinder to estimate the best partitioning-scheme for analyzing supermatrix D-nt (i.e. composed solely of second codon positions) by restricting the substitution models to: GTR+G and GTR+G+F. For clustering nucleotide sites into meta-partitions, we applied the *k*-means approach following the rationale described by Frandsen et al. (2015) [40]. We also performed two independent analyses with PartitionFinder for this nucleotide dataset: (1) with a maximum likelihood starting tree and (2) without a maximum likelihood starting tree (i.e. a maximum parsimony starting tree was used instead). For both analyses we used the options: --all-states --min-subset-size 100, and set the branch-lengths option to 'linked' in the

configuration file. The best partitioning scheme was selected according to the AICc criterion. We used custom made Perl scripts to physically resort supermatrix D-nt in a way, that nucleotide positions belonging to the same clusters (meta-partitions) were grouped next to each other within the matrix. For the ML tree inference, we recalculated the best model for each meta-partition in the resorted nucleotide supermatrix using IQ-TREE v. 1.3.13 and the AICc, this time allowing the following combinations of the GTR matrix and ASRH: GTR, GTR+G, GTR+I and GTR+G+I.

We did not perform any optimization of the partitioning scheme for the datasets that were partitioned according to genes in order to have a direct comparison of their results to those of the coalescent approach with ASTRAL III v. 5.6.1 [41]. Model selection for each gene partition in these supermatrices was performed in IQ-TREE v. 1.6.6a and by testing the same sets of amino-acid and nucleotide substitution models (amino-acid substitution matrices: DCMUT, JTTDCMUT, JTT, LG, LG4X, LG4M [37], WAG; nucleotide substitution matrices: GTR [42], JC [43], F81 [44], K80 [45], HKY [46], TN [47]) that were used to infer the gene trees for the coalescent approach, with the BIC criterion [48]. For all analyses based on the gene-partitions (concatenation-based and coalescent) the best fitting models for each gene were estimated directly in IQ-TREE (v. 1.6.3 when inferring the best fitting models to calculate the individual gene trees, and v. 1.6.6a when inferring the best fitting models for the genes within the supermatrices) by also considering the recently described freerate options for modelling ASRH (i.e. +RX and the option -gmedian) [49].

1.7 Phylogenetic tree reconstructions using the maximum likelihood criterion

Maximum likelihood phylogenetic analysis based on the best scheme and the selected models of supermatrix E was conducted by specifying an edge-linked (options: -spp)

partition model [50], and by starting from random trees (option: -t RANDOM and --numstop 300) instead of default parsimony trees to escape putative local optima in the parameter space during tree searches. After checking for the number of unique tree topologies with Unique Tree v. 1.9 (kindly provided by Thomas Wong) we selected the ML tree with the best log-likelihood score among all 100 independent tree searches as the globally best ML tree and mapped non-parametric bootstrap support values onto this tree. We checked for convergence of the bootstrap values using the bootstop criterion in RaxML v. 8.2 [29] by doing ten independent tests with different random seeds (settings: -I autoMRE -B 0.03 -m PROTGAMMALG –bootstop-perms=1000).

It has been shown that modeling site-heterogeneous profiles of amino-acid substitutions can improve the fit of the models to empirical data [51]. We therefore performed one additional tree search on supermatrix E with the Posterior Mean Site Frequency profile (PMSF) mixture model implemented in IQ-TREE v. 1.5.5 [52], used here as a rapid proxy to the empirical ML-based CAT-like mixture model with 60 profile categories (C60, see [53]). We used the transition rates of the JTT substitution matrix (option: -m JTT+C60+G+F) and the best ML tree that resulted from the partitioned analysis of supermatrix E (see above) as a guide tree for this analysis. The JTT substitution matrix was selected as it was the most frequently selected single-matrix model among the meta-partitions of supermatrix E. To minimize confounding effects of non-random data coverage on phylogenetic reconstructions that were based on the site-heterogeneous models, we finally generated two reduced versions of supermatrix E using custom-made Perl scripts by keeping only those sites with i) at least 90 % species coverage (207,582 amino-acid alignment sites) and ii) at least 95% species coverage (110,708 amino-acid alignment sites). For each of these reduced supermatrices, we conducted two additional tree

searches with the PMSF model, by specifying 60 mixture categories (C60): one with the substitution model JTT (-m JTT+C60+G+F) and one specifying LG (-m LG+C60+G+F). For the analyses of these reduced supermatrices we employed IQ-TREE v. 1.5.5, using the same guide tree as the ones used for analyzing supermatrix E.

We analyzed the decisive and RCFV-corrected versions of the amino-acid supermatrix E (supermatrix E-Decisive and supermatrix E-RCFV corrected respectively , Additional file 1: Table S9) with the use of a linked partition model and by conducting five additional ML searches for each of these two matrices, using IQ-TREE v. 1.6.6, starting from random trees. Branch lengths were kept linked in all analyses (-spp). We used the same version of IQ-TREE for calculating 1,000 ultrafast bootstrap (UFB) [54] replicates (options: -bb 1000 -bnni -wbtl) and 10,000 SH-aLRT replicates [55] (option: -alrt 10000). SH-aLRT support was mapped onto the best ML trees that resulted from the analyses of these two different versions of supermatrix E (Additional file 3: Fig. S6, S8). Because the phylogenetic trees that resulted from the independent tree searches when calculating UFB support were topologically different than the best ML trees for both matrices, UFB support was depicted on these additional trees instead of mapping them on the best ML trees (Additional file 3: Fig. S7, S9).

Maximum likelihood phylogenetic analyses of the nucleotide supermatrix D-nt were conducted in the same fashion with the analyses of supermatrix E and by using the previously selected most fitting substitution models for each meta-partition (i.e. 100 independent searches, options: -t RANDOM, -spp, -gmedian). We again checked for convergence of the bootstrap values using the bootstop check with the same version of RaxML and the same parameters that were used for analyzing the amino-acid supermatrix

E.

Phylogenetic analyses of the gene-partitioned supermatrices (all codon positions included at the nucleotide sequence level, supermatrices G, G-nt, H, H-nt, Additional file 1: Tables S9, S14) were done with IQ-TREE (v. 1.6.6 or later) again with the edge-linked partition model (options: -spp -gmedian) and with the previously inferred optimal substitution models. For the gene-partitioned amino-acid and nucleotide sequence datasets, with all 3,983 genes included, we inferred ten ML trees (five with a parsimony starting tree and five with a random starting tree). Due to computational limitations we only performed five ML searches for the datasets with 95% species coverage (again with the edge-linked model and starting from parsimony trees). Branch support for each of the analyses of the four gene-partitioned datasets (two at the amino-acid and two at the nucleotide sequence level) was calculated based on 1,000 UFB replicates and 10,000 SH-aLRT replicates. SH-aLRT support was mapped onto the best ML tree that resulted from the analyses of these four different supermatrices (Additional file 3: Fig. S19, S21, S27, S29). Because the phylogenetic trees that resulted from the independent runs when calculating UFB support were topologically different than the best ML trees for all four matrices, UFB support was depicted on these additional trees instead (Additional file 3: Fig. S18, S20, S26, S28).

1.8 Rogue-taxon analysis

We tested for the presence of rogue taxa in our dataset with RogueNaRok v. 1.0 [56]. The rogue taxon analysis was performed separately for: (1) the 100 bootstrap trees that resulted from the analyses of the amino-acid supermatrix E and (2) the 100 bootstrap trees that resulted from the analyses of the nucleotide supermatrix D-nt. In both analyses we provided the respective best ML tree as input for RogueNaRok.

1.9 Assessing phylogenetic signal with four-cluster likelihood mapping (FcLM) and sequence data permutations

Classical measures of robustness in phylogenetics such as non-parametric bootstrapping, have been introduced to assess robustness of tree reconstructions against statistical uncertainty of the results obtained for the dataset and a specific phylogenetic method. It is becoming increasingly clear, however, that resampling methods like non-parametric bootstrapping may very likely deliver inflated branch support values [2, 57]. A combination of FcLM methods with sequence data permutations can be applied to dissect phylogenetic from non-phylogenetic signal (i.e. confounding signal) for a set of predefined phylogenetic hypotheses [2, 58–60]. We have applied this approach to dissect phylogenetic from non-phylogenetic signal for nine hypotheses concerning the backbone tree of Neuropterida (Additional file 1: Table S2).

For each of the nine tested hypotheses, we first discarded uninformative meta-partitions with respect to particular group definitions (Additional file 1: Table S2). With these data, we performed the FcLM analyses by applying previously estimated substitution models (edge-linked models, -spp) with IQ-TREE (v. 1.4.4 or later). Additionally to this FcLM analyses applied on the original data, we permuted the original supermatrix E in three different ways following the approach described by Misof et al. (2014) [2]. Permutation scheme I eliminates phylogenetic signal, but leaves inhomogeneous amino-acid composition among sequences and any non-random distribution of data coverage untouched. Permutation scheme II eliminates phylogenetic signal and inhomogeneous amino-acid composition but leaves non-random distribution of data coverage untouched. Permutation scheme II was generated with the application of the amino-acid frequencies of the LG substitution model [1]. Permutation scheme III additionally randomizes data coverage within all meta-

partitions. The expectation is that any potentially genuine or confounding signal should completely vanish after applying permutation scheme III. For the analysis of the permuted supermatrices we used the option -q for the partition model in IQ-TREE. By comparing the FcLM results of the original data with the results of the permuted data we explored the relative contribution of confounding factors on our phylogenetic reconstructions (Additional file 1: Table S2).

1.9.1 Hypothesis 1: Megaloptera and Neuroptera are sister groups (Node: 167)

Our best ML tree implies that Megaloptera is the sister group of Neuroptera. If bootstrap or SH-aLRT support is inflated, FcLM on the original sequence data and on the permuted sequence data should indicate alternatives.

Group definitions: (a) Raphidioptera, (b) Megaloptera, (c) Neuroptera, (d) Outgroups

Note: We defined all outgroup species as the outgroup for the quartet analyses.

1.9.2 Hypothesis 2: The family Coniopterygidae is not the sister group of Sisyridae (Node: 179)

The reconstructed ML tree implies that Coniopterygidae is the sister group of all other Neuroptera. Morphological data is in conflict with this result. It has been proposed that Coniopterygidae and Sisyridae are sister groups [61, 62]. If there is signal for a sister group relationship of Coniopterygidae and Sisyridae, FcLM tests should show some signal for this phylogenetic arrangement.

Group definitions: (a) Coniopterygidae, (b) Osmylidae, (c) Sisyridae, (d) Nevrorthidae

Note: We defined Coniopterygidae as outgroup for the quartet analyses. Results of this analysis should be taken with caution due to the low number of analyzed quartets.

1.9.3 Hypothesis 3: The families Nevrorthidae and Sisyridae are sister groups (Node: 181)

The reconstructed ML tree infers Nevrorthidae and Sisyridae as sister groups. Morphological data is in strong conflict with this result. If there is only a weak signal for this sister group relationship, FcLM analyses should confirm this.

Group definitions: (a) Dilaridae + remaining Neuroptera (without Coniopterygidae), (b) Osmylidae, (c) Sisyridae, (d) Nevrorthidae

Note: We defined Dilaridae + remaining Neuroptera (without Coniopterygidae) as outgroup for the quartet analyses.

1.9.4 Hypothesis 4: The family Osmylidae is not sister to Chrysopidae (Node: 180)

The reconstructed ML tree implies Osmylidae as sister to a clade comprising Nevrorthidae + Sisyridae. Morphological data is in conflict with this result. Analyses of morphological data have suggested a sister group relationship of Osmylidae and Chrysopidae [62]. If true, we would expect to see some genuine phylogenetic signal supporting Chrysopidae + Osmylidae in the results of the FcLM analyses.

Group definitions A: (a) Osmylidae, (b) Chrysopidae + Mantispidae + Berothidae, (c) Dilaridae, (d) Nevrorthidae + Sisyridae

Group definitions B: (a) Osmylidae, (b) Chrysopidae, (c) Dilaridae, (d) Nevrorthidae + Sisyridae

Note: We defined Dilaridae as outgroup for the interpretation of the results of the quartet analyses. Results of this analysis should be taken with caution due to the low number of analyzed quartets.

1.9.5 Hypothesis 5: The family Nevrorthidae is not the sister group of all other Neuroptera (Node: 179)

Our best ML tree implies that Coniopterygidae is the sister group to all remaining neuropteran families. Morphological data is in conflict with this result. It has been hypothesized that Nevrorthidae could be the sister group of all remaining Neuroptera [62]. In this case FcLM tests should show some signal for (Nevrorthidae, Neuroptera partim).

Group definitions: (a) Megaloptera, (b) Coniopterygidae, (c) remaining Neuroptera without Osmylidae and Sisyridae, (d) Nevrorthidae

Note: We defined Megaloptera as outgroup for the interpretation of the results of the quartet analyses.

1.9.6 Hypothesis 6: The family Hemerobiidae is the sister group of Ithonidae + Myrmeleontiformia (Node 204)

Our best ML tree implies Chrysopidae and Mantispoidea are sister groups. Morphological data is in conflict with this hypothesis and suggests a sister group relationship of Chrysopidae and Hemerobiidae [63]. If the result of tree reconstructions is an artifact then the FcLM analyses should indicate alternative phylogenetic hypotheses.

Group definitions A: (a) Chrysopidae, (b) Mantispidae + Berothidae, (c) Hemerobiidae, (d) Myrmeleontidae + Ithonidae + Nymphidae + Ascalaphidae + Nemopteridae

Group definitions B: (a) Chrysopidae + Mantispidae + Berothidae, (b) Hemerobiidae, (c) Dilaridae, (d) Myrmeleontidae + Ithonidae + Nymphidae + Nemopteridae+ Ascalaphidae + Nemopteridae

Note: We defined Chrysopidae as outgroup in quartet analysis A and Dilaridae as outgroup in quartet analysis B.

1.9.7 Hypothesis 7: Myrmeleontiformia are monophyletic and form the sister group of Ithonidae (Node: 220)

Our best ML tree implies that the four included families of Myrmeleontiformia (i.e. Nymphidae, Nemopteridae, Ascalaphidae and Myrmeleontidae) constitute a monophylum. This is in conflict with recent phylogenomic investigations that suggest Nymphidae as the sister group of Ithonidae [64, 65]. If the topology suggested by the analysis of supermatrix E is an artifact, then quartets should indicate support for alternative hypotheses.

Group definitions: (a) Dilaridae + Mantispidae + Berothidae + Chrysopidae + Hemerobiidae, (b) Nemopteridae + Ascalaphidae + Myrmeleontidae, (c) Nymphidae, (d) Ithonidae

Note: We defined Dilaridae + Mantispidae + Berothidae + Chrysopidae + Hemerobiidae as outgroup for the interpretation of the results of the quartet analyses.

1.9.8 Hypothesis 8: Osmyloidea are monophyletic (Node: 180)

Our best ML tree implies that the families Nevrorthidae, and Osmylidae constitute a monophyletic group (i.e. Osmyloidea). If the topology suggested by the analysis of supermatrix E is an artifact, then quartets should indicate support for alternative hypotheses.

Group definitions: (a) Osmylidae, (b) Nevrorthidae + Sisyridae (c) Coniopterygidae + Megaloptera + Raphidioptera, (d) Dilaridae + Chrysopidae + Mantispoidea + Hemerobiidae

Note: We defined Coniopterygidae + Megaloptera + Raphidioptera as outgroup in quartet analyses.

1.9.9 Hypothesis 9: The family Chrysopidae is sister to Mantispoidea (Node: 185)

Our best ML tree implies that the family Chrysopidae is the sister group to a clade of Mantispidae + Berothidae. However, there is no morphological support for this phylogenetic relationship. If the topology suggested by the analysis of supermatrix E is an artifact, then quartets should indicate support for alternative hypotheses.

Group definitions: (a) Chrysopidae, (b) Mantispidae + Berothidae (c) Hemerobiidae + Ithonidae + Myrmeleontiformia (d) Dilaridae + Osmylidae + Sisyridae + Nevrorthidae + Coniopterygidae

Note: We defined Dilaridae + Osmylidae + Sisyridae + Nevrorthidae + Coniopterygidae as outgroup in quartet analyses.

1.10 Divergence times estimation

We used 129 meta-partitions of the decisive amino-acid supermatrix (i.e. supermatrix E-Decisive, Additional file 1: Table S9) to estimate the divergence times of the major lineages of Neuropterida. Specifically, we conservatively removed 80 out of the 209 meta-partitions before estimating divergence times which contained identical (or nearly identical) amino-acid sequences for a few terminal taxa. Those closely related sequences could cause problems in the estimation of the Hessian matrices in the CODEML analyses. In addition, in the selected meta-partitions all species had to be present. The estimation of the Hessian matrices for the remaining 129 meta-partitions was performed under the selected substitution models and by using four rate categories for the gamma distribution of site rates (options: ncatG = 4, fix_alpha = 0, alpha = 0.5, Small_Diff = 0.1e-6). Fossil calibrations were provided as soft minimum bounds (Additional file 1: Table S10) in the form of truncated Cauchy distributions with default parameters (option: L' ') [66]. The time unit in MCMCTREE was set to the default value of 100 million years. The samples of

parameter values that resulted from the analyses of the individual meta-partitions were combined and further analyzed as a single MCMC chain using a custom made summarizer script written in C++ (available from: https://github.com/xflouris/summarizer). We used custom made Python scripts and the library matplotlib to plot: (1) the posterior mean node time estimates from run 1 against the posterior mean node time estimates from run 2 (i.e. convergence plot), 2) the posterior mean node time estimates from run 1 against the 95% highest posterior density confidence interval width (95% HPD-CI width) for each node from run 1. Additionally, in order to verify that the two independent summarized MCMC chains (i.e. from run 1 and run 2) have converged on similar posterior node time estimates, we used the two summarized MCMC files from run 1 and from run 2 to plot median posterior node time estimates from the two independent runs on the same plot with R v. 3.4.3 (Additional file 3: Fig. S31). The node number IDs of the overall best ML tree that was used for estimation of divergence times (Fig. 1) are provided in Additional file 3: Fig. S55.

We performed two additional molecular dating analyses (two independent MCMC chains) for each of the 129 meta-partitions after excluding the fossil *Elmothone martynovae* [67] from the calibrations, because the exact affinity of *E. martynovae* (and of Permithonidae in general) to Neuroptera is dubious [68–70]. We again combined the resulting MCMC samples from all meta-partitions into one summarized MCMC file and calculated posterior mean and median node estimates as well as confidence intervals. The purpose of these additional analyses was to evaluate whether posterior node time estimates were severely affected the initial placement of the fossil *E. martynovae*.

1.11 Fossils calibrations used for divergence time estimates

1.11.1 Aparaglossata

Westphalomerope maryvonneae Nel et al. 2007 [71] (see Wolfe et al. 2016 [72]).

Specimen: see Wolfe et al. (2016).

Justification for phylogenetic placement**:** see Wolfe et al. 2016.

Age: see Wolfe et al. (2016).

1.11.2 Coleoptera

Triadogyrus sternalis Ponomarenko, 1977 [73] (see Wolfe et al. 2016 [72]).

Specimen: see Wolfe et al. (2016) [72].

Justification for phylogenetic placement: see Wolfe et al. (2016) [72].

Age: see Wolfe et al. (2016).

1.11.3 Mecopterida

Permopanorpa inaequalis Tillyard, 1926 [74] (see Wolfe et al. 2016 [72]).

Specimen**:** see Wolfe et al. 2016.

Justification for phylogenetic placement**:** see Wolfe et al. 2016.

Age: see Wolfe et al. 2016.

1.11.4 Megaloptera

Dobbertinia reticulata Handlirsch, 1920 [75]

Jurassic fossil in family Sialidae – typical venation from the family.

Specimen: Holotype: No. 123/68, isolated wing, housed in Institut für Geologische Wissenschaften of the Ernst-Moritz-Arndt-Universität Griefswald.

Justification for phylogenetic placement: It was used in phylogeny of Liu et al. (2014) [60].

Age: The fossil is from Dobbertin, Germany. From ammonite biostratigraphy (associated ammonites of *Eleganticeras elegantulum*, *Lobolytoceras siemensi* and *Harpoceras capellatum*) the locality has been correlated as being in the lower part of the *falciferum* zone, which is early Toarcian in age [77]. The boreal *falciferum* zone is equivalent to the *Harpoceras serpentinum* zone [78]. This zone is known to be succeeded by the *Hildoceras bifrons* zone, the base of which is dated as 180.36Mya ± 0.7Mya [72, 79].

1.11.5 Stem Neuroptera

Elmothone martynovae Carpenter, 1976 [67]

Oldest member of Permithonidae from Permian of Elmo, Kansas. Permithonidae considered sister to rest of Neuroptera [80]; however monophyly of Permithonidae is inconclusive, with the placement of other permithonids being obscure [68].

Specimen: Holotype, MCZ 5585, isolated forewing. Housed in Museum of Comparative Zoology, Harvard, USA.

Justification for phylogenetic placement: Used as stem group Neuroptera to calibrate age of Neuropterida in the study by Wang et al. (2016) [69]. Also used as fossil calibration for Neuropterida in Wolfe et al. (2016) [72]. Here we used it as minimum for the Megaloptera + Neuroptera split (i.e. stem Neuroptera) and performed one additional analysis after excluding it from the calibrations to check whether it affects node time estimates.

Age: Permian, Age given in Prokop et al. (2015). Cisuralian/Artinskian. Age given in Wang et al. (2016) [69] Artinskian – Kungurian (290.1±0.26 - 272.3±0.5). Wang et al. (2016) [69] used minimum age of 271.8 Mya, following Wolfe et al. (2016) [72].

1.11.6 Coniopterygidae - Crown Coniopteryginae

The earliest definitive members of the family are from the Lower Cretaceous amber of

Lebanon.

Libanosemidalis hammanaensis Azar et al. 2000 [81]

Specimen: 326A, complete male, deposited in the Museum National d'Histoire Naturelle, Paris.

Justification for phylogenetic placement: Placed within extant subfamily Coniopteryginae based on: forewing with only one r-m crossvein, vein M without two long stiff setae, no plicatyre on abdominal sternites. It shares with Aleuropteryginae: the hind wing with vein Rs branching from R1 very near the wing base. It is possible that the characters are plesiomorphic – which led to a tentative placement within Coniopteryginae (pending a phylogenetic analysis). Winterton et al. (2018) [64] used this fossil as a crown representative of Coniopteryginae to calibrate their proposed phylogeny of Neuropterida.

Age: Lebanese stratigraphy has recently been revised [82]. The upper boundary of the early Barremian is proposed to be the first appearance of the ammonite *Ancyloceras vandenheckii* [79]. Cyclostratigraphy dates the *A. vandenheckii* Zone beginning at 129.41 Mya [79], providing a minimum age for Lebanese amber fossils.

1.11.7 Nevrorthidae

Cretarophalis patrickmuelleri Wichard 2017 [83]

Specimen: Holotype: SMNS BU296, complete insect in amber, housed in the collection of the Staatliches Museum für Naturkunde Stuttgart.

Complete insect in Burmese amber, showing many of the diagnostic characters of family. Also an unamed larvae of family also recorded from the amber [83]. Other Nevrorthidae are known from the Eocene (Rovno amber: [84], and Baltic amber: [85].

Justification for phylogenetic placement: It has not been considered in any phylogenetic analysis, as it was just recently described. Body and wing characters are characteristic of

the family. The larva is also characteristic of the family.

Age: The fossil is from Burmese amber, the age of the deposit is 98.8 Mya (Albian-Cenomanian) based on U-Pb dating of zircons from the volcaniclastic matrix of the amber [86].

1.11.8 Stem Chrysopidae

Mesypochrysa c.f. *intermedia* Panfilov, 1980 [87]

Specimen: NIGP161886, almost complete insect (absent head), housed in Nanjing Institute of Geology and Palaeontology, China.

The fossil record of Chrysopidae stretches back to the Jurassic with the oldest representatives being from Daohugou, China and Karatau, Kazakhstan. *Mesypochrysa sinica* and *M.* c.f. *intermedia* represent the earliest records of true chrysopids [88]. Mesypochrysa is placed within the subfamily Limaiinae, which contains the majority of Mesozoic chrysopids [88]. The subfamily Limaiinae was raised to family level, Limaiidae, by Nel et al. 2005 [89], however subsequent authors have treated it as a subfamily of Chrysopidae [88, 90–92].

Justification for phylogenetic placement: The genus *Mesypochrysa* was included in the phylogeny described by Nel et al. 2005 [89]. It was placed within Limaiinae (Limaiidae sensu Nel et al. 2005 [89]). The genus has also been used to calibrate the phylogenies of Winterton et al. (2010) [93], *Mesypochrysa miniscula* in [94], and in Dai et al. 2016 [92], *Mesypochrysa minuta* in Jepson et al. 2012 [95]. *Mesypochrysa c.f. intermedia* Panfilov, 1980 is the oldest most complete specimen of *Mesypochrysa*. Winterton et al. (2018) [64] used *Mesypochrysa* c.f. *intermedia* as a stem representative of Chrysopidae in their fossils calibrations.

Age: The specimen is from the Jiulongshan Formation, Daohugou, China. There has been

some controversy as to the age of the Daohugou Beds [96–98], with estimates ranging from an Aalenian age (Middle Jurassic) to a Lower Cretaceous age [99], with some studies converging on the age of Callovian-Oxfordian (Upper Jurassic; [98]). Radiometric dating (40Ar/39Ar and SHRIMP U-Pb) of underlying ignimbrites have yielded ages of 165Mya ± 2.5Mya and 158.7Mya ± 0.6Mya [96, 100–102]. The Daouhugou beds have been shown to correlate with sediments from China and Kazakhstan of Oxfordian age [98]. Wolfe et al. (2016) [72] used a minimum age of 158.1Mya.

1.11.9 Stem Hemerobiidae

Promegalomus anomalus Panfilov, 1980 [87]

Specimen: Holotype: PIN 2239/1695, a complete but poorly preserved forewing, housed in the Palaeontological Institute of the Russian Academy of Sciences.

This specimen is the oldest known hemerobiid, it is a poorly preserved, yet complete forewing from the Jurassic of Kazakhstan. Panfilov (1980) [87] originally placed the specimen in a monotypic family Promegalomidae, this was later synonymized with Hemerobiidae by Oswald (1993) [103]. The family placement within Hemerobiidae was later confirmed by a re-examination of the holotype by Makarkin et al. (2003) [104]. The hemerobiid affinity is based on the wing venation synapomorphy: presence of multiple oblique radial branches. Only one definite Cretaceous Hemerobiid is known *Cretomerobius disjunctus* Ponomarenko, 1992 [105] (Mongolia), with numerous specimens known from the "Tertiary". The absent apomorphies of the family are: presence of penciliform sensillae on the galea; and clypeus with paired dorsocentral and ventrolateral setae [103, 106].

Justification for phylogenetic placement: Few phylogenies of Hemerobiidae have been undertaken, and none have incorporated both fossil and extant specimens. Therefore,

Promegalomus anomalus has not been used in any phylogenetic analysis. Oswald (1993) [103] discussed the species placing it within the family Hemerobiidae based on the aforementioned synapomorphy, but did not use this or any other fossil in his cladistics analysis. Garzón-Orduña et al. (2016) [106] briefly discussed the specimen, accepting its placement within Hemerobiidae, while stating that it would give a minimum age of the family (Hemerobiidae), it does not give information on the subfamilial relationships, therefore not of use to their study. Instead they used Miocene and Eocene fossils of specimens in extant genera to constrain ages. Winterton et al. (2018) [64] used *Promegalomus anomalus* as a stem representative of Hemerobiidae in their fossils calibrations.

Age: The fossil is from the Karabastau Formation, Karatau, Kazakhstan. Spore and pollen data has given an age of Callovian to Kimmeridgian [107] or more recently possibly Oxfordian to Kimmeridgian [108]. No radiometric analysis has been undertaken.

1.11.10 Stem Ithonidae

Guithone bethouxi Zheng, Ren and Wang 2016 [109]

Specimen: Holotype: CNU-NEU-NN2015003P/C, a nearly completely preserved male specimen with clearly visible structures, and four overlapping, sub-complete wings, partially folded. *Guithone bethouxi* shows some distinctive ithonid traits, such as the head retracted under pronotum and the broadened base of forewing costal space.

Justification for phylogenetic placement: Not available. It was used as a calibration point for the crown Ithonidae by Winterton et al. (2018) [53].

Age: The specimen is from Jiulongshan Formation, Daohugou, China. There has been some controversy as to the age of the Daohugou Beds [96–98], with estimates ranging from an Aalenian age (Middle Jurassic) to a Lower Cretaceous age [99, 110], with some

studies converging on the age of Callovian-Oxfordian (Upper Jurassic; [98]. Radiometric dating (40Ar/39Ar and SHRIMP U-Pb) of underlying ignimbrites have yielded ages of 165Mya ± 2.5Mya and 158.7Mya ± 0.6Mya [96, 100–102]. The Daouhugou beds have been shown to correlate with sediments from China and Kazakhstan of Oxfordian age [98]. Wolfe et al. (2016) [72] used a minimum age of 158.1Mya.

1.11.11 Stem Nemopteridae

Roesleriana exotica Martins-Neto and Vulcano, 1989 [111]

Specimen: Holotype: GP/1T-1627, complete insect. It is housed in the Collection of Paleontologica de Invertebrados do Instituto de Geosciencias da Univeridade de Sao Paulo.

The fossil record of Nemopteridae is very sparse, two fossils from the genus *Marquietta* are known from North America, from the Eocene Florrissant, Colorado and the Oligocene of Montana. A defining character of the family is the elongated spoon or ribbon-like hind wings. This feature is observed in some Mesozoic specimens: *Roesleriana exotica* Martins-Neto and Vulcano, 1989 and *Cratonemopterix robusta* Martins-Neto and Vulcano, 1989 from the Crato Formation. *Cratonemopteryx* was placed within Nemopteridae and *Roesleriana* was placed within Roeslerianidae. Martins-Neto and Vulcano (1989) [111] stated that Roeslerianidae are without doubt plesiomorphic nemopterids and should therefore be considered a subfamily of Nemopteridae. Some species of Araripeneurinae also have narrow hind wings and may represent remote stem-nemopterids [112, 113]. The araripeneurines are also from the Crato Formation.

Justification for phylogenetic placement: Few phylogenies have been performed on the relationships of Nemopteridae, and none has considered fossil species. A phylogeny of South African Nemopterinae, used the fossils of *Marquietta* from North America for calibration. The major apomorphy of the group is the elongate thread or spoon shaped

hind wings, which are also present in *Roesleriana exotica.* In their phylogeny of Myrmeleontidae Michel et al. (2017) [112] used the date of the Crato Formation (*Roesleriana exotica* and *Cratonemopterix robusta)* to calibrate dates for the crown representatives of Nemopteridae.

Age: The specimen is from the Crato Formation, Brazil. The age of the Formation has been dated as being at the Aptian/Albian boundary, the date being obtained by palynomorph studies [114]. No more accurate dating has been performed on the deposit. The Upper boundary of the Aptian is 113Mya ± 0.4Mya.

1.11.12 Stem Myrmeleontidae

Choromyrmeleon othneius Ren and Guo, 1996 [94]

Specimen: Holotype: LB95013/4, poorly preserved almost complete insect, housed in the Geological Museum of China, Beijing.

Justification for phylogenetic placement: It was used as a calibration by Winterton et al. (2010) [93] for calibrating the age of the most recent commom ancestor of Ascalaphidae and Myrmeleontidae. It was also used by Michel et al. (2017) [112] as a calibration for Ascalaphidae + Myrmeleontidae, however the authors stated that this is an inconclusive placement. This was due to the possibility that this is not a myrmeleontid, because of venational differences (e.g., *Choromyrmeleon*: MP2 and CuA in a very distal position in hind wing with respect to forewing – this is subequal in extant taxa) and unpreserved important characters (e.g. whether MP2 is fused with CuA). The other Mesozoic potential myrmeleontid fossil, *Bittersdorfia*, is known from an incompletely described isolated wing, which again its place in the family is hard to confirm. Michel et al. (2017) [112] ran the analysis with and without *Choromyrmeleon*, and found that this caused no difference.

Age: The fossil is from the Yixian Formation, Jehol Group, China. Recent 40Ar/39Ar dates

of basaltic rocks at the base of the Yixian Formation have yielded ages of 129.7 Mya±0.5 My and for tuff layers at the base of the overlying Jiufotang Formation have given an age of 122.1Mya±0.3My [100]. Other age estimates have fallen within this range [115]. Wolfe et al. (2016) [72] used a conservative date of the younger estimate, giving a minimum age of 121.8 Ma.

1.12 Ancestral character state reconstructions of the larval habitats of Neuropterida

We used the stochastic character mapping method (SCM) [116, 117], as implemented in the R package phytools v. 0.6.99 [118] to reconstruct the ancestral states of the larval habits of Neuropterida. In order to conduct the SCM analyses, we coded all species in our dataset as either aquatic or terrestrial. We assumed based on the literature that the larval habitats remain constant within families and therefore in cases where the states of larval habitats are unknown, these states were extrapolated from other species of the same family (note: we considered the larvae of *Lepicerus* sp. as either aquatic or terrestrial in two separate analyses [119], see Additional file 3: Fig. S32–S34, Additional file 1: Table S20). Subsequently, we used the R package geiger v. 2.0.6.4 [120] to separately fit an equal-rates model (ER) and an all-rates-different model (ARD) to our data by using the ultrametric tree of Fig. 1. We selected the model with the lowest AICc score for downstream SCM analyses (Additional file 1: Table S20). We then simulated 10,000 stochastic character maps based on the topology and branch lengths of the best ML tree using the best fitted model of character evolution. Simulations of character histories were performed using a fixed value of the transition matrix. The results of the different character maps were summarized using ape v. 5.3 [121] by visualizing the posterior probabilities of states at the nodes as pie charts (Fig. 4). To test the sensitivity of our SCM analyses to the topology and branch lengths of the used tree, we repeated these analyses for the

phylogram in Fig. 2a (Additional file 3: Fig. S32–S34, Additional file 1: Table S20).

2. Supplementary results

2.1 Orthology assignment

In 87 out of the 96 analyzed transcript libraries of Neuropterida, we were able to assign transcripts to more than 3,000 COGs (Additional file 1: Table S1). The transcriptome with the lowest number of assigned orthologs was one of the two transcriptomes referring to the megalopteran species *Sialis lutaria.* This specific transcriptome was excluded for further downstream analyses. The transcriptome of the corydalid species *Protohermes xanthodes* yielded the best assignment success rate, with 3,777 assigned transcripts. Among the outgroup taxa the highest success rate yielded *Harpegnathos saltator* with 3,909 assigned transcripts. Detailed results of the orthology assignment step are given in Additional file 1: Table S1.

2.2 Protein domain identification

The protein domain identification pipeline resulted in the annotation of 8,741 PfamA domain data blocks and 1,764 PfamB domain data blocks within the 3,983 gene sequence alignments (COGs). The annotated PfamA domains clustered into 328 different protein domain clans. In total, we identified 13,144 independent void sections among the 3,983 COGs. The mean number of annotated PfamA domains per gene was 2.19, while the mean number of annotated PfamB domains per gene was 0.44. The cumulative alignment length of PfamA domains was 1,046,857 amino-acid alignment positions. The cumulative alignment length of PfamB domains was 174,653 amino-acid alignment positions.

2.3 Exploration of patterns of missing data and violations of SRH conditions

Mengenilla moldrzyki (Strepsiptera) and *Micropterix calthella* (Lepidoptera) had the lowest pairwise alignment completeness scores in the amino-acid supermatrix C (Additional file 3: Fig. S37). These two species were also automatically dropped in the the analysis with MARE, as a result of their low alignment completeness. The heatmaps of pairwise alignment completeness scores generated with AliStat do not display any obvious signs of non-random distributions of data coverage in any of the analyzed amino-acid supermatrices. Visual inspection of the AliStat heatmap of supermatrix D-nt (Additional file 3: Fig. S43) does not indicate obvious signs of non-random distribution of data coverage either.

The Bowker's tests of symmetry on the reduced versions of supermatrix E (decisive and RCFV-corrected) indicate a reduced level of deviation from global SRH conditions in these matrices. The pairwise p-values for the Bowker's test of symmetry were larger for the decisive matrix (median: 3.29e-013) than for the RCFV-corrected matrix (median: 9.33e-018) indicating that deviation from SRH conditions were significantly reduced in the decisive matrix (see also Additional file 3: Fig. S50, S51). All other generated amino-acid supermatrices show elevated levels of deviation from global SRH conditions (see Table 1 main Text and Additional file 3: Fig. S44–S49). When analyzing the corresponding nucleotide supermatrix of supermatrix D, the second codon positions showed the least deviation from SRH conditions (Additional file 3: Fig. S52–S54) by having the largest median and the largest mean pairwise p-values for the Bowker's test among taxa (median 1st: 0.000e+00, median 2nd: 3.028e-03, median 3rd: 0.000e+00, where 0.000e+00 indicates values close to zero).

2.4 Model selection and phylogenetic analyses of protein-domain-based partitioned amino-acid sequence data and phylogenetic analyses of second codon positions

The analyses of supermatrices E and D-nt with PartitionFinder resulted in calculated partitioning schemes with better AICc scores when a maximum-likelihood starting tree was used (AICc with option ml-tree: 64791636 for amino acids and 43149227 for second codon-positions, AICC without ml-tree option: 64806990 for amino acids and 43166061 for second codon positions). LG4X was the most frequently selected model at the amino-acid sequence level (684 meta-partitions). The analysis with PartitionFinder resulted in 1,825 meta-partitions in the calculated and optimized partitioning scheme of the amino-acid supermatrix E. The optimized partitioning scheme of supermatrix D-nt comprised 168 meta-partitions. The bootstop checks in RaxML converged mostly after 50 replicates suggesting that 100 bootstrap replicates are sufficient for the convergence of the bootstrap values for both datasets. Phylogenetic analyses of amino-acid and nucleotide datasets with the optimized partitioning schemes yielded 1 unique topology among all 100 tree searches for each of the these two separate datasets. These topologies resulted from the analyses of these two data types are highly congruent for the major clades of Neuropterida and only show slight topological differences at the very shallow nodes of the tree (see Fig. 1 of the main Text and Additional file 3: Fig. S1–S5). Additionally, the topologies that resulted from the reduced versions of supermatrix E (decisive and RCFV-corrected) are highly congruent to the above mentioned topologies for the Neuropterida backbone tree, except for a difference within the outgroup taxa (Coleoptera are paraphyletic with respect to Strepsiptera, Additional file 3: Fig. S6–S9). The analyses of the amino-acid sequence alignments with the site-heterogeneous models suggested topologies identical with the analyses of the above-mentioned partitioned datasets concerning the major phylogenetic splits in Neuropterida backbone tree (Additional file 3: Fig. S10–S14).

The rogue-taxon analyses of the amino-acid and the nucleotide sequence data did not reveal substantial contribution of rogue taxa in the phylogenetic results. Specifically, we identified the amino acid dataset to be rogue taxon-free; analyses of the nucleotide supermatrix D-nt flagged one species, *Turcoraphidia amara* as rogue taxon. The difference in the placement of this taxon refers to its placement within Raphidiidae and therefore does not affect the bootstrap values of the backbone tree of Neuropterida.

2.5 Summary coalescent and concatenation-based analyses of gene partitions

At the amino-acid sequence level the results of the concatenation-based analyses of the gene-partitions are highly congruent with those obtained from the analyses of the protein-domain-partitioned supermatrices. As in the analyses of all datasets of protein-domain partitions, the phylogenetic analyses of gene partitions supported a sister group relationship of Hemerobiidae to a clade of Ithonidae + monophyletic Myrmeleontiformia. The above-mentioned sister group relationship is recovered in every analysis of amino-acid sequence dataset analyzed irrespective of the method or the partitioning strategy that was used (Additional file 3: Fig. S1, S2, S6–S9, S10–S14, S15–S21). However, the Chrysopidae + Mantispoidea clade is disrupted in the concatenation-based analyses of genes at the amino-acid sequence level when genes were filtered for species coverage (Additional file 3: Fig. S20, S21). Overall, the topologies that resulted from the summary coalescent phylogenetic analyses of genes at the amino-acid sequence level are very similar to the topologies that resulted from the partitioned concatenated analyses of protein domains (or genes). The main difference between coalescent and concatenated analyses of amino-acid data is that the quartet support values are indicative of the conflict in the data, while the local posterior probabilities, non-parametric bootstrap, or Bootstrap by

transfer (TBE) support values are overall less conservative (Additional file 3: Fig. S1–S2, Additional file 1: Table S16–S19). An example of this pattern is the support for the clade Hemerobiidae + Myrmeleontiformia which is stable across analyses of amino-acid sequence data (Additional file 3: Fig. S1–S14, S18–S21). UFB, SH-aLRT, non-parametric bootstrap, TBE support, and local posterior probabilities are consistently high for a clade Hemerobiidae + Myrmeleontiformia (e.g. Fig. 1, Additional file 3: Fig. S1–S2, S6–S9), while quartet support for the given topology is weak (e.g. Fig. 2a, Additional file 1: Tables S16, S18). SH-aLRT and UFB support for the monophyly of the clade Mantispoidea + Chrysopidae is very low when analyzing the decisive amino-acid dataset (supermatrix E-decisive: 57 % and 66 % respectively, Additional file 3: Fig. S8, S9). UFB and SH-aLRT support is high for a clade of Mantispoidea + Chrysopidae in the analysis of the RCFV-corrected matrix (98 % and 91% respectively, Additional file 3: Fig. S6, S7).

Partitioned phylogenetic analyses of the second codon positions of the nucleotide sequence data suggested identical topologies with analyses of domain-partitioned amino-acid sequence data for the major phylogenetic relationships of Neuropterida with high bootstrap support (Additional file 3: Fig. S3–S5). When analyzing complete codon-based nucleotide sequence supermatrices partitioned according to genes, we considered datasets based all three codon positions. Overall, the phylogenetic relationships of Neuropterida (especially concerning the interrelationships of the clades: Hemerobiidae, Ithonidae + Myrmeleontiformia, Chrysopidae and Mantispoidea) are more unstable across analyses and datasets when analyzing gene partitions of nucleotide sequences with all three codon positions included (Additional file 3: Fig. S22–S29). The main difference between the analyses of codon-based nucleotide sequence data (both coalescent and concatenation-based) with previously the mentioned analyses of amino acid sequence

alignments or those of second codon positions is the placement of Chrysopidae (instead of Hemerobiidae) as sister to a clade of Ithonidae + paraphyletic Myrmeleontiformia (Additional file 3: Fig. S22–S29). The paraphyly of Myrmeleontiformia (except Psychopsidae for which no data were available) stems from a sister group relationship of Nymphidae to Ithonidae with high statistical UFB and SH-aLRT support (Additional file 3: S22–S29). The paraphyly of Myrmeleontiformia and the sister group relationship of Chrysopidae to a clade of Ithonidae + paraphyletic Myrmeleontiformia is recovered in the analyses of codon-based nucleotide sequence data irrespective of whether we applied a concatenation-based or a summary coalescent approach (Additional file 3: Fig. S22–S29).

In the analyses of codon-based nucleotide sequence data Osmylidae instead of Sisyridae is recovered as the sister group of Nevrorthidae. This relationship is supported by all coalescent analyses irrespective of the data type analyzed (Fig. 2a, Additional file 3: Fig. S15–S17, S22–S25). However, quartet support calculated with ASTRAL shows substantial conflict among gene trees for this particular phylogenetic question (Additional file 1: Tables S16–S19). The sister group relationship of Osmylidae and Nevrorthidae is not in agreement with the analyses of second-codon positions (supermatrix D-nt, Additional file 3: Fig. S3–S5) which suggest the same topologies as the concatenation-based analyses of amino-acids (Fig. 1 of the main text, Additional file 3: Fig. S1, S2).

Within the herewith inferred monophyletic Raphidioptera, Raphidiidae was inferred as monophyletic with high bootstrap support in all phylogenetic analyses. The nearctic genus *Agulla* was inferred as sister to all Palearctic Raphidiidae also with high bootstrap support Fig. 1, non-parametric bootstrap support: 100 %). Within the Palearctic Raphidiidae the genus *Mongoloraphidia* was inferred as sister to all other palearctic Raphidiidae. In

general, these results are stable across all concatenation-based analyses of amino-acid sequence alignments (Additional file 3: Fig. S1, S2, S6–S14, S18–S21), but non-parametric bootstrap support for the inter-relationships of the palearctic Raphidiidae is low (Fig. 1 of the main text). However, additional topological differences were observed between summary coalescent and concatenation-based analyses concerning the inferred phylogenetic relationships within the palearctic Raphidiidae. Specifically, the summary coalescent analyses did not support the placement of the palearctic genus *Mongoloraphidia* as sister to the remaining palearctic Raphidiidae (Additional file 3: Fig. S15–17, S22–S25). In general, all summary coalescent and concatenation-based analyses suggested a low level of molecular divergence among the species of palearctic Raphidiidae. Quartet support calculated with ASTRAL is also indicative of the extensive amounts of gene-tree conflict concerning the inter-relationships of the palearctic Raphidiidae (Additional file 3: Fig. S15–17, S22–S25).

2.6 FcLM branch support tests with and without data permutations

The phylogenetic relationships between the three orders Raphidioptera, Megaloptera and Neuroptera is robustly resolved based on the results of tree reconstructions and FcLM analyses. Megaloptera and Neuroptera are sister groups. Within Neuroptera, Coniopterygidae are sister to all other Neuroptera. Osmylidae, Sisyridae and Nevrorthidae form a monophyletic clade. Support for the monophyly of Osmyloidea is indirect from the results of FcLM under hypothesis 4a but also directly from the FcLM results under hypothesis 8. Dilaridae are the sister group to all remaining Neuroptera, excluding the previously mentioned families. Nymphidae, Nemopteridae, Ascalaphidae and Myrmeleontidae form a monophyletic clade. All these relationships mentioned above receive high bootstrap support and high support in the quartet tests based on the original

data and permutations (see also Additional file 1: Table S2).

The overall best ML tree (Fig. 1) displays a clade of Mantispoidea + Chrysopidae with high bootstrap and high SH-aLRT support (97 % and 98.8 % respectively) (node 185). This clade does not receive unequivocal support from the quartet analyses (Fig. 2a, Additional file 1: Table S2, Hypothesis 9). The tree also displays a clade of Hemerobiidae + (Ithonidae + [Nymphidae, Nemopteridae, Ascalaphidae and Myrmeleontidae]) (node 204). This clade receives strong bootstrap and SH-aLRT support in all analyses of amino-acid sequence data but shows unequivocal quartet support in the FcLM analyses and in the ASTRAL quartet analyses. It should therefore as well be regarded as only weakly supported. The unequivocal support for the above-mentioned nodes in the FcLM analyses is in agreement with the low quartet support received for these nodes from the coalescent-based approach (e.g. Fig. 2a). A sister group relationship of Sisyridae and Nevrorthidae (node 181) is not supported by the FcLM analyses, albeit the number of informative quartets for this particular question is extremely low to evaluate the robustness of this hypothesis (eight quartets). Quartet support as resulted from the analysis of the coalescent approach also shows weak support for the interrelationships of Sisyridae, Osmylidae, and Nevrorthidae (e.g. Additional file 1: Table S16, S18). With the use of different definitions of groups of taxa (Hypothesis 3, Additional file 1: Table S2) FcLM and ASTRAL quartet support show almost equal proportion of quartets supporting Osmylidae + (Nevrorthidae + Sisyridae) and Sisyridae + (Nevrorthidae + Osmylidae).

2.6.1 Hypothesis 1: Megaloptera and Neuroptera are sister taxa (Node: 167)

Quartet-Expectation: (Raphidioptera, Outgroups), (Megaloptera, Neuroptera)

Statistics: 276,480 quartets; 119 taxa, 1,825 partitions, no partitions dropped

In total ~86 % of the quartets favor (Raphidioptera, Outgroups), (Megaloptera, Neuroptera), 14 % (Raphidioptera, Megaloptera), (Outgroups, Neuroptera). Permutations indicate a confounding effect most likely coming from the distribution of missing data for (Raphidioptera, Megaloptera), (Outgroups, Neuroptera). This preference for (Raphidioptera, Megaloptera), (Outgroups, Neuroptera) vanishes in permutation III. Taken together this result gives strong confidence in (Raphidioptera, Outgroups), (Megaloptera, Neuroptera).

2.6.2 Hypothesis 2: Coniopterygidae are not the sister group of Sisyridae (Node: 179)

Quartet-Expectation: (Coniopterygidae, Osmylidae), (Sisyridae, Nevrorthidae)

Statistics: 8 quartets; 8 taxa, 1,733 partitions, 92 partitions dropped

In total 100 % of the quartets favor (Coniopterygidae, Sisyridae), (Osmylidae, Nevrorthidae), in contrast to the expectation. Permutations indicate potential confounding factors for the original result of the tree reconstructions (62.5 % of quartets). However, permutation III is very strongly biased and the number of quartets for this FcLM analysis is very low, therefore the results of this analysis should be taken with great caution.

2.6.3 Hypothesis 3: Nevrorthidae and Sisyridae are sister groups (Node: 181)

Quartet-Expectation: (Neuroptera partim., Osmylidae), (Sisyridae, Nevrorthidae)

Statistics: 112 quartets; 60 taxa; 1,759 partitions; 66 partitions dropped

In total 52 % of the quartets favor the expected relationship, 48 % the alternative (Neuroptera partim., Sisyridae), (Osmylidae, Nevrorthidae). Confounding factors favor (Neuroptera partim., Nevrorthidae), (Sisyridae, Osmylidae). Taken together this weakly supports the original hypothesis. Note that results must be taken with caution due to the

low number of analyzed quartets.

2.6.4 Hypothesis 4: The family Osmylidae is not the sister group of Chrysopidae (Node: 180)

Quartet-Expectation A: (Osmylidae, Nevrorthidae + Sisyridae), (Chrysopidae + Mantispoidea, Dilaridae)

Statistics: 60 quartets; 25 taxa; 1,752 partitions; 73 partitions dropped

In total 100 % of the quartets favor (Osmylidae, Nevrorthidae + Sisyridae), (Chrysopidae + Mantispoidea, Dilaridae). There are no strong confounding factors detectable.

Quartet-Expectation B: (Osmylidae, Nevrorthidae + Sisyridae), (Chrysopidae +Mantispoidea, Dilaridae)

Statistics: 45 quartets; 20 taxa; 1,751 partitions; 74 partitions dropped

In total 100 % of the quartets favor (Osmylidae, Nevrorthidae + Sisyridae), (Chrysopidae, Dilaridae). There are confounding factors favoring the original hypothesis in quartet expectation b. It should therefore accepted with caution.

2.6.5 Hypothesis 5: The family Nevrorthidae is not the sister group to all other Neuroptera (Node: 179)

Quartet-Expectation: (Megaloptera, Coniopterygidae), (Neuroptera partim, Nevrorthidae)

Statistics: 1,792 quartets; 69 taxa; 1,778 partitions; 47 partitions dropped

In total 94 % of the quartets favor (Megaloptera, Coniopterygidae), (Neuroptera partim, Nevrorthidae). There are confounding factors for (Megaloptera, Nevrorthidae). Taken together there is strong support for the original results.

2.6.6 Hypothesis 6: The family Hemerobiidae is the sister group of Ithonidae + Myrmeleontiformia (Node 204)

Quartet-Expectation A: (Chrysopidae, Mantispidae + Berothidae), (Hemerobiidae, Myrmeleontidae + Ithonidae + Nymphidae + Ascalaphidae + Nemopteridae)

Statistics: 22,800 quartets; 55 taxa; 1,824 partitions; 1 partitions dropped

In total 72.4 % of the quartets support (Chrysopidae, Mantispidae), (Hemerobiidae, Myrmeleontidae + Ithonidae + Nymphidae + Ascalaphidae + Nemopteridae + Dilaridae). There is confounding signal supporting this result, it should therefore be taken with caution (Additional file 1: Table S2).

Quartet-Expectation B: (Chrysopidae + Mantispidae + Berothidae, Dilaridae), (Hemerobiidae, Myrmeleontidae + Ithonidae + Ascalaphidae + Nemopteridae))

Statistics: 6,080 quartets; 57 taxa; 1,793 partitions; 32 partitions dropped

There is almost no support for any hypotheses in these quartet analyses. Also not for the original result derived from the tree reconstruction (Chrysopidae + Mantispidae + Berothidae, Dilaridae), (Hemerobiidae, Myrmeleontidae + Ithonidae + Nymphidae + Ascalaphidae + Nemopteridae).

2.6.7 Hypothesis 7: Myrmeleontiformia are monophyletic and sister to Ithonidae (Node: 220)

Quartet-Expectation: (Dilaridae + Mantispidae + Berothidae+ Chrysopidae + Hemerobiidae, Nymphidae), (Myrmeleontidae + Ascalaphidae + Nemopteridae, Ithonidae)

Statistics: 1,184 quartets; 57 taxa; 1,643 partitions; 182 partitions dropped

There is strong support for the ML tree reconstruction supporting monophyletic Myrmeleontiformia. Confounding signal in favor of this hypothesis is not detectable.

2.6.8 Hypothesis 8: Osmyloidea are monophyletic (Node: 180)

Quartet-Expectation: (Osmylidae, Nevrorthidae + Sisyridae), (Coniopterygidae + Megaloptera + Raphidioptera, Dilaridae + Chrysopidae + Mantispoidea + Hemerobiidae)

Statistics: 3,552 quartets; 73 taxa; 1779 partitions; 46 partitions dropped

There is strong support from quartet analyses in favor of the monophyly of Osmyloidea (99.70 % of quartets). There is no detectable confounding signal in favor of the original hypothesis.

2.6.9 Hypothesis 9: The family Chrysopidae is sister to Mantispoidea (Node: 185)

Quartet-Expectation: (Chrysopidae, Mantispidae + Berothidae), (Hemerobiidae + Ithonidae + Myrmeleontiformia, Dilaridae + Osmylidae + Sisyridae + Nevrorthidae + Coniopterygidae)

Statistics: 23,625 quartets; 64 taxa; 1824 partitions; 1 partition dropped

There is conflicting signal from quartets and no strong support for the original result of tree reconstructions (Additional file 1: Table S2). Taken together this weakly supports the original hypothesis.

2.7 Divergence times estimation

The divergence time estimates that resulted from the two independent combined analyses in MCMCTree resulted in very close estimates of mean and median ages for the major lineages of Neuropterida when using the full set of the twelve fossil calibration points (Additional file 1: Table S3–S4, Additional file 3: Fig. S30–S31). The median node age estimates of the meta-partitions from the two runs are almost identical as they overlap with each other in our beanplots of medians when comparing the two different runs (Additional file 3: Fig. S31). The additional analyses when exluding the fossil *Elmothone martynovae*

from the calibrations resulted very similar posterior median, mean and confidence interval values for the ages of the major nodes of Neuropterida, suggesting that the placement of *Elmothone martynovae* did not significantly affect the posterior node age estimates (Additional file 1: Table S12–S13). Overall, the posterior updated estimates of node ages suggested much younger ages than the prior marginal distributions for the shallow nodes and more narrow confidence intervals (Additional file 3: Fig. S56, Additional file 1: Tables S3–S4, S11). There exists a stronger correlation between the posterior and the prior mean ages for the deep nodes (as well as for the confidence interval width, Additional file 1: Tables S3–S4, S11, Additional file 3: Fig. S56) which most probably stems from the placement of a hard maximum bound at the root in all of our analyses. Nevertheless posterior confidence intervals for the ages overlap well with the prior confidence intervals (Additional file 1: Tables S3–S4) suggesting that there is no strong conflict between the prior marginal distributions and the posterior distribution of node ages.

Interestingly, the inferred mean and median divergence time estimates for the crown representatives of the clade Ascalaphidae + Myrmeleontidae are younger than the minimum age that was defined *a priori* for this clade. Since the two independent MCMC runs converged on similar age estimates for this node, the observed conflict is unlikely due to the lack of convergence. There are several possible reasons for this conflicting result, including the putative non-monophyly of Myrmeleontidae (see discussion of main text) and the conflict among meta-partitions for this relationship, the lack of the possibly most basal antlions in our dataset such as those in the tribe Stilbopterygini [112, 122], and node calibration or clock model misspecifications. Nevertheless, the 95 % posterior confidence intervals for the origin of the clade Ascalaphidae + Myrmeleontidae show some overlap with the minimum age defined *a priori* for this clade (Fig. 1, Additional file 1: Tables S3–

S4).

2.8 Ancestral character state reconstructions of the larval habitats of Neuropterida

On average there were 5.88 changes between states along the branches of each stochastic character map (analysis presented in Fig. 4). The results of the stochastic character mapping analyses suggest that ancestral Neuropterida most likely had terrestrial larvae (pp_aquatic = 0.0008 and pp_terrestrial = 0.9992 in analyses of Fig. 4, see also Additional file 2: Fig. S32–S34). In addition, the results suggest a primarily terrestrial larva in the last common ancestor of Neuroptera (pp_aquatic = 0.0008 and pp_terrestrial = 0.9992 in analyses of Fig. 4, see also Additional file 2: Fig. S32–S34). Furthermore, primarily terrestrial larvae in the last common ancestor of Megaloptera + Neuroptera is inferred as the most likely scenario given our data (pp_aquatic = 0.0034 and pp_terrestrial = 0.9966 in analyses of Fig. 4, see also Additional file 2: Fig. S32–S34). The larvae of Megaloptera were inferred as primarily aquatic (pp_aquatic = 0.7994 and pp_terrestrial = 0.2006 in analyses of Fig. 4, see also Additional file 2: Fig. S32–S34). These results are stable irrespective of whether the larva of *Lepicerus* sp. was coded as aquatic or terrestrial (Fig. 4, Additional file 2: Fig. S32–S34). Moreover, these results are strongly supported irrespective of the inferred relationships within Osmyloidea (Fig. 4, Additional file 3: Fig. S32–34). It has been pointed out that branch lenghts in coalescent units calculated with ASTRAL should be taken with caution [123]. Despite this, the different SCM analyses based on the coalescent-based trees produced similar results with the SCM analyses based on the branch lengths of ultrametric trees (i.e. from molecular dating analyses) concerning the common ancestor of Neuroptera and Neuropterida (Fig. 4, Additional file 3: Fig. S32–34).

3. Availability of data and materials

The datasets and additional information supporting the conclusions of this article are available in the Dryad digital repository, https://doi.org/10.5061/dryad.1jwstqjrs. The data deposited in Dryad include: 1) the ortholog set used as input for Orthograph, 2) all analyzed supermatrices, 3) datasets used for four-cluster likelihood mapping analyses, 4) meta-partitions used for estimation of divergence times, 5) species trees inferred with ASTRAL, 6) best phylogenetic tree that resulted from the analyses of supermatrix E, and 7) Gene trees used for summary coalescent analyses with ASTRAL.

References

1. Peters RS, Krogmann L, Mayer C, Donath A, Gunkel S, Meusemann K, et al. Evolutionary history of the Hymenoptera. Curr Biol. 2017;27:1013–8.

2. Misof B, Liu S, Meusemann K, Peters RS, Donath A, Mayer C, et al. Phylogenomics resolves the timing and pattern of insect evolution. Science. 2014;346:763–7.

3. Xie Y, Wu G, Tang J, Luo R, Patterson J, Liu S, et al. SOAPdenovo-Trans: *De novo* transcriptome assembly with short RNA-Seq reads. Bioinformatics. 2014;30:1660–6.

4. McKenna DD, Shin S, Ahrens D, Balke M, Beza-Beza C, Clarke DJ, et al. The evolution and genomic basis of beetle diversity. Proc Natl Acad Sci U S A. 2019;116:24729–37.

5. Giraldo-Calderón GI, Emrich SJ, MacCallum RM, Maslen G, Dialynas E, Topalis P, et al. VectorBase: an updated bioinformatics resource for invertebrate vectors and other organisms related with human diseases. Nucleic Acids Res. 2015;43 Database issue:D707–13.

6. Munoz-Torres MC, Reese JT, Childers CP, Bennett AK, Sundaram JP, Childs KL, et al. Hymenoptera Genome Database: integrated community resources for insect species of the order Hymenoptera. Nucleic Acids Res. 2011;39 Database issue:D658–62.

7. Waterhouse RM, Tegenfeldt F, Li J, Zdobnov EM, Kriventseva E V. OrthoDB: A hierarchical catalog of animal, fungal and bacterial orthologs. Nucleic Acids Res. 2013;41:1–8.

8. Nygaard S, Zhang G, Schiøtt M, Li C, Wurm Y, Hu H, et al. The genome of the leaf-cutting ant *Acromyrmex echinatior* suggests key adaptations to advanced social life and fungus farming. Genome Res. 2011;21:1339–48.

9. Richards S, Gibbs RA, Weinstock GM, Brown SJ, Denell R, Beeman RW, et al. The

genome of the model beetle and pest *Tribolium castaneum*. Nature. 2008;452:949–55.

10. Xia Q, Zhou Z, Lu C, Cheng D, Dai F, Li B, et al. A draft sequence for the genome of the domesticated silkworm (*Bombyx mori*). Science. 2004;306:1937–40.

11. Adams MD, Celniker SE, Holt RA, Evans CA, Gocayne JD, Amanatides PG, et al. The genome sequence of *Drosophila melanogaster*. Science. 2000;287:2185–95.

12. Tatusov RL, Koonin E V, Lipman DJ. A genomic perspective on protein families. Science. 1997;278:631–7.

13. Dos Santos G, Schroeder AJ, Goodman JL, Strelets VB, Crosby MA, Thurmond J, et al. FlyBase: introduction of the *Drosophila melanogaster* Release 6 reference genome assembly and large-scale migration of genome annotations. Nucleic Acids Res. 2015;43 Database issue:D690-7.

14. Wang J, Xia Q, He X, Dai M, Ruan J, Chen J, et al. SilkDB: A knowledgebase for silkworm biology and genomics. Nucleic Acids Res. 2005;33 Database issue:399–402.

15. Kim HS, Murphy T, Xia J, Caragea D, Park Y, Beeman RW, et al. BeetleBase in 2010: revisions to provide comprehensive genomic information for *Tribolium castaneum*. Nucleic Acids Res. 2010;38 Database issue:D437–42.

16. Petersen M, Meusemann K, Donath A, Dowling D, Liu S, Peters RS, et al. Orthograph: A versatile tool for mapping coding nucleotide sequences to clusters of orthologous genes. BMC Bioinformatics. 2017;18:111.

17. Suyama M, Torrents D, Bork P. PAL2NAL: Robust conversion of protein sequence alignments into the corresponding codon alignments. Nucleic Acids Res. 2006;34 suppl. 2:609–12.

18. Misof B, Misof K. A Monte Carlo approach successfully identifies randomness in multiple sequence alignments: a more objective means of data exclusion. Syst Biol. 2009;58:21–34.

19. Kück P, Meusemann K, Dambach J, Thormann B, von Reumont BM, Wägele JW, et al. Parametric and non-parametric masking of randomness in sequence alignments can be improved and leads to better resolved trees. Front Zool. 2010;7:10.

20. Finn RD, Bateman A, Clements J, Coggill P, Eberhardt RY, Eddy SR, et al. Pfam: The protein families database. Nucleic Acids Res. 2014;42:222–30.

21. Misof B, Meyer B, von Reumont BM, Kück P, Misof K, Meusemann K. Selecting informative subsets of sparse supermatrices increases the chance to find correct trees. BMC Bioinformatics. 2013;14:348.

22. Wong TKF, Kalyaanamoorthy S, Meusemann K, Yeates DK, Misof B, Jermiin LS. A minimum reporting standard for multiple sequence alignments. NAR Genomics Bioinforma. 2020;2.

23. Strimmer K, von Haeseler A. Likelihood-mapping: a simple method to visualize phylogenetic content of a sequence alignment. Proc Natl Acad Sci U S A. 1997;94:6815–9.

24. Jermiin L, Ho SY, Ababneh F, Robinson J, Larkum AW. The biasing effect of compositional heterogeneity on phylogenetic estimates may be underestimated. Syst Biol. 2004;53:638–43.

25. Ababneh F, Jermiin LS, Ma C, Robinson J. Matched-pairs tests of homogeneity with applications to homologous nucleotide sequences. Bioinformatics. 2006;22:1225–31.

26. Jayaswal V, Jermiin LS, Robinson J. Estimation of phylogeny using a general Markov model. Evol Bioinform Online. 2005;1:62–80.

27. Bowker AH. A test for symmetry in contingency tables. J Am Stat Assoc. 1948;43:572–4.

28. Lanfear R, Frandsen PB, Wright AM, Senfeld T, Calcott B. Partitionfinder 2: New methods for selecting partitioned models of evolution for molecular and morphological phylogenetic analyses. Mol Biol Evol. 2017;34:772–3.

29. Stamatakis A. RAxML version 8: A tool for phylogenetic analysis and post-analysis of large phylogenies. Bioinformatics. 2014;30:1312–3.

30. Lanfear R, Calcott B, Kainer D, Mayer C, Stamatakis A. Selecting optimal partitioning schemes for phylogenomic datasets. BMC Evol Biol. 2014;14:82. doi:10.1186/1471-2148-14-82.

31. Le SQ, Gascuel O. An improved general amino acid replacement matrix. Mol Biol Evol. 2008;25:1307–20.

32. Whelan S, Goldman N. A general empirical model of protein evolution derived from multiple protein families using a maximum-likelihood approach. Mol Biol Evol. 2001;18:691–9.

33. Kosiol C, Goldman N. Different versions of the dayhoff rate matrix. Mol Biol Evol. 2005;22:193–9.

34. Jones DT, Taylor WR, Thornton JM. The rapid generation of mutation data matrices from protein sequences. Comput Appl Biosci. 1992;8:275–82.

35. Henikoff S, Henikoff JG. Amino acid substitution matrices from protein blocks. Proc Natl Acad Sci U S A. 1992;89:10915–9.

36. Yang Z. Maximum likelihood phylogenetic estimation from DNA sequences with variable rates over sites: Approximate methods. J Mol Evol. 1994;39:306–14.

37. Le SQ, Dang CC, Gascuel O. Modeling protein evolution with several amino acid replacement matrices depending on site rates. Mol Biol Evol. 2012;29:2921–36.

38. Akaike H. A new look at the statistical model identification. IEEE Trans Automat Contr. 1974;19:716–23.

39. Hurvich CM, Tsai CL. Regression and time series model selection in small samples. Biometrika. 1989;76:297–307.

40. Frandsen PB, Calcott B, Mayer C, Lanfear R. Automatic selection of partitioning schemes for phylogenetic analyses using iterative *k*-means clustering of site rates. BMC Evol Biol. 2015;15:13.

41. Zhang C, Rabiee M, Sayyari E, Mirarab S. ASTRAL-III: Polynomial time species tree reconstruction from partially resolved gene trees. BMC Bioinformatics. 2018;19 Suppl 6:15–30.

42. Tavaré S. Some probabilistic and statistical problems in the analysis of DNA sequences. Lect Math Life Sci. 1986;17:57–86.

43. Jukes TH, Cantor CR. Evolution of protein molecules. In: Munro HNBT-MPM, editor. Mammalian protein metabolism. Academic Press; 1969. p. 21–132.

44. Felsenstein J. Evolutionary trees from DNA sequences: A maximum likelihood approach. J Mol Evol. 1981;17:368–76.

45. Kimura M. A simple method for estimating evolutionary rates of base substitutions through comparative studies of nucleotide sequences. J Mol Evol. 1980;16:111–20.

46. Hasegawa M, Kishino H, Yano T. Dating of the human-ape splitting by a molecular clock of mitochondrial DNA. J Mol Evol. 1985;22:160–74.

47. Tamura K, Nei M. Estimation of the number of nucleotide substitutions in the control region of mitochondrial DNA in humans and chimpanzees. Mol Biol Evol. 1993;10:512–26.

48. Schwarz G. Estimating the dimension of a model. Ann Stat. 1978;6:461–4.

49. Kalyaanamoorthy S, Minh BQ, Wong TKF, von Haeseler A, Jermiin LS. ModelFinder: Fast model selection for accurate phylogenetic estimates. Nat Methods. 2017;14.

50. Chernomor O, Von Haeseler A, Minh BQ. Terrace aware data structure for phylogenomic inference from supermatrices. Syst Biol. 2016;65:997–1008.

51. Lartillot N, Brinkmann H, Philippe H. Suppression of long-branch attraction artefacts in the animal phylogeny using a site-heterogeneous model. BMC Evol Biol. 2007;7 Suppl. 1:1–14.

52. Wang H-C, Minh BQ, Susko E, Roger AJ. Modeling site heterogeneity with posterior mean site frequency profiles accelerates accurate phylogenomic estimation. Syst Biol. 2017;67:216–235.

53. Quang LS, Gascuel O, Lartillot N. Empirical profile mixture models for phylogenetic reconstruction. Bioinformatics. 2008;24:2317–23.

54. Hoang DT, Chernomor O, von Haeseler A, Minh BQ, Le SV. UFBoot2: Improving the ultrafast bootstrap approximation. Mol Biol Evol. 2018;35:518–22.

55. Guindon S, Dufayard JF, Lefort V, Anisimova M, Hordijk W, Gascuel O. New algorithms and methods to estimate maximum-likelihood phylogenies: Assessing the performance of PhyML 3.0. Syst Biol. 2010;59:307–21.

56. Aberer AJ, Krompass D, Stamatakis A. Pruning rogue taxa improves phylogenetic accuracy: An efficient algorithm and webservice. Syst Biol. 2013;62:162–6.

57. Salichos L, Rokas A. Inferring ancient divergences requires genes with strong phylogenetic signals. Nature. 2013;497:327–31.

58. Vasilikopoulos A, Balke M, Beutel RG, Donath A, Podsiadlowski L, Pflug JM, et al. Phylogenomics of the superfamily Dytiscoidea (Coleoptera: Adephaga) with an evaluation of phylogenetic conflict and systematic error. Mol Phylogenet Evol. 2019;135:270–85.

59. Johnson KP, Dietrich CH, Friedrich F, Beutel RG, Wipfler B, Peters RS, et al. Phylogenomics and the evolution of hemipteroid insects. Proc Natl Acad Sci. 2018;115:12775–80.

60. Sann M, Niehuis O, Peters RS, Mayer C, Kozlov A, Podsiadlowski L, et al. Phylogenomic analysis of Apoidea sheds new light on the sister group of bees. BMC Evol Biol. 2018;18:1–15.

61. Jandausch K, Pohl H, Aspöck U, Winterton SL, Beutel RG. Morphology of the primary larva of *Mantispa aphavexelte* Aspöck & Aspöck, 1994 (Neuroptera: Mantispidae) and phylogenetic implications to the order of Neuroptera. Arthropod Syst Phylogeny. 2018;76:529–60.

62. Aspöck U, Plant JD, Nemeschkal HL. Cladistic analysis of Neuroptera and their systematic position within Neuropterida (Insecta: Holometabola: Neuropterida: Neuroptera). Syst Entomol. 2001;26:73–86.

63. Aspöck U, Aspöck H. Phylogenetic relevance of the genital sclerites of Neuropterida (Insecta: Holometabola). Syst Entomol. 2008;33:97–127.

64. Winterton SL, Lemmon AR, Gillung JP, Garzon IJ, Badano D, Bakkes DK, et al. Evolution of lacewings and allied orders using anchored phylogenomics (Neuroptera, Megaloptera, Raphidioptera). Syst Entomol. 2018;43:330–354.

65. Machado RJP, Gillung JP, Winterton SL, Garzón-Orduña IJ, Lemmon AR, Lemmon EM, et al. Owlflies are derived antlions: anchored phylogenomics supports a new phylogeny and classification of Myrmeleontidae (Neuroptera). Syst Entomol. 2019;44:418–450.

66. Inoue J, Donoghue PCJ, Yang Z. The impact of the representation of fossil calibrations on Bayesian estimation of species divergence times. 2010;59:74–89.

67. Carpenter FM. The Lower Permian insects of Kansas. Pan 12. Protorthoptera (continued), Neuroptera, additional Palaeodictyoptera, and families of uncertain position. Psyche. 1976;83:336–376.

68. Prokop J, Rodrigues Fernandes F, Lapeyrie J, Nel A. Discovery of the first lacewings (Neuroptera: Permithonidae) from the guadalupian of the Lodève basin (Southern France). Geobios. 2015;48:263–70.

69. Wang YH, Engel MS, Rafael JA, Wu HY, Rédei D, Xie Q, et al. Fossil record of stem groups employed in evaluating the chronogram of insects (Arthropoda: Hexapoda). Sci Rep. 2016;6:1–12.

70. Engel MS, Winterton SL, Breitkreuz LCV. Phylogeny and evolution of Neuropterida: Where have wings of lace taken us? Annu Rev Entomol. 2018;63:531–51.

71. Nel A, Roques P, Nel P, Prokop J, Steyer JS. The earliest holometabolous insect from the Carboniferous: a “crucial” innovation with delayed success (Insecta Protomeropina Protomeropidae). Ann la Société Entomol Fr. 2007;43:349–55.

72. Wolfe JM, Daley AC, Legg DA, Edgecombe GD. Fossil calibrations for the arthropod tree of life. Earth-Science Rev. 2016;160 July:43–110.

73. Ponomarenko AG. Suborder Adephaga, Polyphaga Incertae Sedis, Infraorder Staphyliniformia. In: Mesozoic Coleoptera. 161st edition. Tr. Paleont. Inst. Akad. Nauk SSSR 161; 1977. p. 17–119.

74. Tillyard RJ. Kansas Permian insects; Part 7, The order Mecoptera. Am J Sci. 1926;11:133–164.

75. Handlirsch A. Palaeontologie. Handbuch der Entomologie, 3. 1920.

76. Liu X, Hayashi F, Yang D. Phylogeny of the family Sialidae (Insecta: Megaloptera) inferred from morphological data, with implications for generic classification and historical biogeography. Cladistics. 2014;31:18–49.

77. Ansorge J, Schlüter T. The earliest chrysopid: *Liassochrysa stigmatica* n.g., n. sp. from the Lower Jurassic of Dobbertin, Germany. Neuroptera Int. 1990;6:87–93.

78. Macchioni F. Myths and legends in the correlation between the Boreal and Tethyan Realms. Implications on the dating of the Early Toarcian mass extinctions and the oceanic anoxic event. Geobios. 2002;35 Suppl. 1:150–164

79. Ogg JG, Hinnov LA, Huang C. Chapter 26 - Jurassic. In: Gradstein FM, Ogg JG, Schmitz MD, Ogg GM, editors. The geologic time scale. Boston: Elsevier; 2012. p. 731–91.

80. Ren D, Labandeira CC, Santiago-Blay JA, Rasnitsyn A, Shih C, Bashkuev A, et al. A probable pollination mode before angiosperms: Eurasian, long-proboscid scorpionflies. Science. 2009;326:840–847.

81. Azar D, Nel A, Solignac M. A new Coniopterygidae from Lebanese amber. Acta Geol Hisp. 2000;35:31–6.

82. Maksoud S, Azar D, Granier B, Gèze R. New data on the age of the Lower Cretaceous

amber outcrops of Lebanon. Palaeoworld. 2017;26:331–8.

83. Wichard W. Family Nevrorthidae (Insecta, Neuroptera) in mid-Cretaceous Burmese amber. Palaeodiversity. 2017;10:1–5.

84. Makarkin VN, Perkovsky E. *Rophalis relicta* Hagen (Neuroptera, Nevrorthidae) in the Late Eocene Rovno amber, with a discussion of the taxonomic status of the genus. Denisia. 2009;86:137–144.

85. Wichard W. Overview and descriptions of Nevrorthidae in Baltic amber (Insecta, Neuroptera). Palaeodiversity. 2016;9:95–111.

86. Shi G, Grimaldi DA, Harlow GE, Wang J, Wang J, Yang M, et al. Age constraint on Burmese amber based on U–Pb dating of zircons. Cretac Res. 2012;37:155–63.

87. Panfilov D V. Novye predstaviteli setcharokrylykh (Neuroptera) iz yury Karatau [New representatives of lacewings (Neuroptera) from the Jurassic of Karatau]. In: Dolin, V. G., Panfilov DV et al., editor. Iskopaemye nasekomye mezozoya (Fossil insects of the Mesozoic). Kiev: Naukova Dumka; 1980. p. 82–111.

88. Khramov A V, Liu Q, Zhang H, Jarzembowski EA. Early green lacewings (Insecta: Neuroptera: Chrysopidae) from the Jurassic of China and Kazakhstan. Pap Palaeontol. 2015;2:25–39.

89. Nel A, Delclos X, Hutin A. Mesozoic chrysopid-like Planipennia: A phylogenetic approach (Insecta: Neuroptera). Ann la Société Entomol Fr. 2005;41:29–69.

90. Archibald SB, Makarkin VN. A diverse new assemblage of green lacewings (Insecta, Neuroptera, Chrysopidae) from the early Eocene Okanagan Highlands, western North America. J Paleontol. 2013;87:123–46.

91. Archibald SB, Makarkin VN. A new species of *Archaeochrysa* Adams (Neuroptera: Chrysopidae) from the early Eocene of Driftwood Canyon, British Columbia, Canada. Can Entomol. 2015;147:359–69.

92. Dai Y, Winterton SL, Garzón-Orduña IJ, Liang F, Liu X. Mitochondrial phylogenomic analysis resolves the subfamily placement of enigmatic green lacewing genus *Nothancyla* (Neuroptera: Chrysopidae). Austral Entomol. 2016;56:322–31.

93. Winterton SL, Hardy NB, Wiegmann BM. On wings of lace: Phylogeny and Bayesian divergence time estimates of Neuropterida (Insecta) based on morphological and molecular data. Syst Entomol. 2010;35:349–78.

94. Ren D, Guo ZG. On the new fossil genera and species of Neuroptera (Insecta) from the Late Jurassic of northeast China. Acta Zootaxonomica Sin. 1996;21:461–79.

95. Jepson JE, Makarkin VN, Coram RA. Lacewings (Insecta: Neuroptera) from the Lower Cretaceous Purbeck limestone group of southern England. Cretac Res. 2012;34:31–47.

96. Gao K-Q, Ren D. Radiometric dating of Ignimbrite from inner Mongolia provides no

indication of a post-Middle Jurassic age for the Daohugou Beds. Acta Geol Sin - English Ed. 2006;80:42–5.

97. Wang X, Zhou Z, He H, Jin F, Wang Y, Zhang J, et al. Stratigraphy and age of the Daohugou Bed in Ningcheng, inner Mongolia. Chinese Sci Bull. 2005;50:2369–76.

98. Zhang J. Archisargoid flies (Diptera, Brachycera, Archisargidae and Kovalevisargidae) from the Jurassic Daohugou biota of China, and the related biostratigraphical correlation and geological age. J Syst Palaeontol. 2015;13:857–81.

99. Wang X, Wang Y, Zhang F, Zhang J, Zhou Z, Jin F, et al. Vertebrate biostratigraphy of the Lower Cretaceous Yixian Formation in Lingyuan, western Liaoning and its neighboring southern Nei Mongol (Inner Mongolia), China. Vertebr Palasiat. 2000;38:81–99.

100. Chang S, Zhang H, Renne PR, Fang Y. High-precision 40Ar/39Ar age for the Jehol Biota. Palaeogeogr Palaeoclimatol Palaeoecol. 2009;280:94–104.

101. He HY, Wang XL, Zhou ZH, Zhu RX, Jin F, Wang F, et al. 40Ar/39Ar dating of ignimbrite from Inner Mongolia, northeastern China, indicates a post-Middle Jurassic age for the overlying Daohugou Bed. Geophys Res Lett. 2004;31:L20609.

102. Peng N, Liu Y, Kuang H, Jiang X, Xu H. Stratigraphy and geochronology of vertebrate fossil bearing Jurassic strata from Linglongta, Jianchang county, western Liaoning, Northeastern China. Acta Geologica Sinica - English Ed. 2012;86:1326–1339

103. Oswald JD. Revision and cladistic analysis of the world genera of the family Hemerobiidae (Insecta: Neuroptera). Journal of the New York Ent. Soc. 1993;101:143–296

104. Makarkin VN, Archibald SB, Oswald JD. New Early Eocene brown lacewings (Neuroptera: Hemerobiidae) from western North America. Can Entomol. 2003;135:637–53.

105. Ponomarenko AG. Novye setchatokrylye (Insecta: Neuroptera) iz mezozoya Mongolii. Novye Taksony Iskopaemykh Bespozvonochnykh Mongolii, Sovmestnaya Rossiisko-Mongol’skaya. Paleontol Ekspeditsiya. 1992;41:101–111.

106. Garzón-Orduña IJ, Menchaca-Armenta I, Contreras-Ramos A, Liu X, Winterton SL. The phylogeny of brown lacewings (Neuroptera: Hemerobiidae) reveals multiple reductions in wing venation. BMC Evol Biol. 2016;16:1–19.

107. Doludenko MP, Orlovskaya ER. Jurassic flora of Karatau. Moscow: Nauka; 1976.

108. Doludenko MP, Sakulina G V., Ponomarenko AG. The geology and Late Jurassic fauna and flora of a unique locality Aulie (Karatau Mountains, southern Kazakhstan). Moscow: Geologicheskii Institut AN SSSR; 1990.

109. Zheng B, Ren D, Wang Y. Earliest true moth lacewing from the Middle Jurassic of inner Mongolia, China. Acta Palaeontol Pol. 2016;61:847–51.

110. Liu Q, Zheng D, Zhang Q, Wang B, Fang Y, Zhang H. Two new kalligrammatids (Insecta, Neuroptera) from the Middle Jurassic of Daohugou, inner Mongolia, China. 2014.

111. Martins-Neto RG, Vulcano M. Neurópteros (Insecta, Planipennia) da Formação Santana (Cretáceo Inferior), Bacia do Araripe, nordeste do Brasil. II. Superfamília Myrmeleontoidea. Rev Bras Entomol. 1989;33:367–402.

112. Michel B, Clamens AL, Béthoux O, Kergoat GJ, Condamine FL. A first higher-level time-calibrated phylogeny of antlions (Neuroptera: Myrmeleontidae). Mol Phylogenet Evol. 2017;107:103–16.

113. Huang D, Azar D, Cai C, Garrouste R, Nel A. The first Mesozoic pleasing lacewing (Neuroptera: Dilaridae). Cretac Res. 2015;56:274–7.

114. Pons D, Berthou PY, Campos D d. A. Quelques observations sur la palynologie del'Aptien Supérieur et de l'Albien du bassin d'Araripe (NE du Brésil). Atas Do. 1990;1:241–252.

115. Zhonghe Z. Evolutionary radiation of the Jehol biota: Chronological and ecological perspectives. Geol J. 2006;41:377–93.

116. Bollback JP. SIMMAP: Stochastic character mapping of discrete traits on phylogenies. BMC Bioinformatics. 2006;7:1–7.

117. Huelsenbeck JP, Nielsen R, Bollback JP. Stochastic mapping of morphological characters. Syst Biol. 2003;52:131–58.

118. Revell LJ. phytools: An R package for phylogenetic comparative biology (and other things). Methods Ecol Evol. 2012;3:217–23.

119. Lawrence JF, Ślipiński A, Beutel RG, Newton AF. *Lepicerus* larva still unknown: A correction (Coleoptera: Lepiceridae, Phalacridae). Zootaxa. 2019;4545:441–2.

120. Pennell MW, Eastman JM, Slater GJ, Brown JW, Uyeda JC, Fitzjohn RG, et al. Geiger v2.0: An expanded suite of methods for fitting macroevolutionary models to phylogenetic trees. Bioinformatics. 2014;30:2216–8.

121. Paradis E, Schliep K. ape 5.0: An environment for modern phylogenetics and evolutionary analyses in R. Bioinformatics. 2018;35:526–8.

122. Badano D, Aspöck U, Aspöck H, Cerretti P. Phylogeny of Myrmeleontiformia based on larval morphology (Neuropterida: Neuroptera). Syst Entomol. 2017;42:94–117.

123. Sayyari E, Mirarab S. Fast coalescent-based computation of local branch support from quartet frequencies. Mol Biol Evol. 2016;33:1654–68.
