# Supplementary material for: An integrative phylogenomic approach to elucidate the evolutionary history and divergence times of Neuropterida (Insecta: Holometabola)
Source: BMC Evol Biol. 2020 Jun 3;20:64. doi: 10.1186/s12862-020-01631-6 (PMC7268685; doi:10.1186/s12862-020-01631-6)
Supplement: Supplementary file 3 — Additional file 3: Supplementary Figures S1–S56. The supplementary figures include: 1) all phylogenetic trees inferred from the analyses of different datasets and tree-inference methods, 2) results of additional ACSR analyses under different parameters, 3) heatmaps visualizing the pairwise alignment completeness scores of all analyzed supermatrices, 4) heatmaps visualizing the pairwise deviation from SRH conditions in each analyzed supermatrix, 5) scatter plot of the mean posterior node-age estimates from run 1 plotted against the mean posterior node-age estimates from run 2 when using all fossil calibrations, 6) beanplots of median posterior node-age estimates from run 1 and from run 2 when using all fossil calibrations, 7) scatter plots of the mean posterior node-age estimates plotted against the 95% higher posterior density CI-width of each node when running the dating analyses with or without data. [file 12862_2020_1631_MOESM3_ESM.pdf]

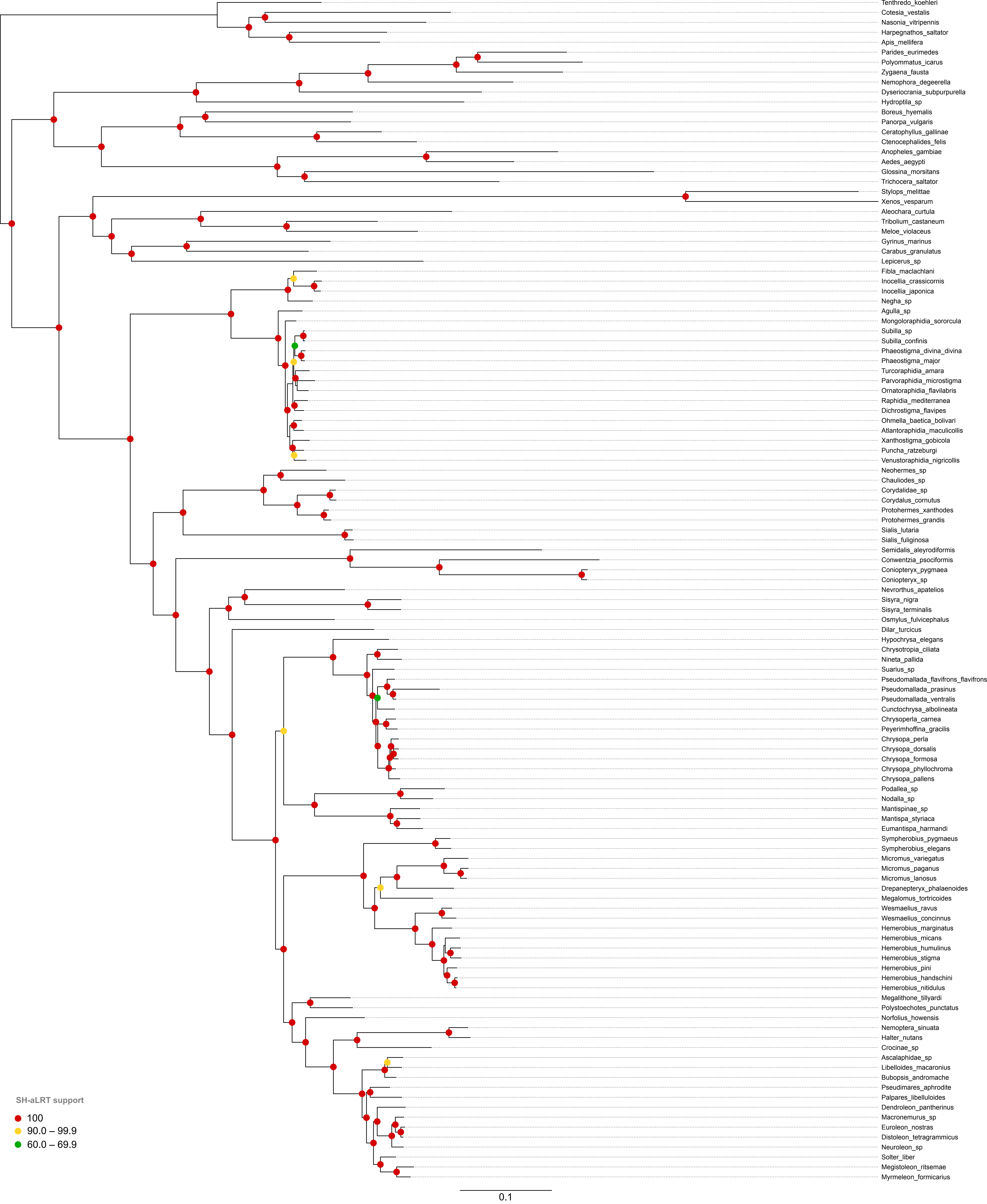

Figure S1: Phylogenetic tree with the highest log-likelihood score that resulted from the partitioned concatenated analysis of the amino-acid supermatrix E. Colored circles indicate branch support based on 10,000 SH-aLRT replicates.

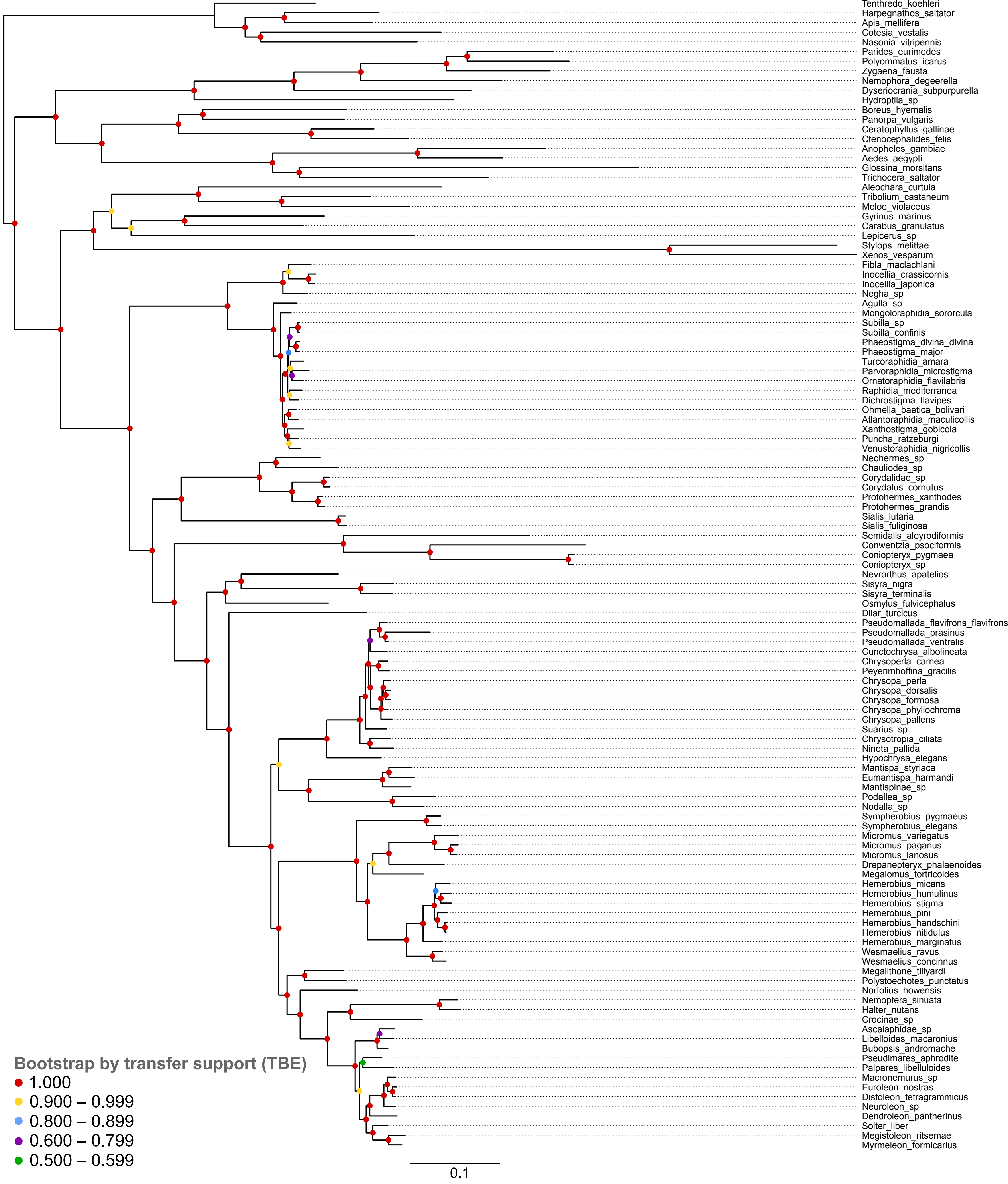

Figure S2: Phylogenetic tree with the highest log-likelihood score that resulted from the partitioned concatenated analysis of the amino-acid supermatrix E. Colored circles indicate TBE branch support based on 100 non-parametric bootstrap replicates.

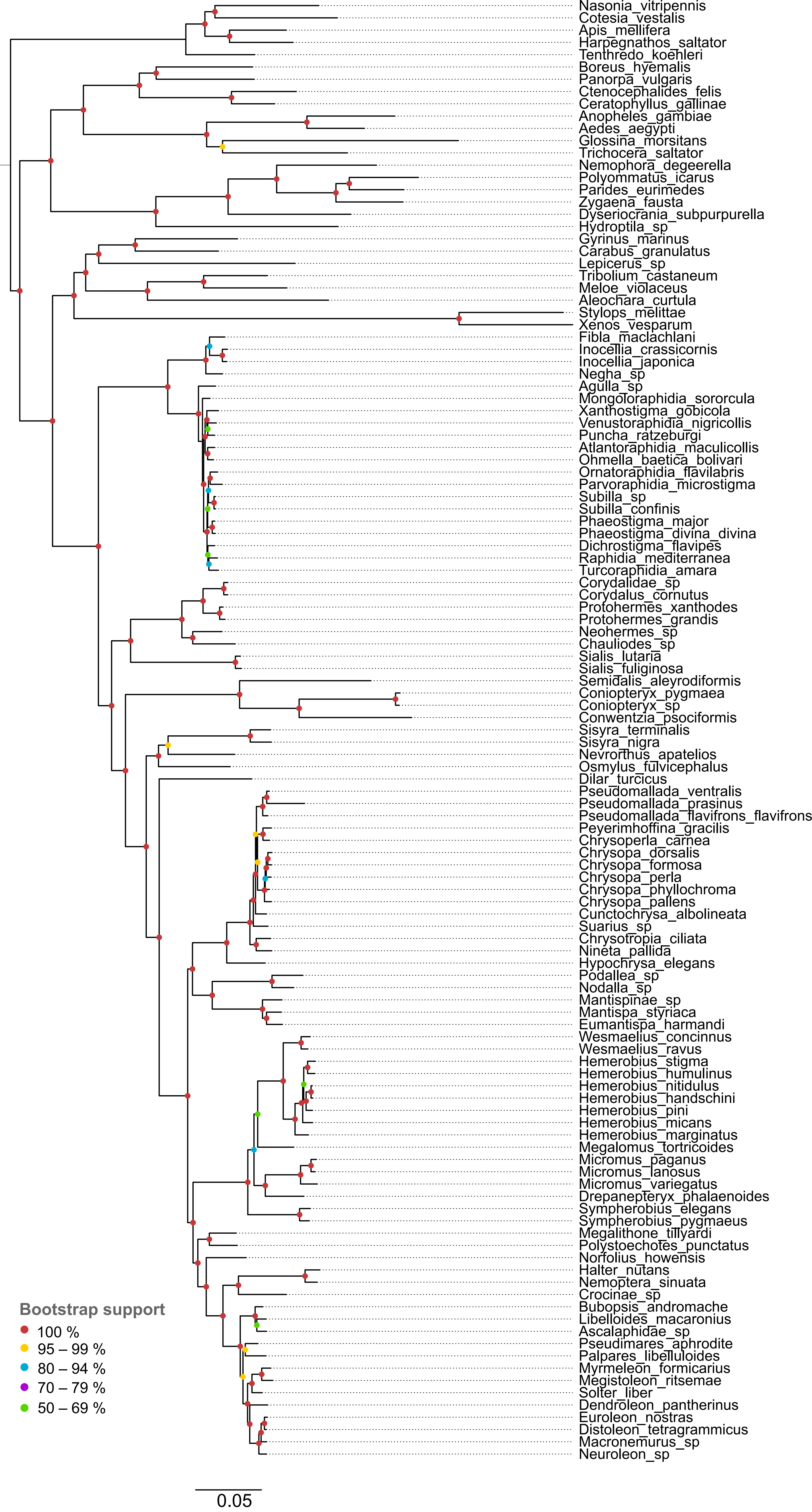

Figure S3: Phylogenetic tree with the highest log-likelihood score that resulted from the partitioned concatenated analysis of the nucleotide supermatrix D-nt. Colored circles indicate branch support based on 100 non-parametric bootstrap replicates.

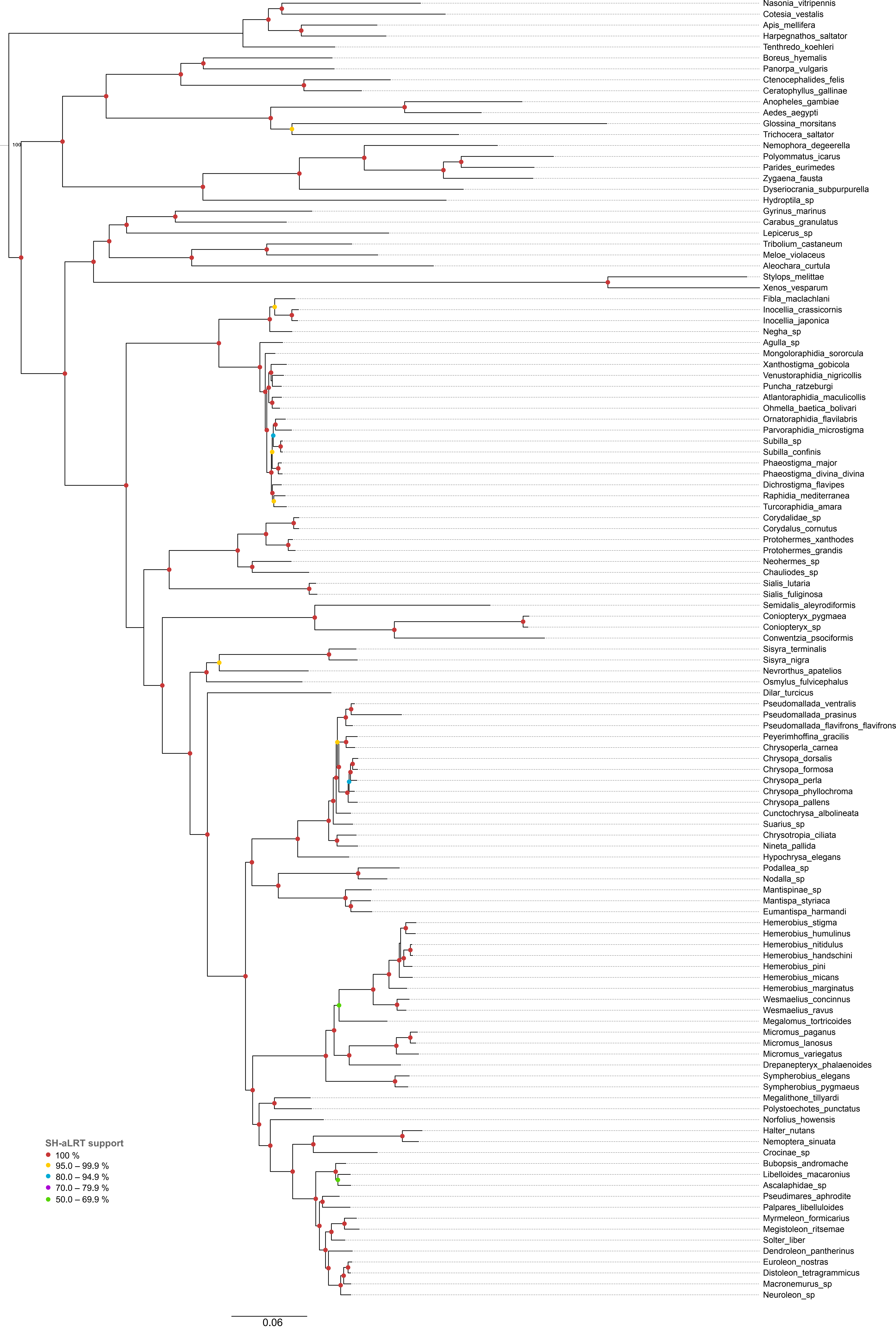

Figure S4: Phylogenetic tree with the highest log-likelihood score that resulted from the partitioned concatenated analysis of the nucleotide supermatrix D-nt. Colored circles indicate branch support based on 10,000 SH-aLRT replicates.

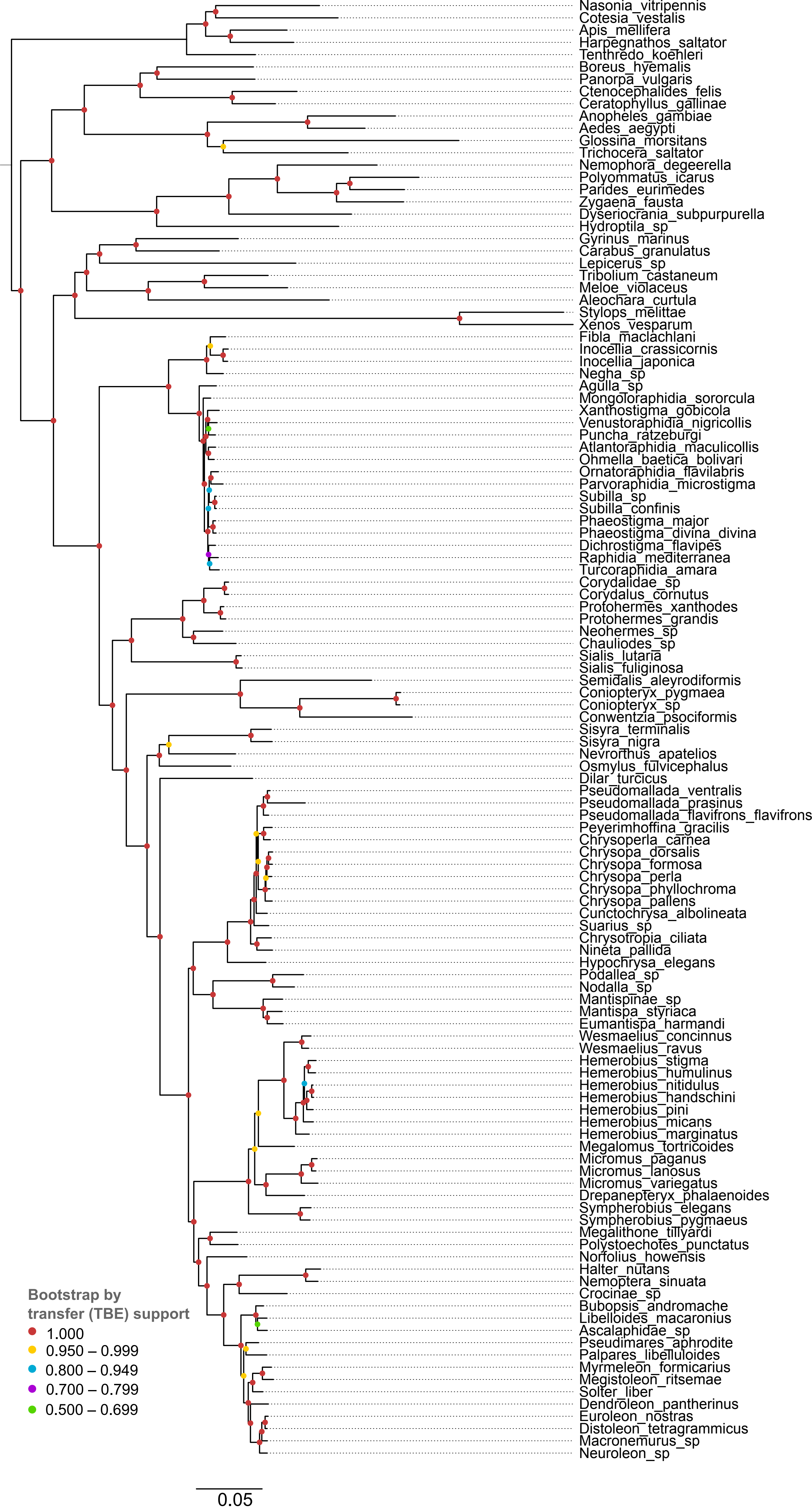

Figure S5: Phylogenetic tree with the highest log-likelihood score that resulted from the partitioned concatenated analysis of the nucleotide supermatrix D-nt. Colored circles indicate TBE support based on 100 non-parametric bootstrap replicates.

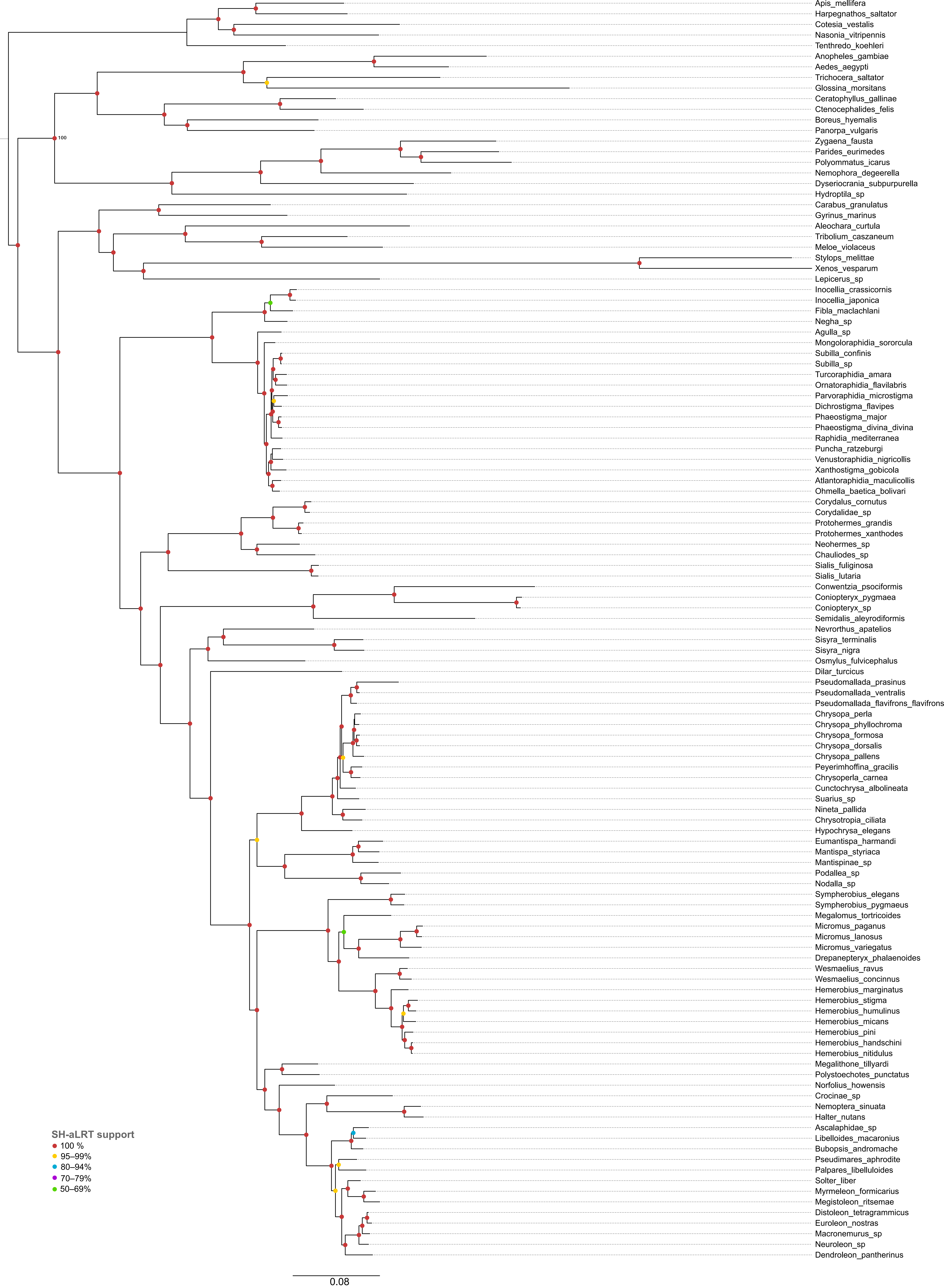

Figure S6: Phylogenetic tree with the highest log-likelihood score that resulted from the partitioned concatenated analysis of the amino-acid supermatrix E (RCFV-corrected). Colored circles indicate branch support based on 10,000 SH-aLRT replicates.

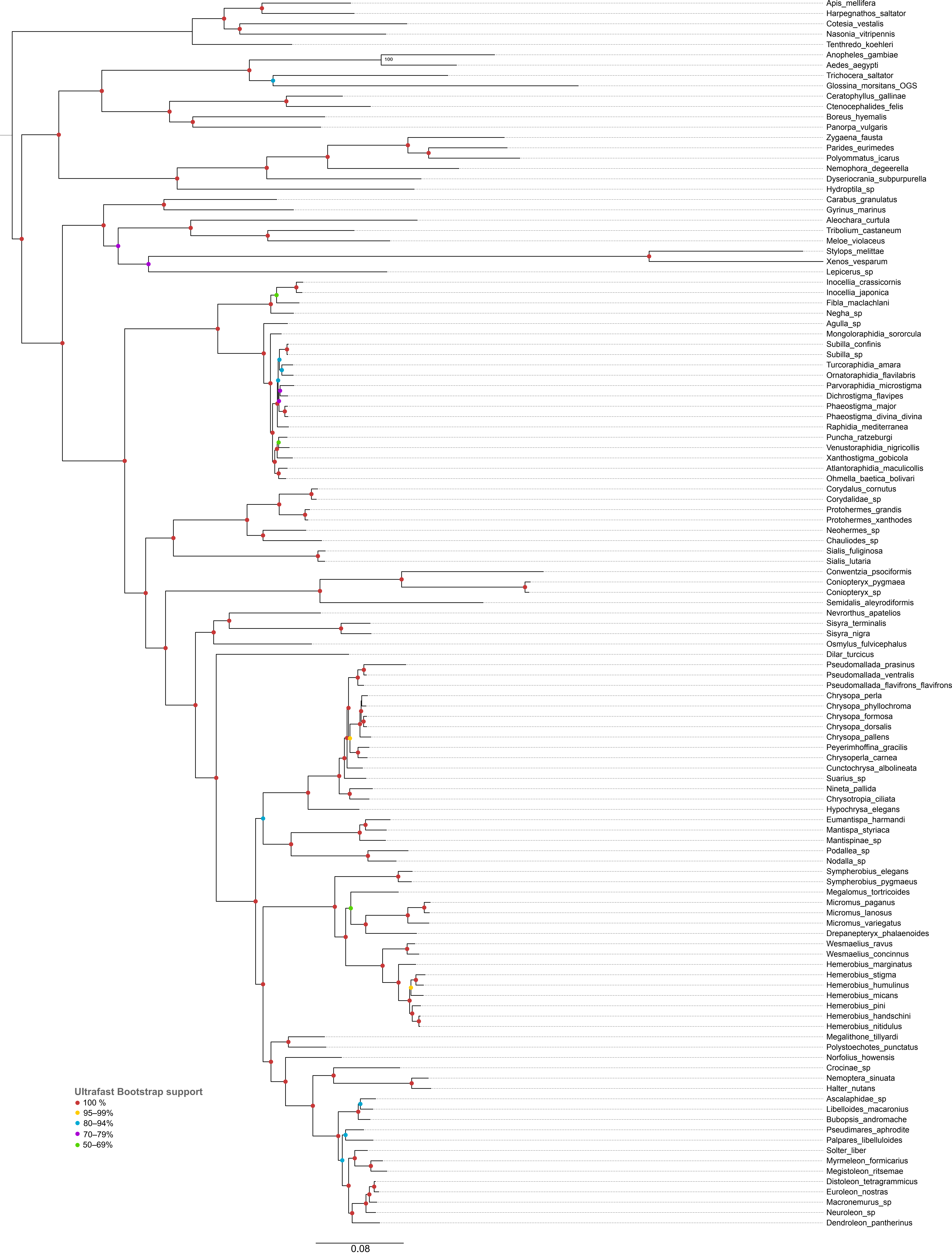

Figure S7: Phylogenetic tree with the highest log-likelihood score that resulted from the partitioned concatenated analysis of the amino-acid supermatrix E (RCFV-corrected) when calculating UFB support. Colored circles indicate UFB support based on 1,000 replicates.

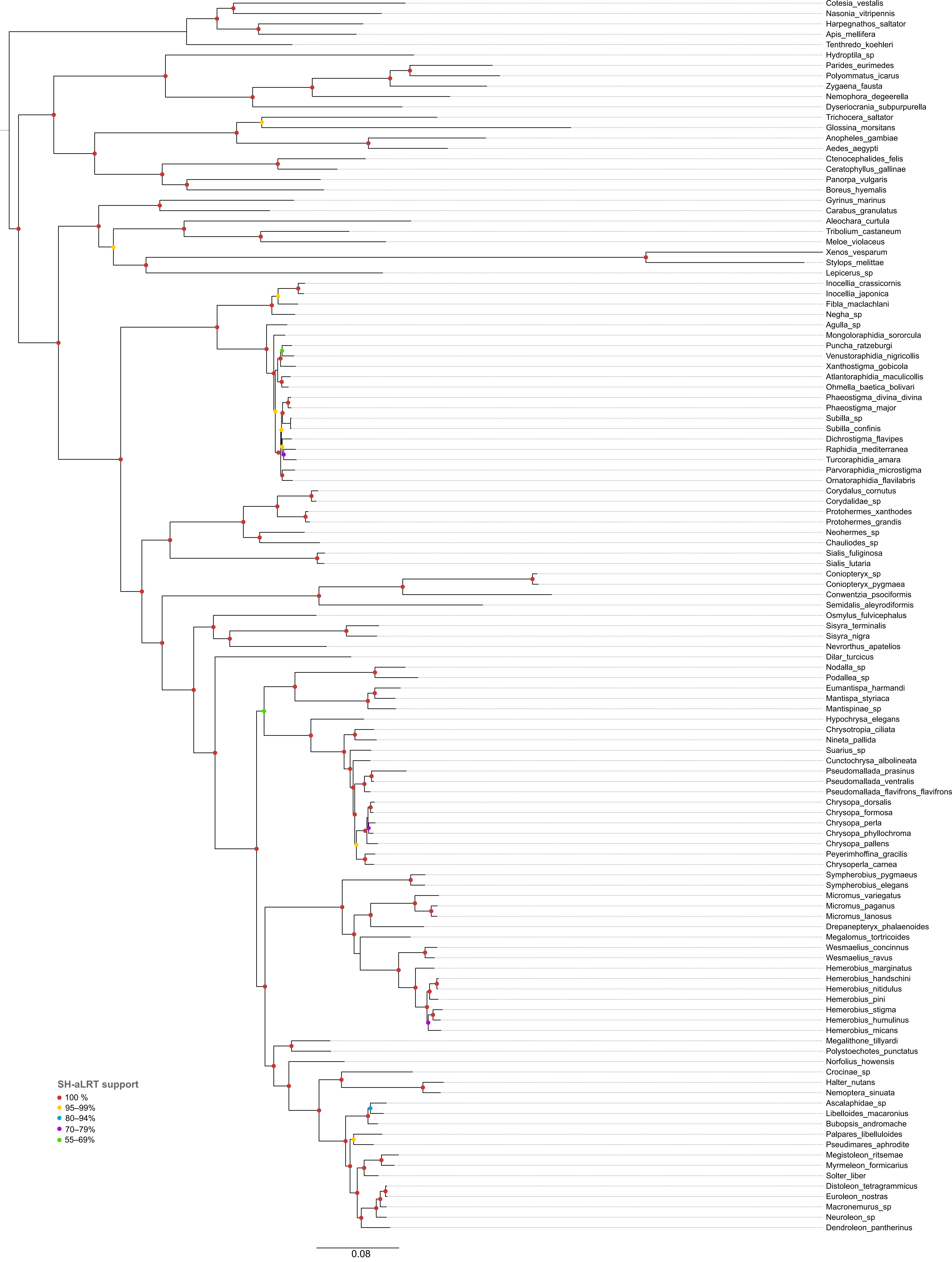

Figure S8: Phylogenetic tree with the highest log-likelihood score that resulted from the partitioned concatenated analysis of the amino-acid supermatrix E (decisive). Colored circles indicate branch support based on 10,000 SH-aLRT replicates.

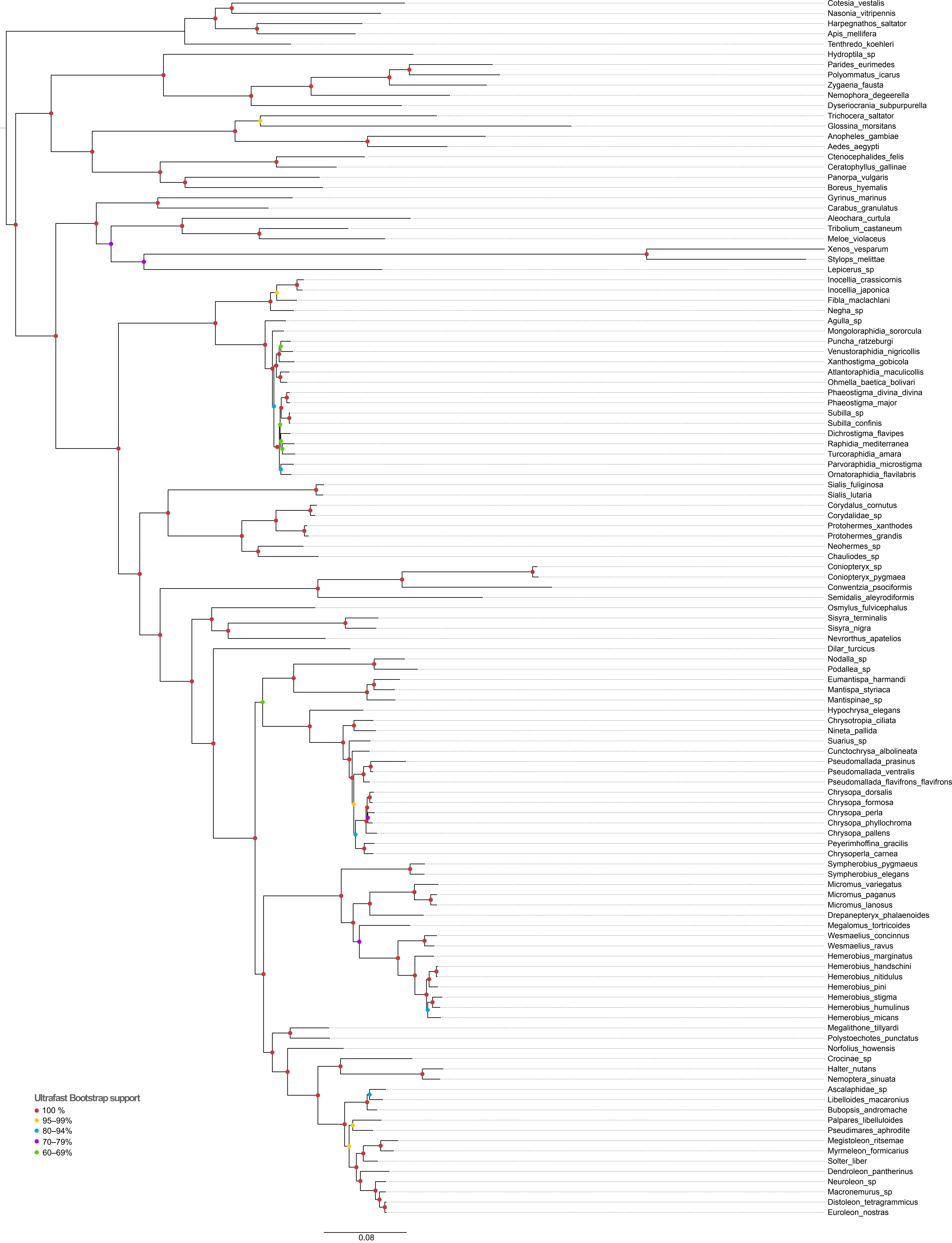

Figure S9: Phylogenetic tree with the highest log-likelihood score that resulted from the partitioned concatenated analysis of the amino-acid supermatrix E (decisive) when calculating UFB support. Colored circles indicate UFB support based on 1,000 replicates.

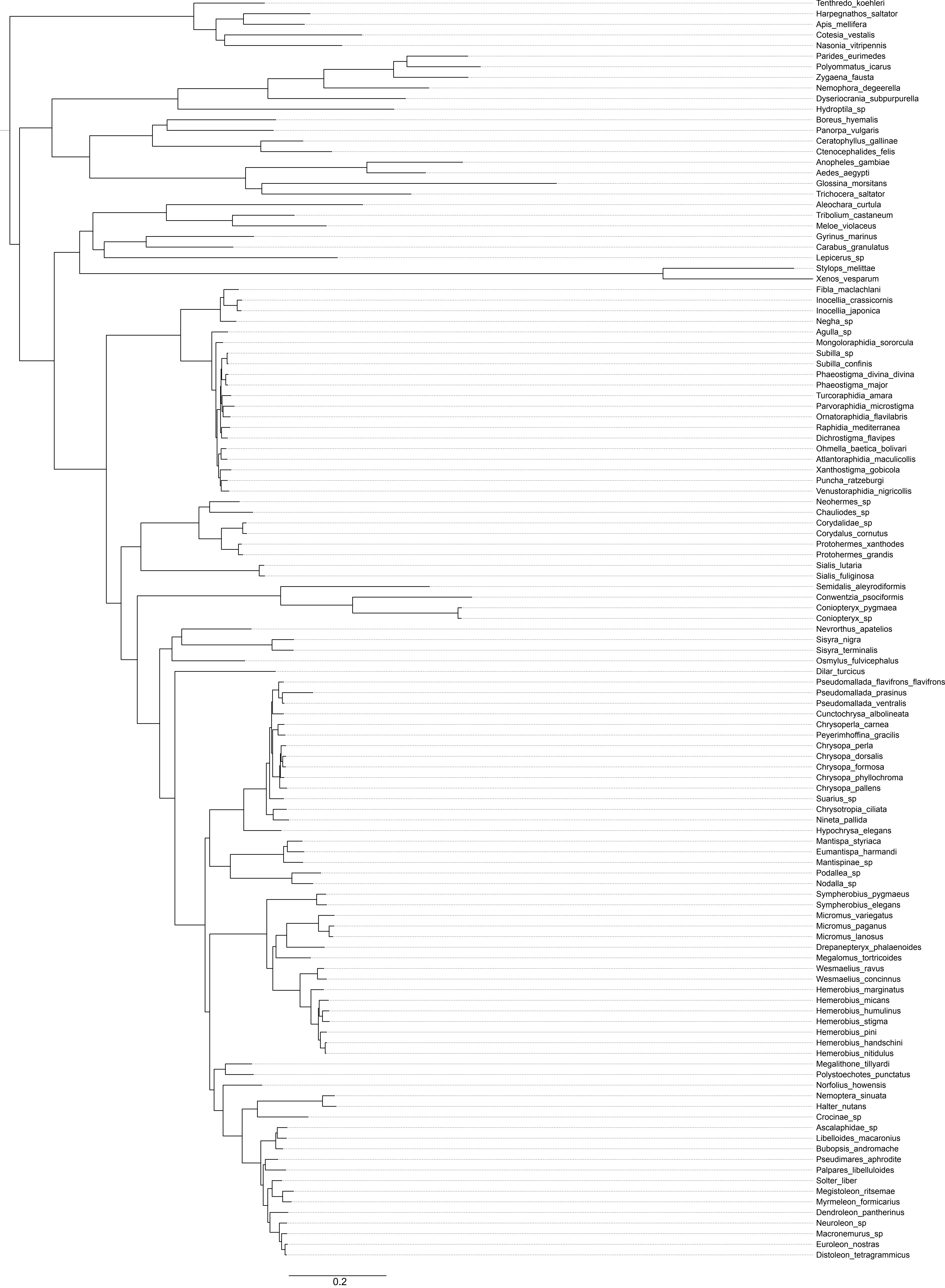

Figure S10: Phylogenetic tree that resulted from the unpartitioned phylogenetic analysis of the amino-acid supermatrix E when using the site-heterogeneous PMSF mixture model (JTT+C60+F+G).

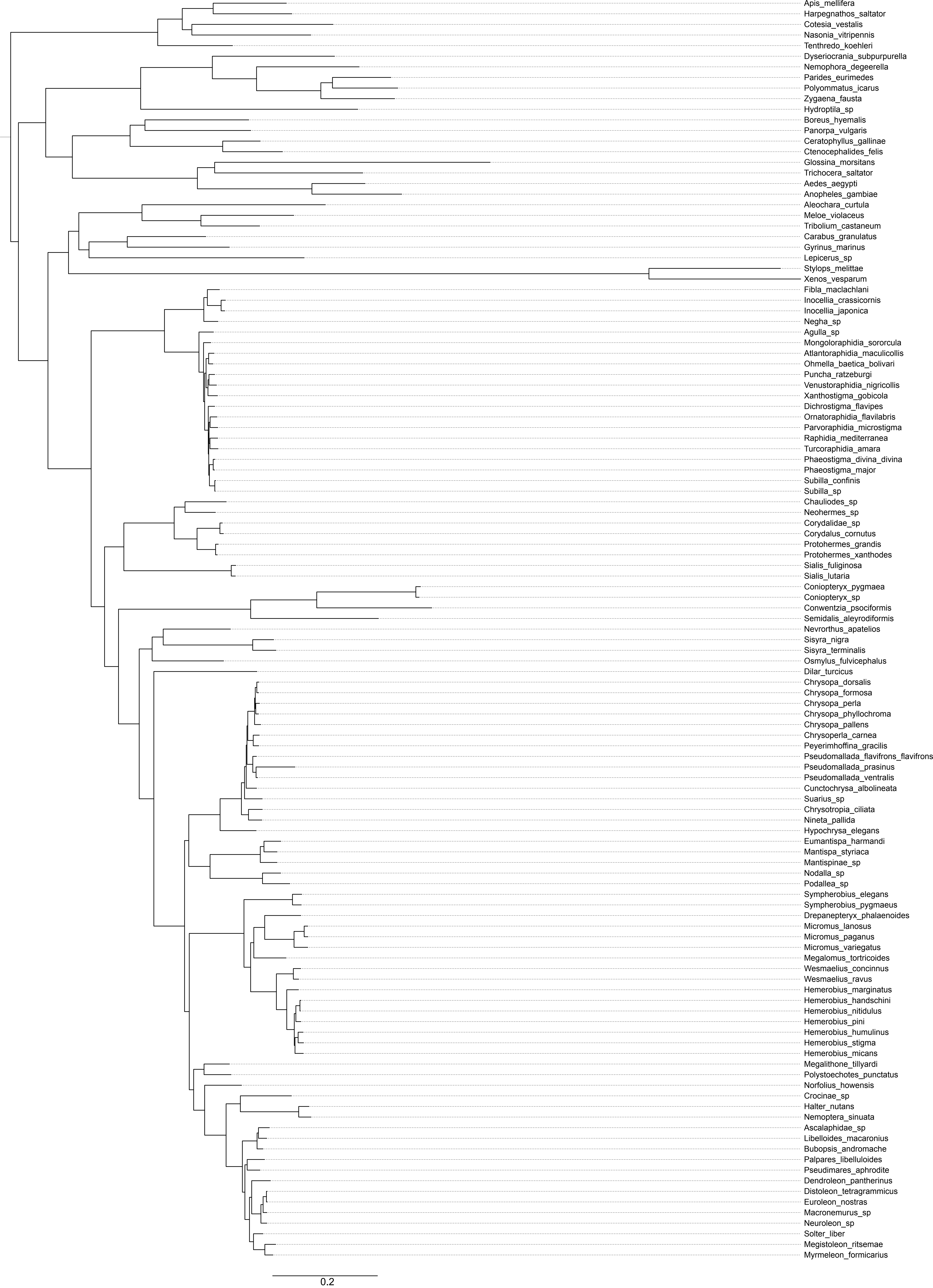

Figure S11: Phylogenetic tree that resulted from the unpartitioned phylogenetic analysis of the amino-acid supermatrix E-90 when using the site-heterogeneous PMSF mixture model (JTT+C60+F+G).

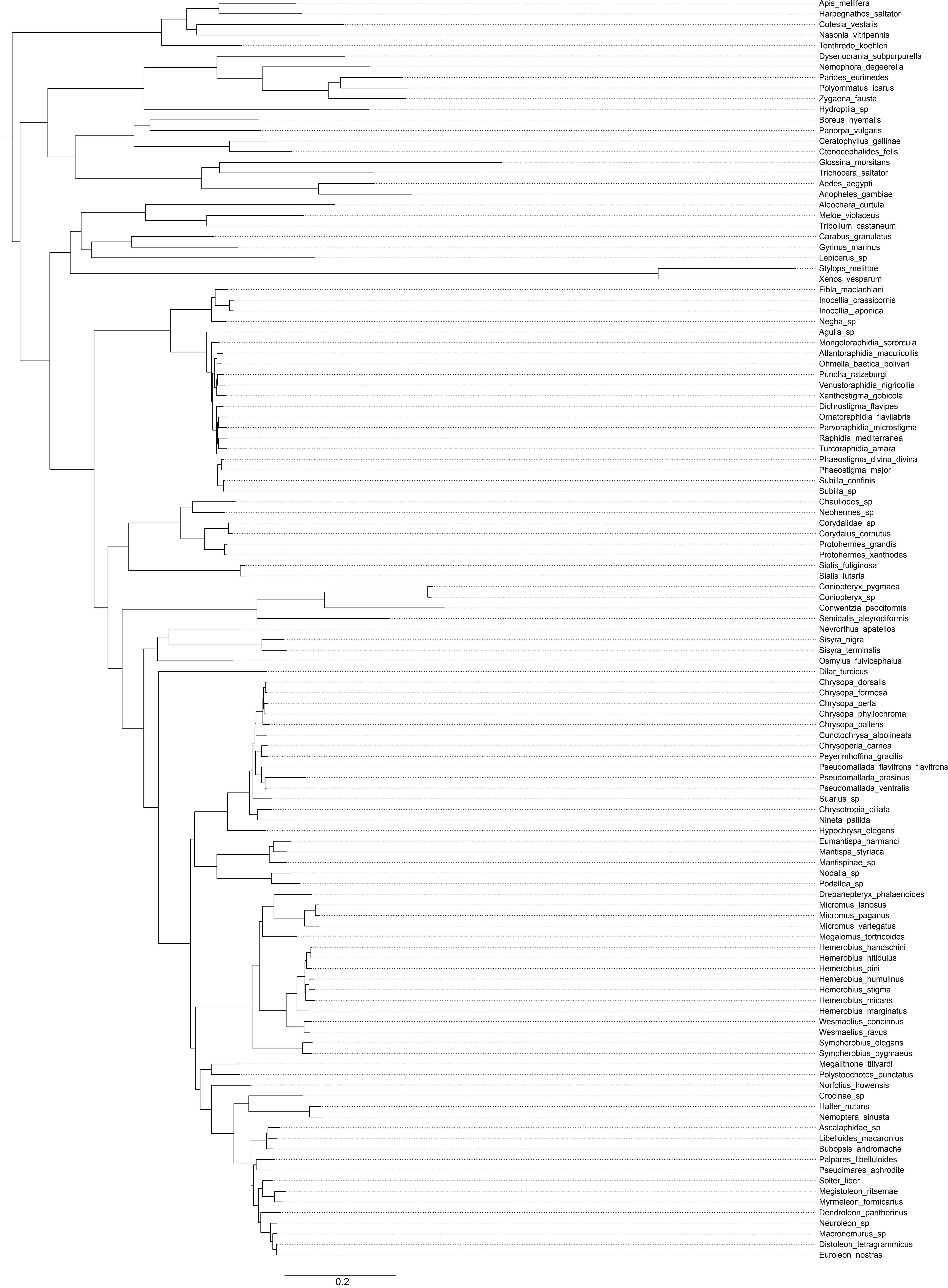

Figure S12: Phylogenetic tree that resulted from the unpartitioned phylogenetic analysis of the amino-acid supermatrix E-90 when using the site-heterogeneous PMSF mixture model (LG+C60+F+G).

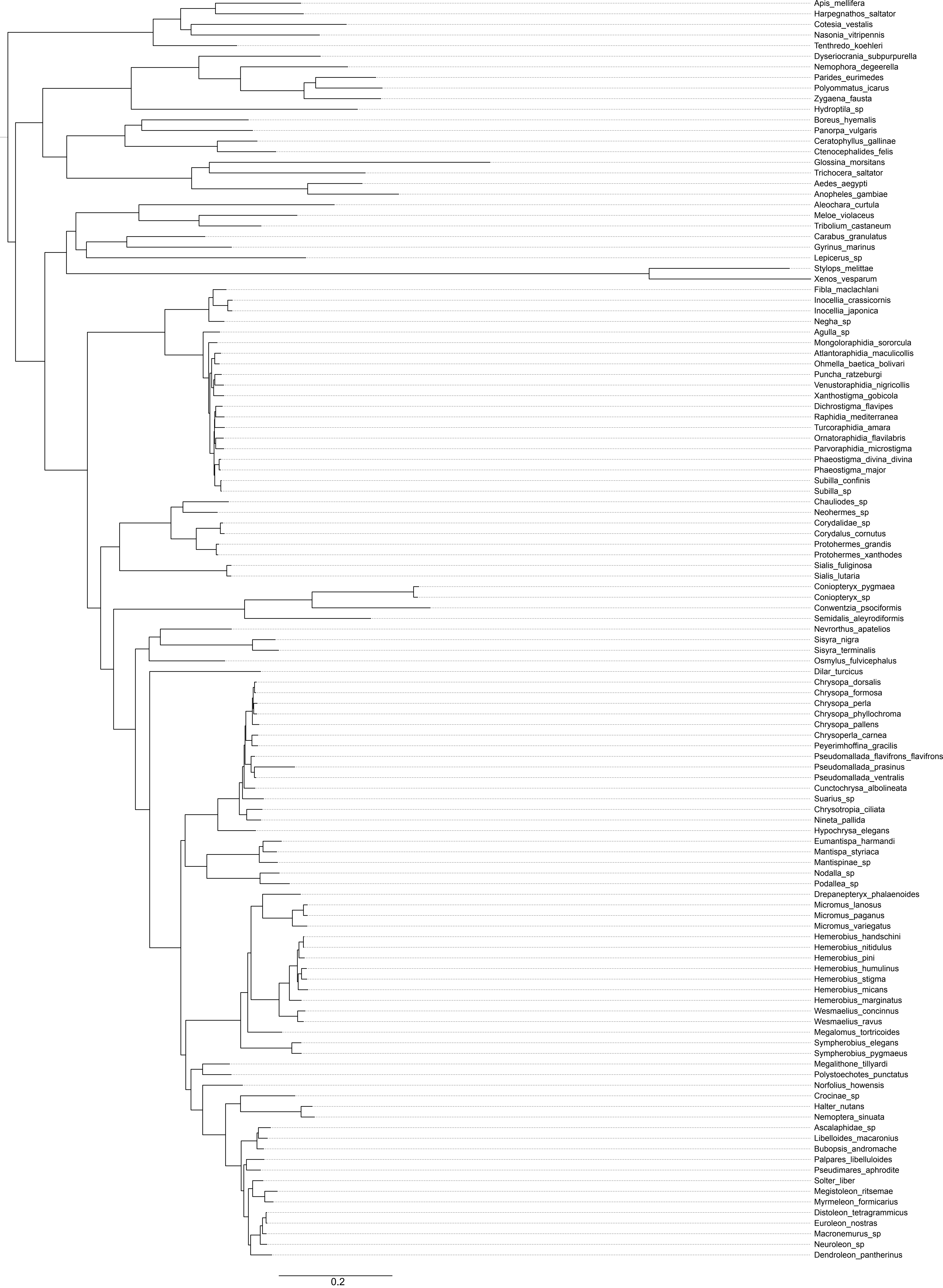

Figure S13: Phylogenetic tree that resulted from the unpartitioned phylogenetic analysis of the amino-acid supermatrix E-95 when using the site-heterogeneous PMSF mixture model (JTT+C60+F+G).

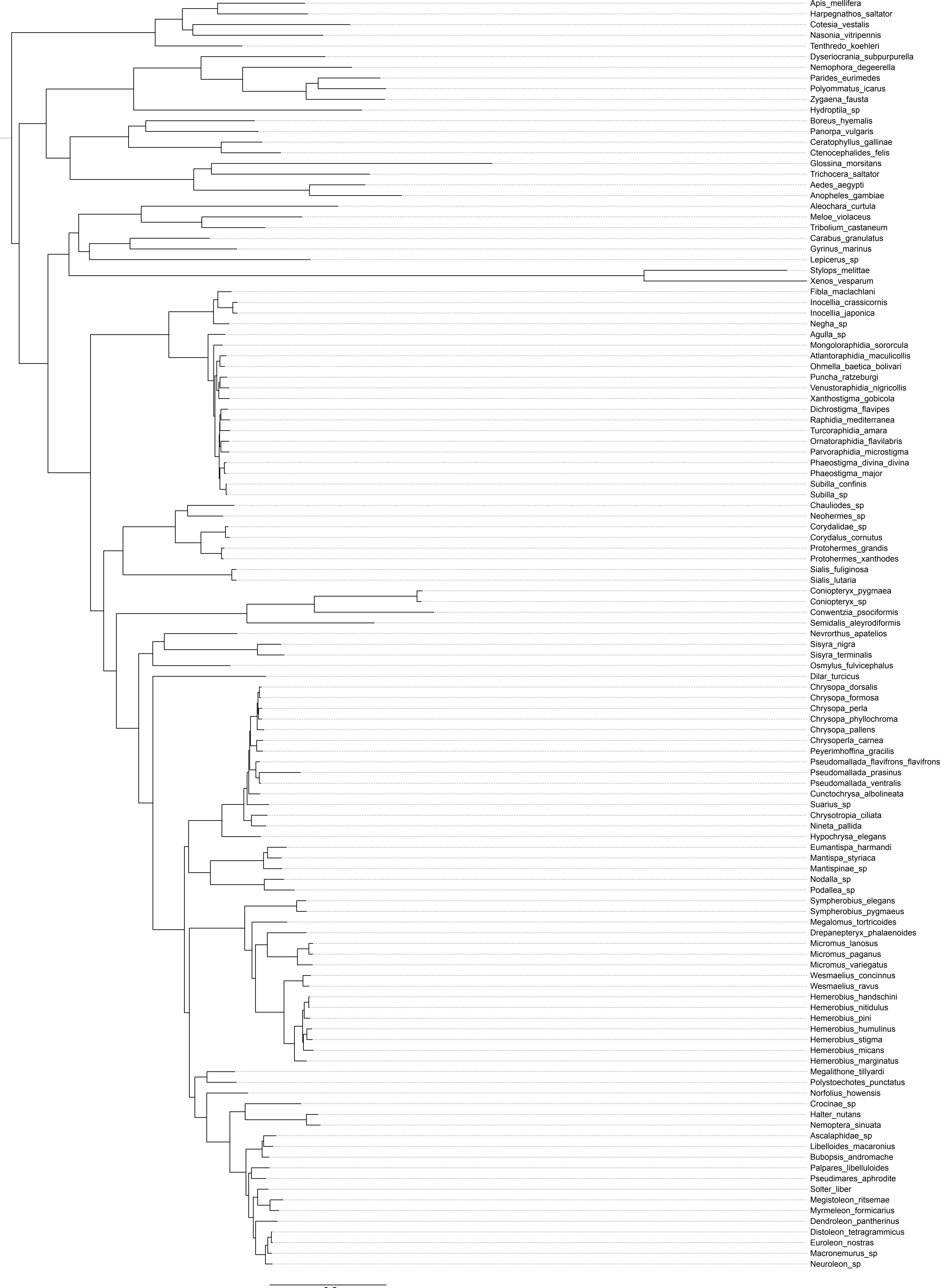

Figure S14: Phylogenetic tree that resulted from the unpartitioned phylogenetic analysis of the amino-acid supermatrix E-95 when using the site-heterogeneous PMSF mixture model (LG+C60+F+G).

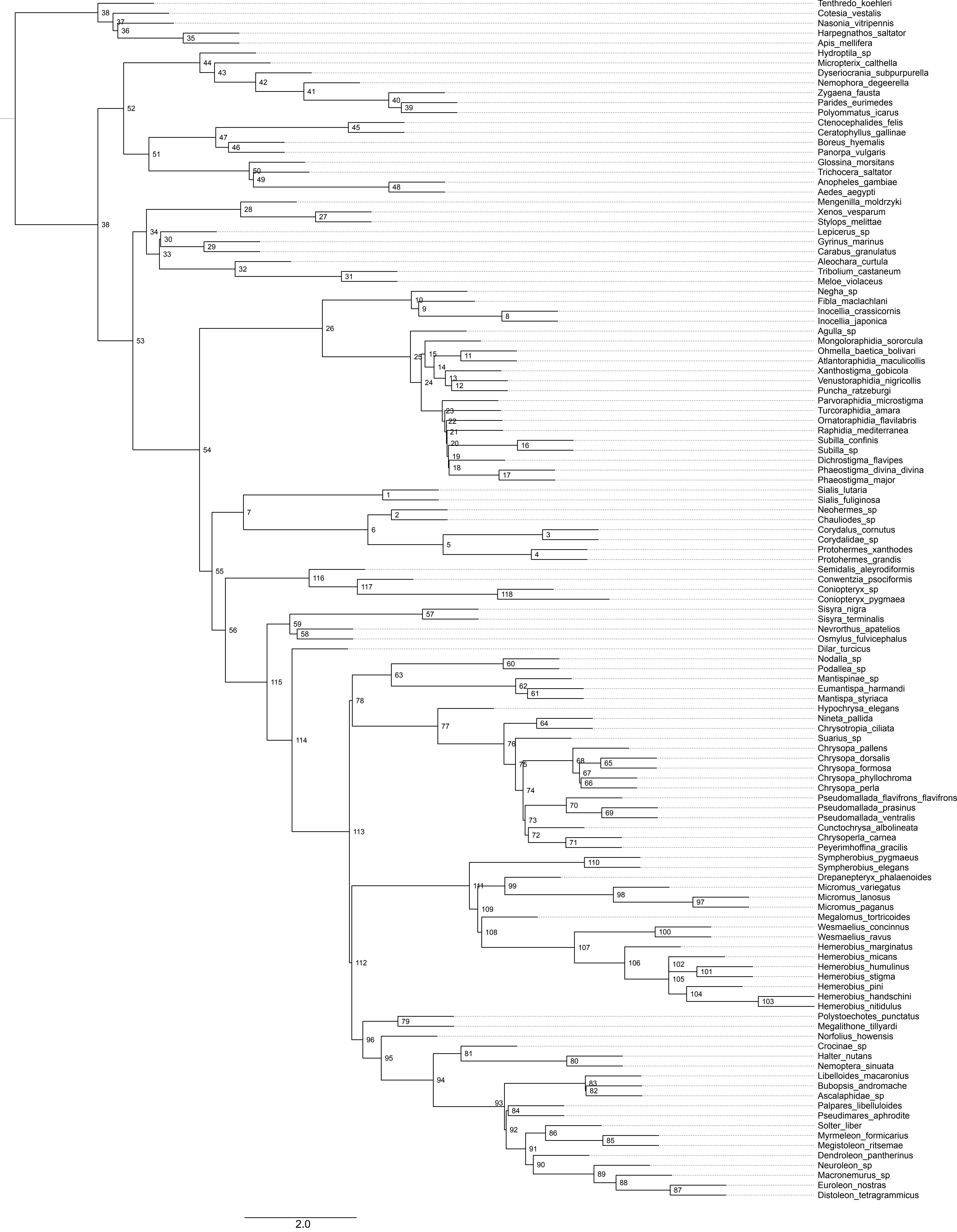

Figure S15: Phylogenetic tree that resulted from the species-tree analysis with ASTRAL when analyzing the gene trees of the amino-acid supermatrix G (see Fig. 2a). Values on branches correspond to the branch numbers in Table S16. All the different coalescent-based branch statistics are given in Table S16.

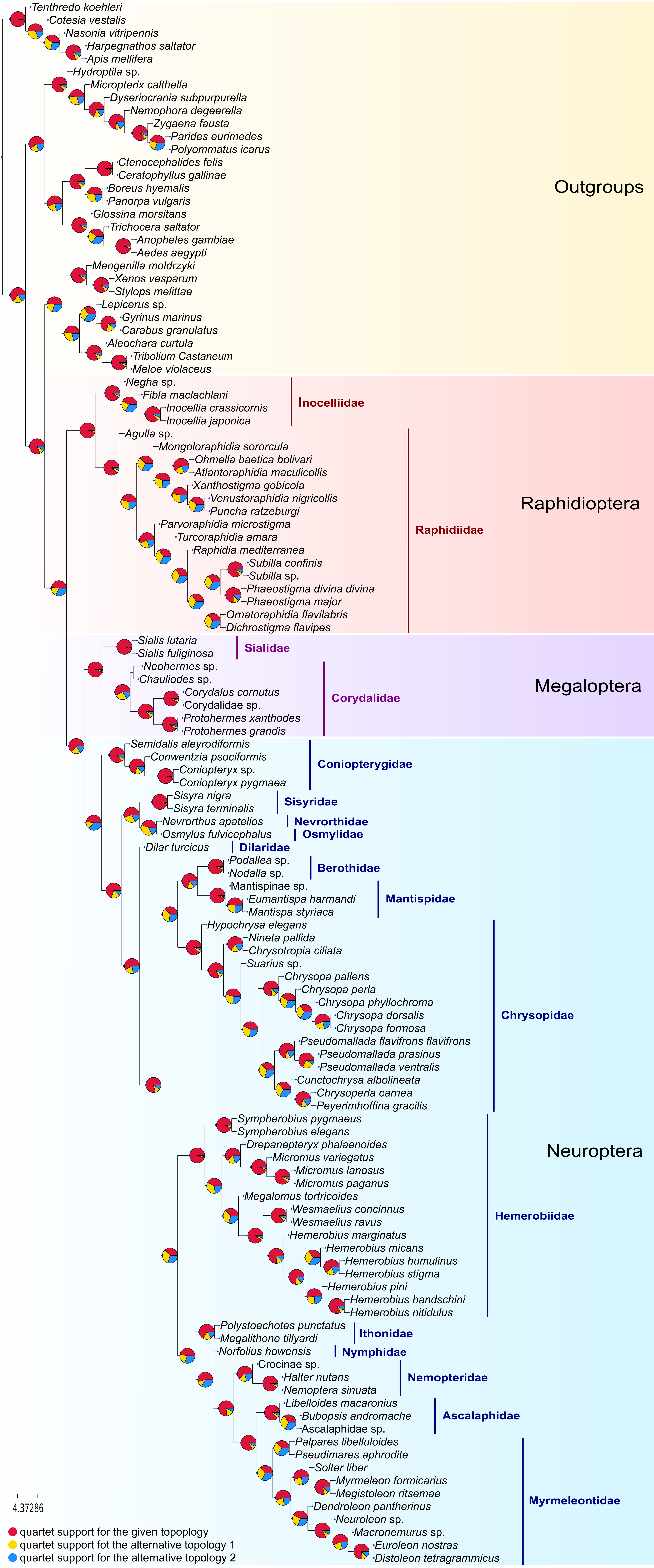

Figure S16: Phylogenetic tree that resulted from the species tree analysis with ASTRAL when analyzing the gene trees of the amino-acid supermatrix H. Pie charts on branches show quartet support (q1, q2, q3) calculated with ASTRAL.

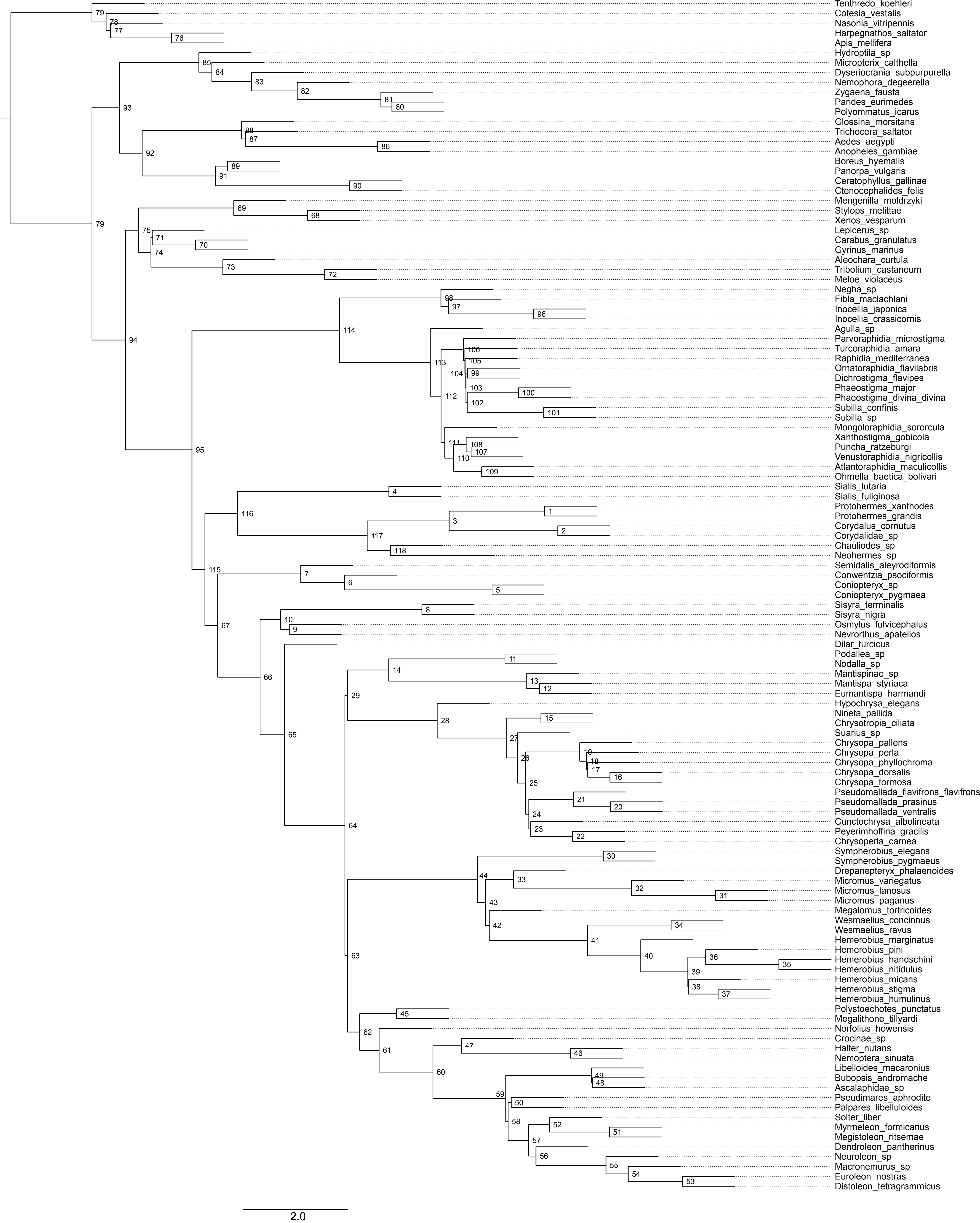

Figure S17: Phylogenetic tree that resulted from the species tree analysis with ASTRAL when analyzing the gene trees of the amino-acid supermatrix H. Values on branches correspond to the branch numbers in Table S18. All the different coalescent-based branch statistitics are given in Table S18.

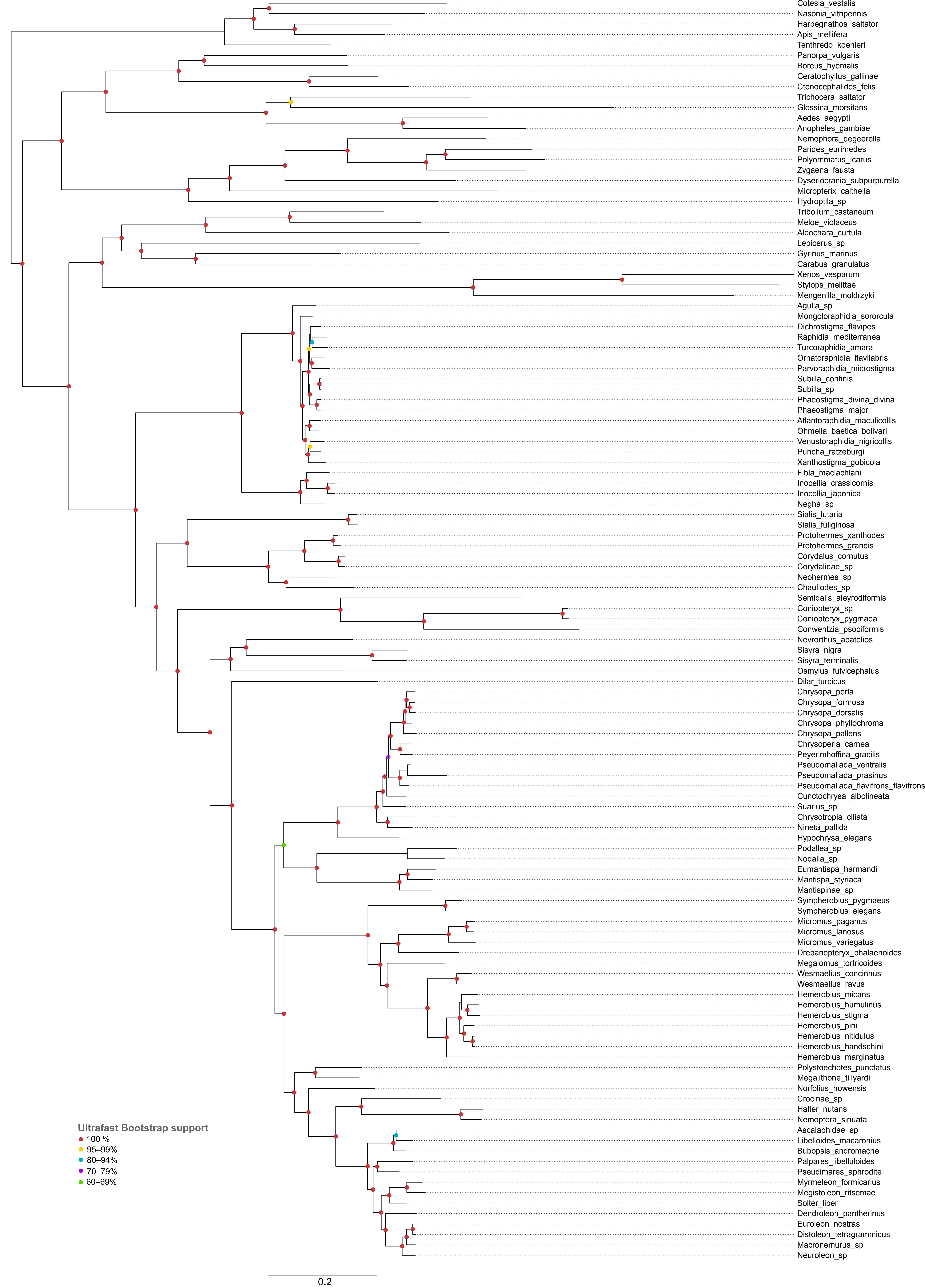

Figure S18: Phylogenetic tree with the highest log-likelihood score that resulted from the partitioned concatenated analysis of the amino-acid supermatrix G when calculating UFB support. Colored circles indicate UFB support based on 1,000 replicates.

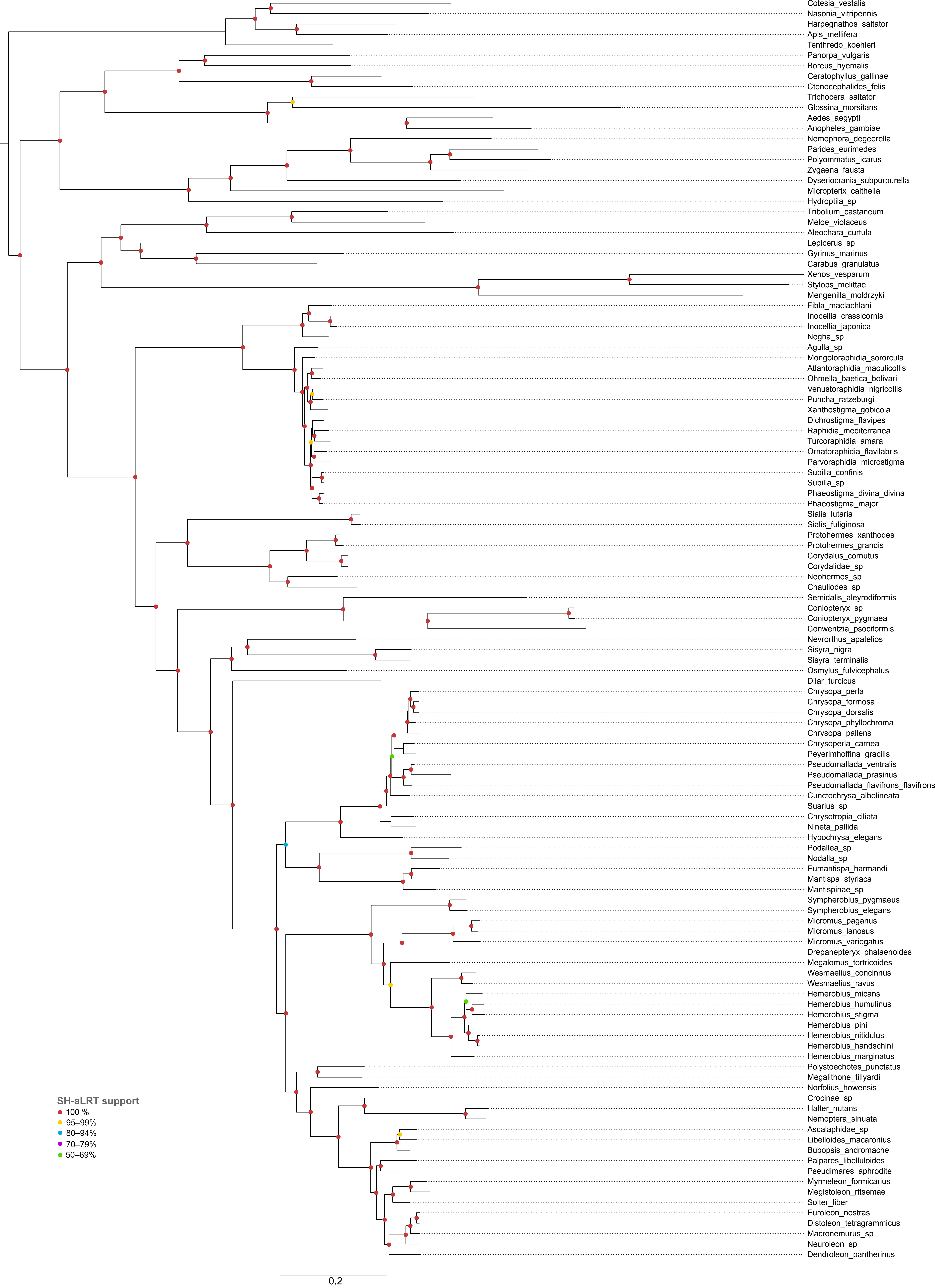

Figure S19: Phylogenetic tree with the highest log-likelihood score that resulted from the partitioned concatenated analysis of the amino-acid supermatrix G. Colored circles indicate branch support based on 10,000 SH-aLRT replicates.

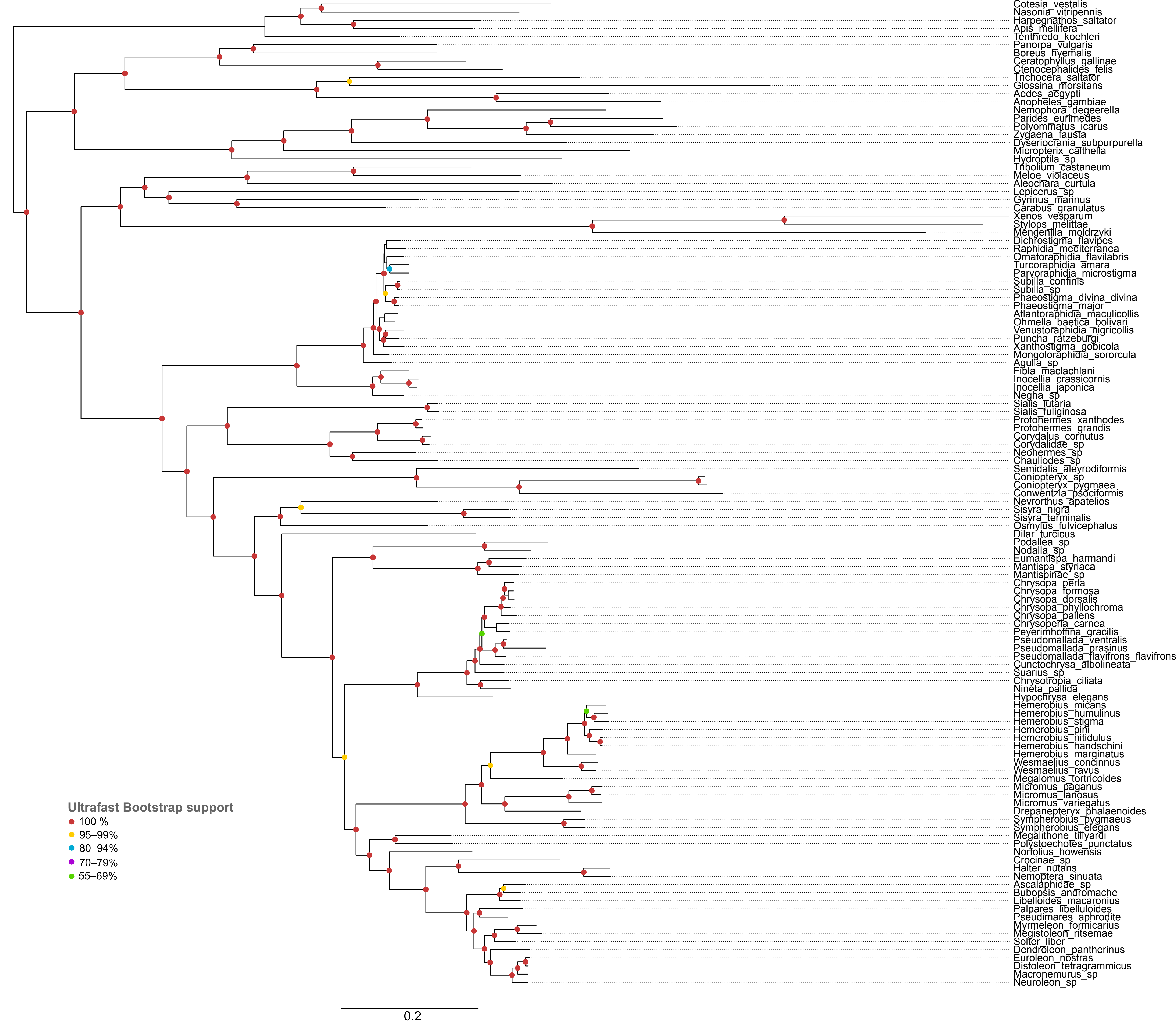

Figure S20: Phylogenetic tree with the highest log-likelihood score that resulted from the partitioned concatenated analysis of the amino-acid supermatrix H when calculating UFB support. Colored circles indicate UFB support based on 1,000 replicates.

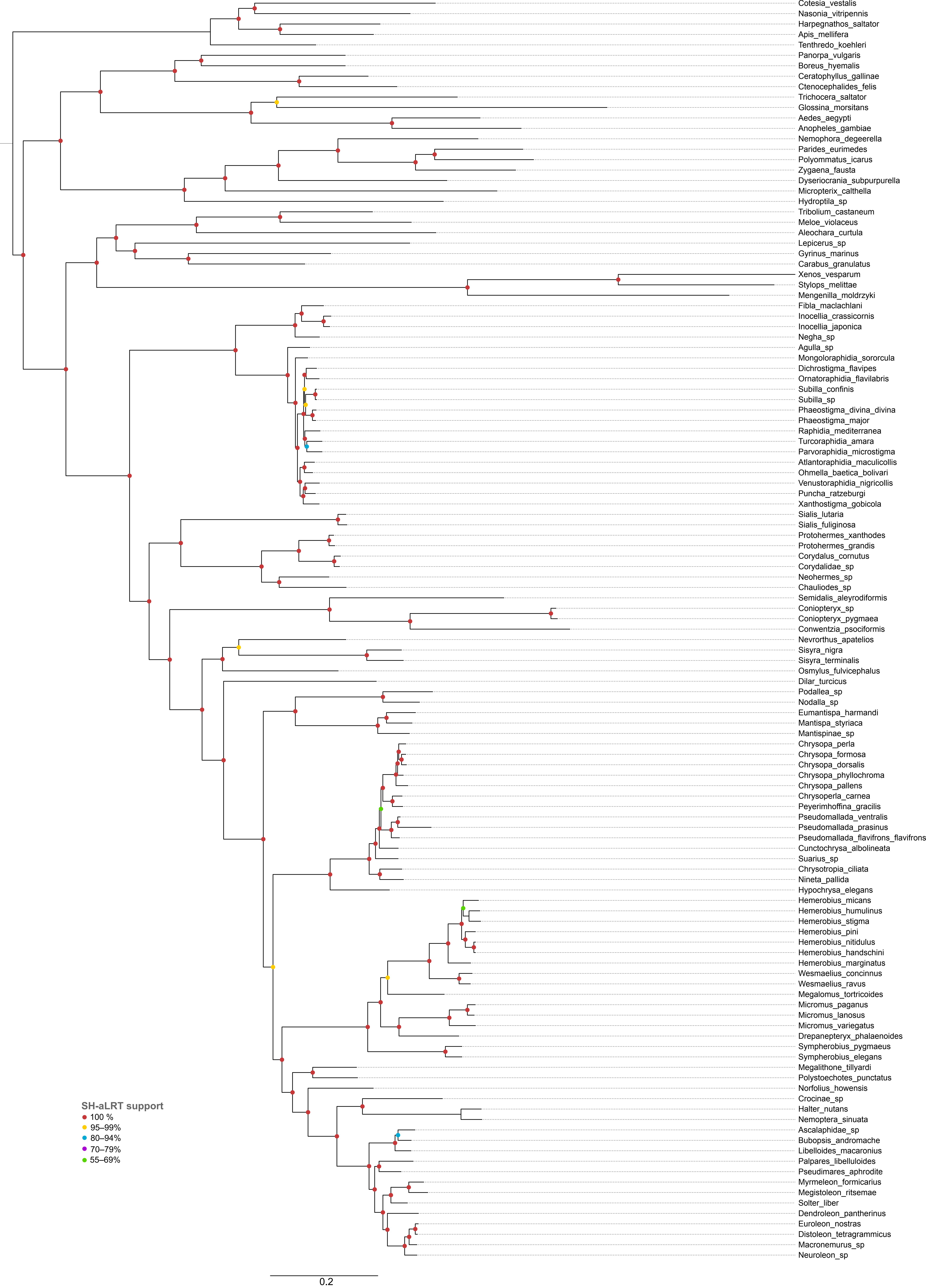

Figure S21: Phylogenetic tree with the highest log-likelihood score that resulted from the partitioned concatenated analysis of the amino-acid supermatrix H. Colored circles indicate branch support based on 10,000 SH-aLRT replicates.

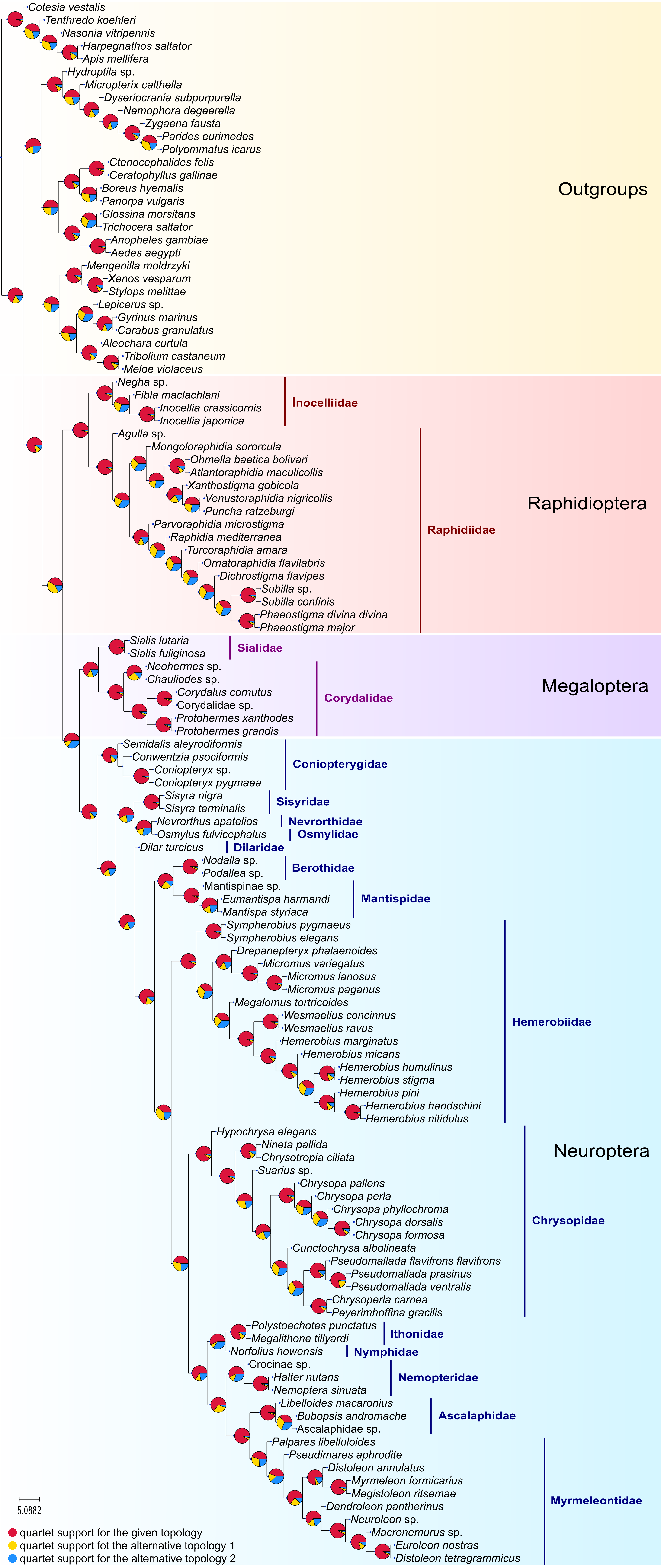

Figure S22: Phylogenetic tree that resulted from the species tree analysis with ASTRAL when analyzing the gene trees of the nucleotide supermatrix G-nt. Pie charts on branches show quartet support (q1, q2, q3) calculated with ASTRAL.

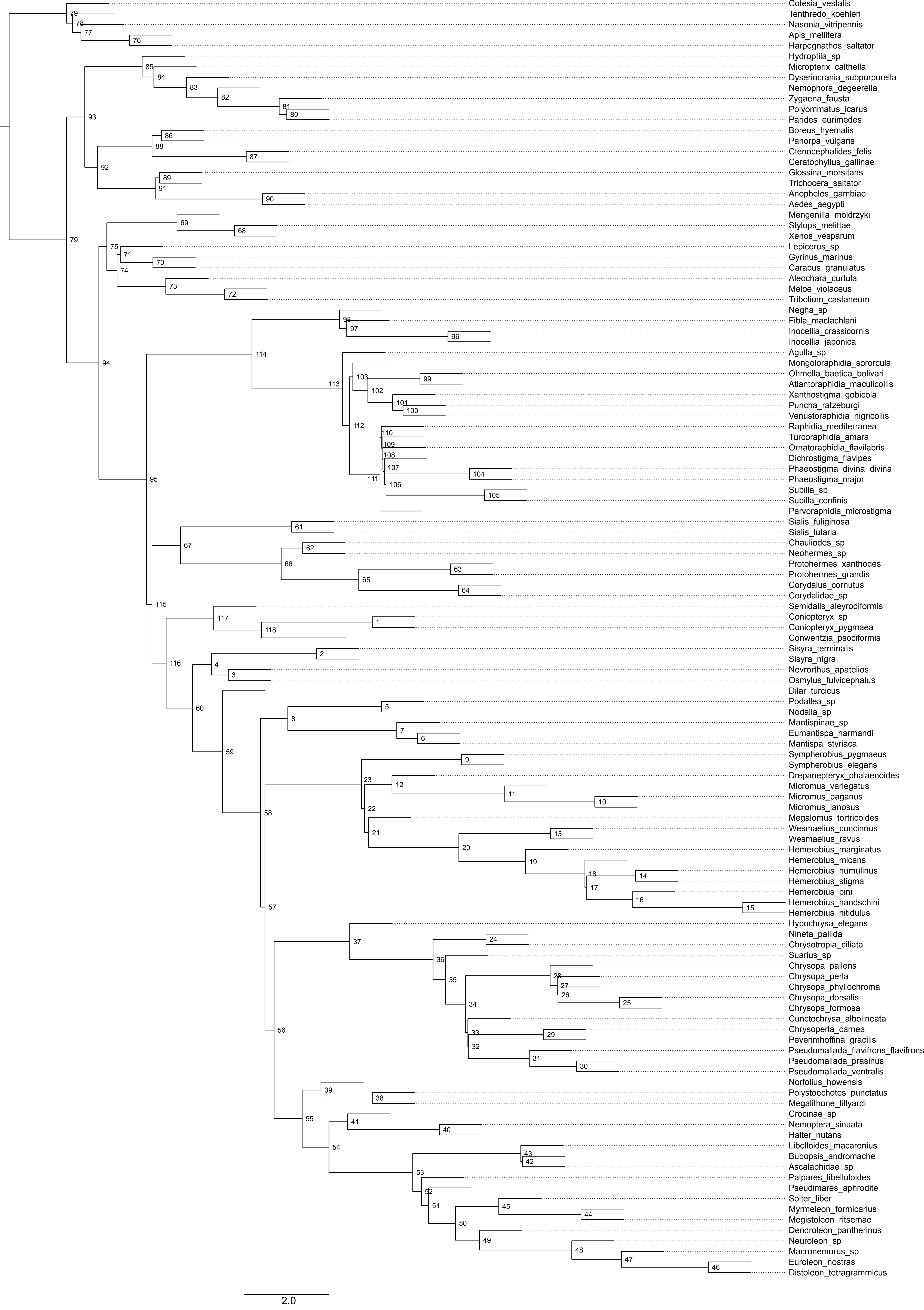

Figure S23: Phylogenetic tree that resulted from the species tree analysis with ASTRAL when analyzing the gene trees of the nucleotide supermatrix G-nt. Values on branches correspond to the branch numbers in Table S17. All the different coalescent-based branch statistitics are given in Table S17.

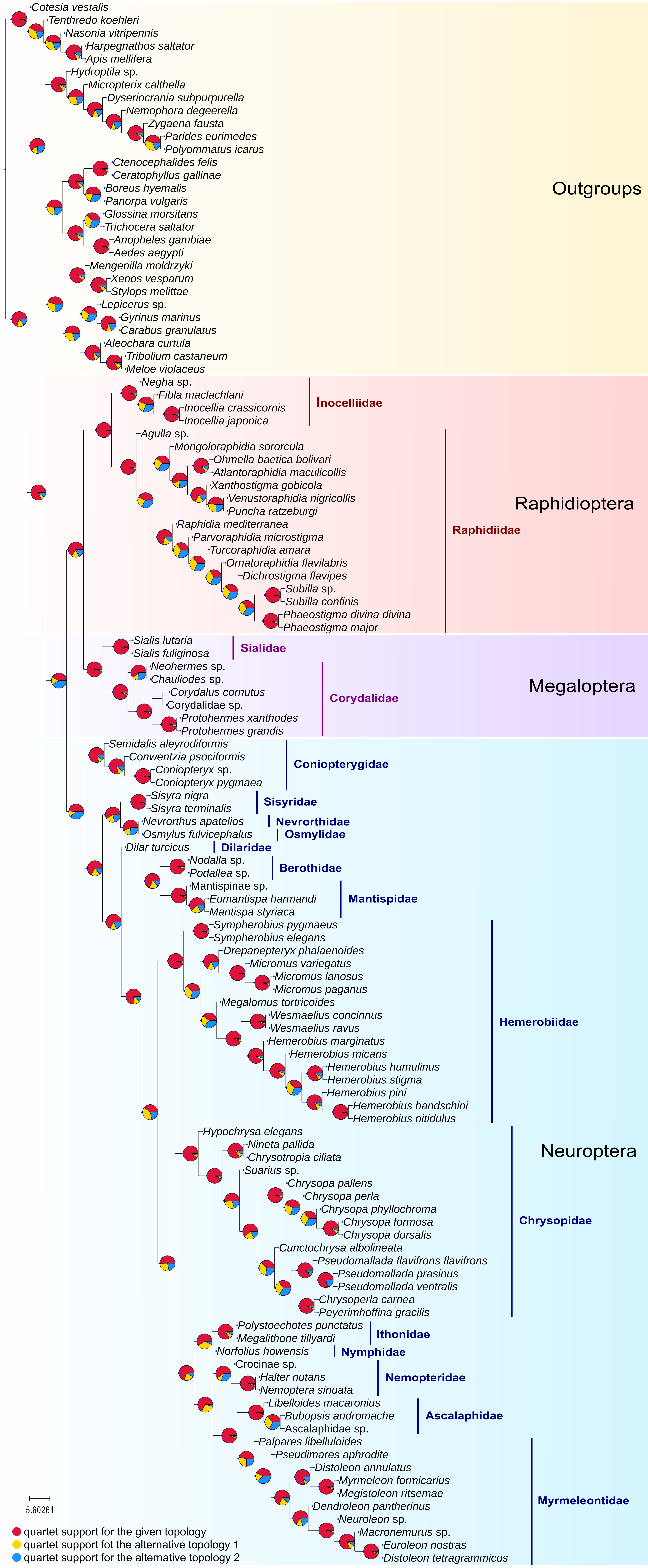

Figure S24: Phylogenetic tree that resulted from the species tree analysis with ASTRAL when analyzing the gene trees of the nucleotide supermatrix H-nt. Pie charts on branches show quartet support (q1, q2, q3) calculated with ASTRAL.

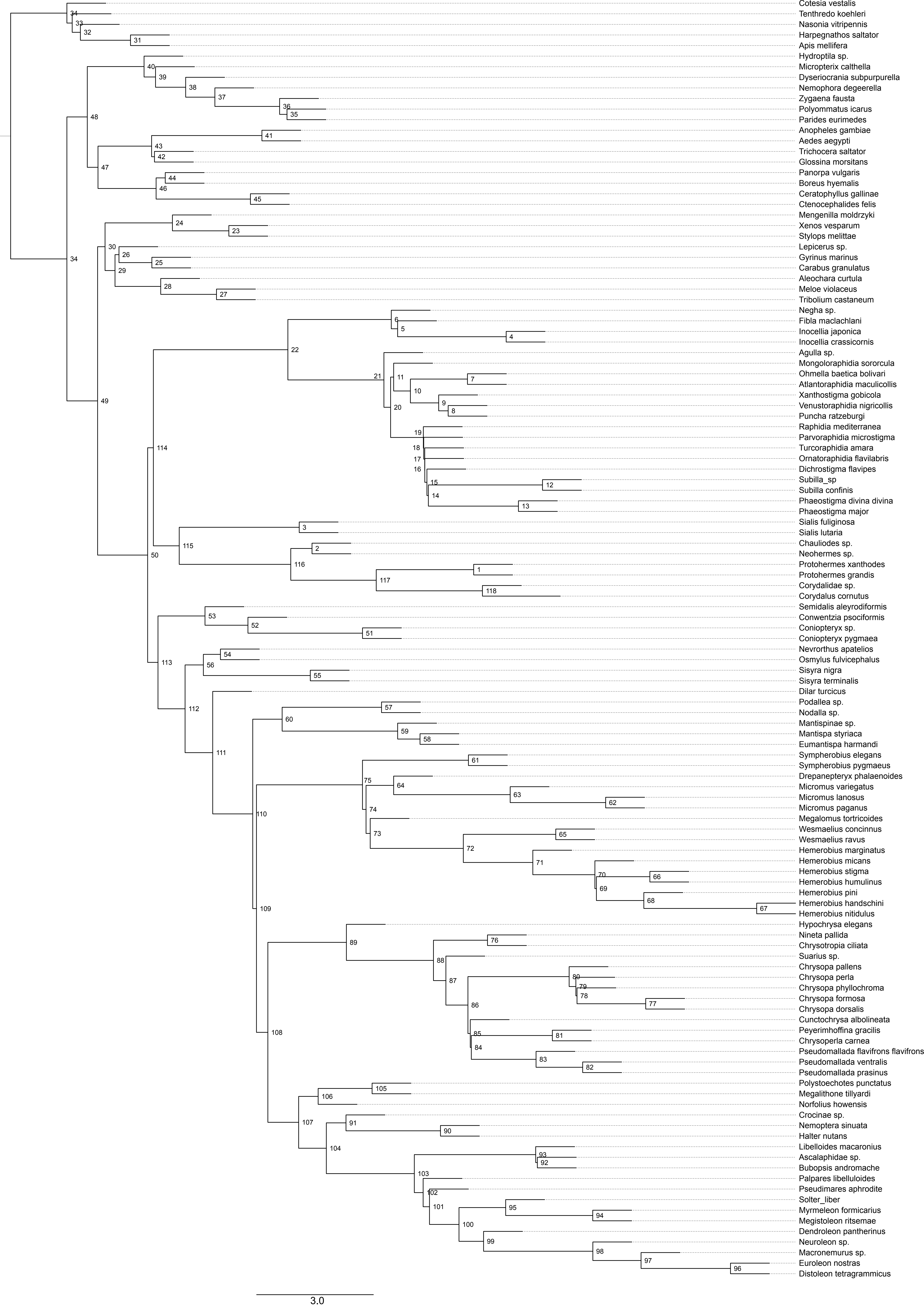

Figure S25: Phylogenetic tree that resulted from the species tree analysis with ASTRAL when analyzing the gene trees of the amino-acid supermatrix H-nt. Values on branches correspond to the branch numbers in Table S19. All the different coalescent-based branch statistics are given in Table S19.

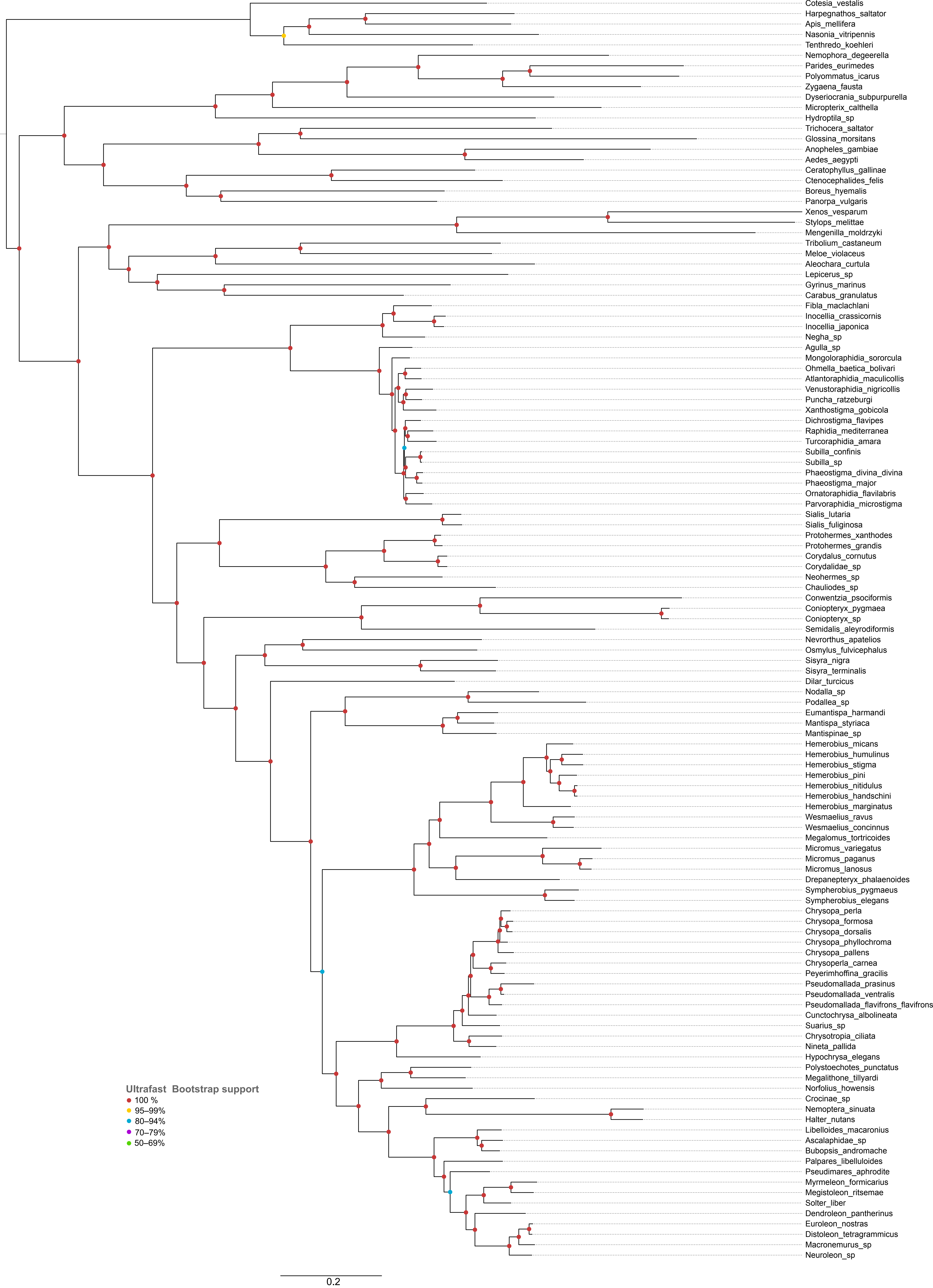

Figure S26: Phylogenetic tree with the highest log-likelihood score that resulted from the partitioned concatenated analysis of the nucleotide supermatrix G-nt when calculating UFB support. Colored circles indicate UFB support based on 1,000 replicates.

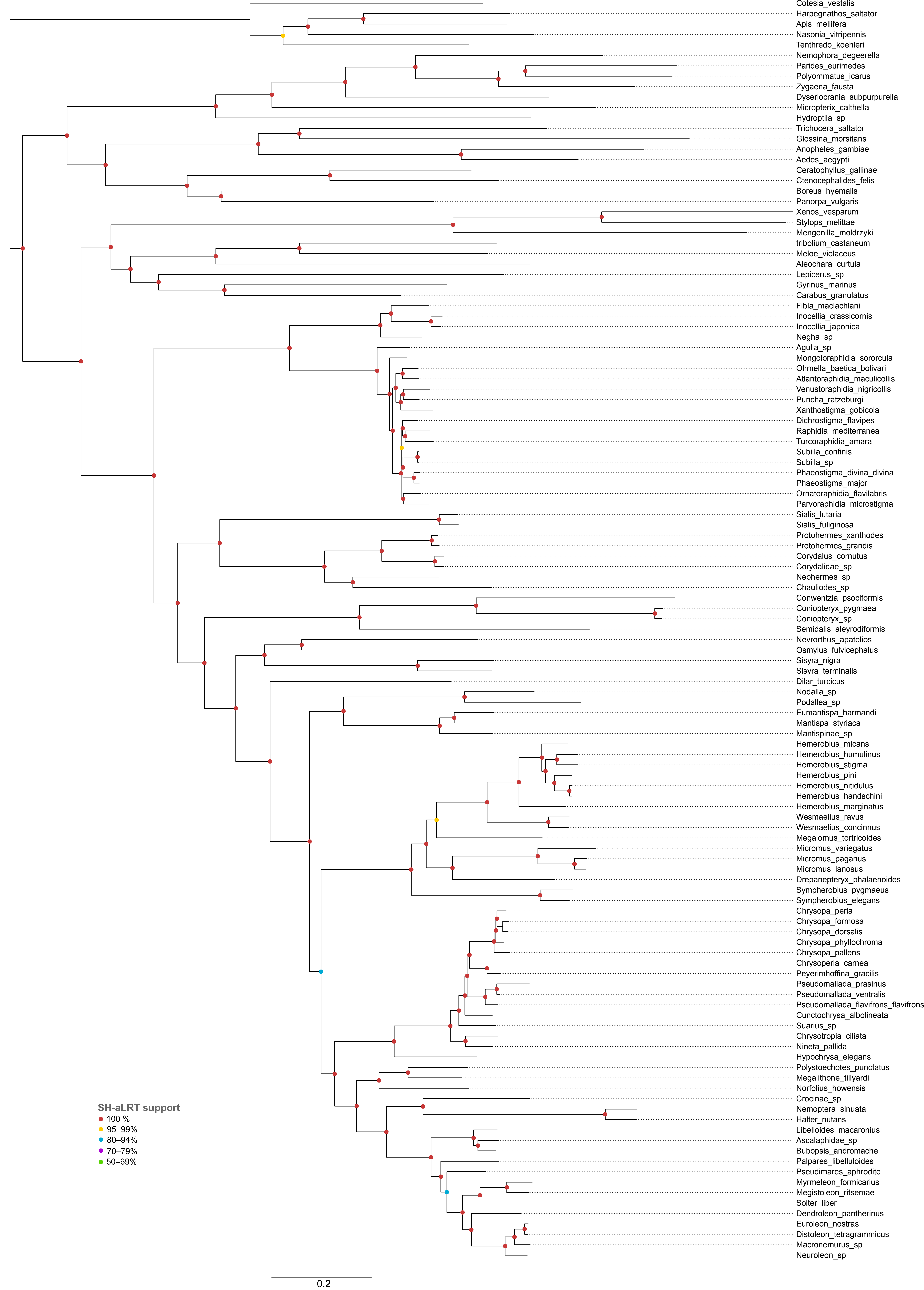

Figure S27: Phylogenetic tree with the highest log-likelihood score that resulted from the partitioned concatenated analysis of the nucleotide supermatrix G-nt. Colored circles indicate branch support based on 10,000 SH-aLRT replicates.

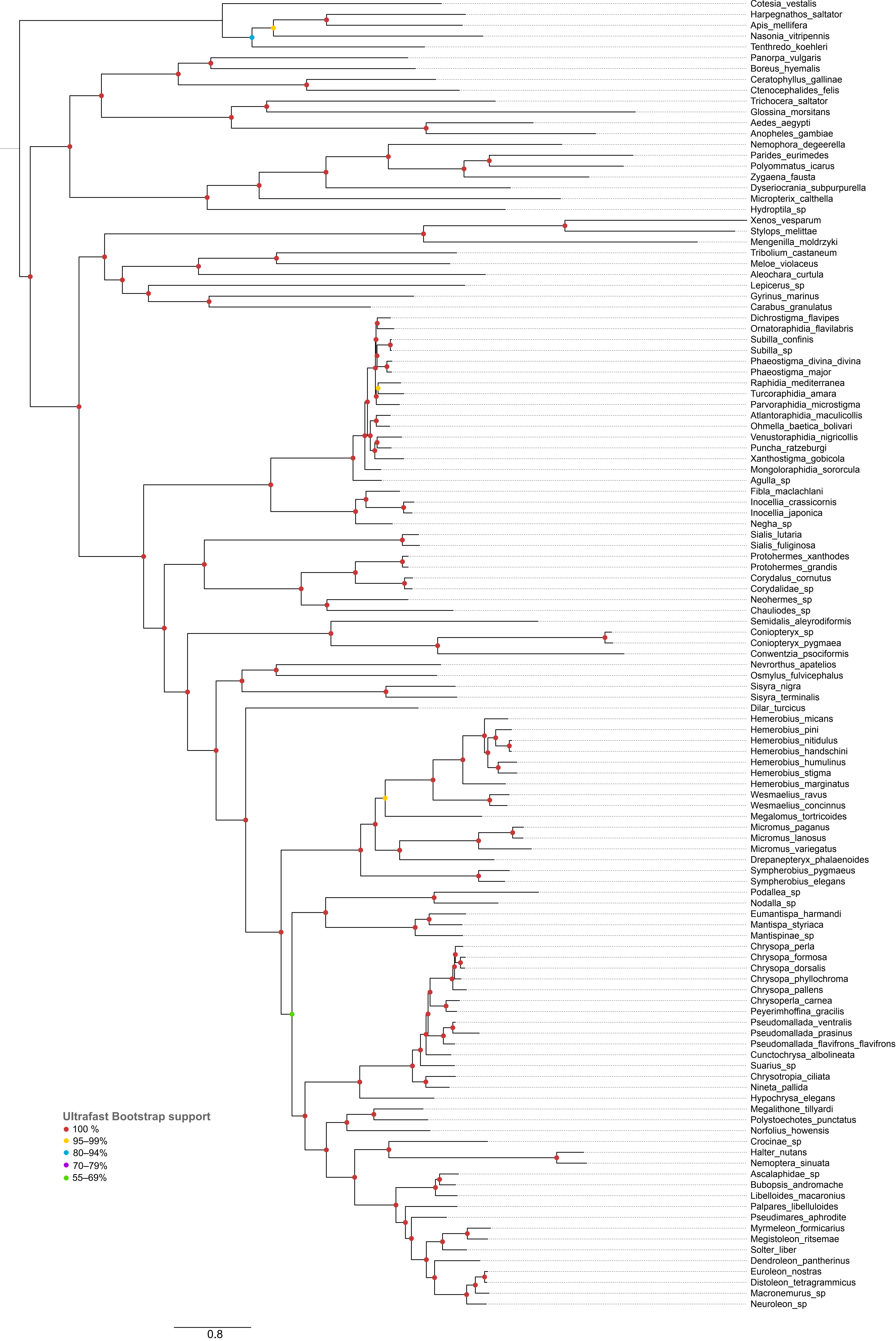

Figure S28: Phylogenetic tree with the highest log-likelihood score that resulted from the partitioned concatenated analysis of the nucleotide supermatrix H-nt when calculating UFB support. Colored circles indicate UFB support based on 1,000 replicates.

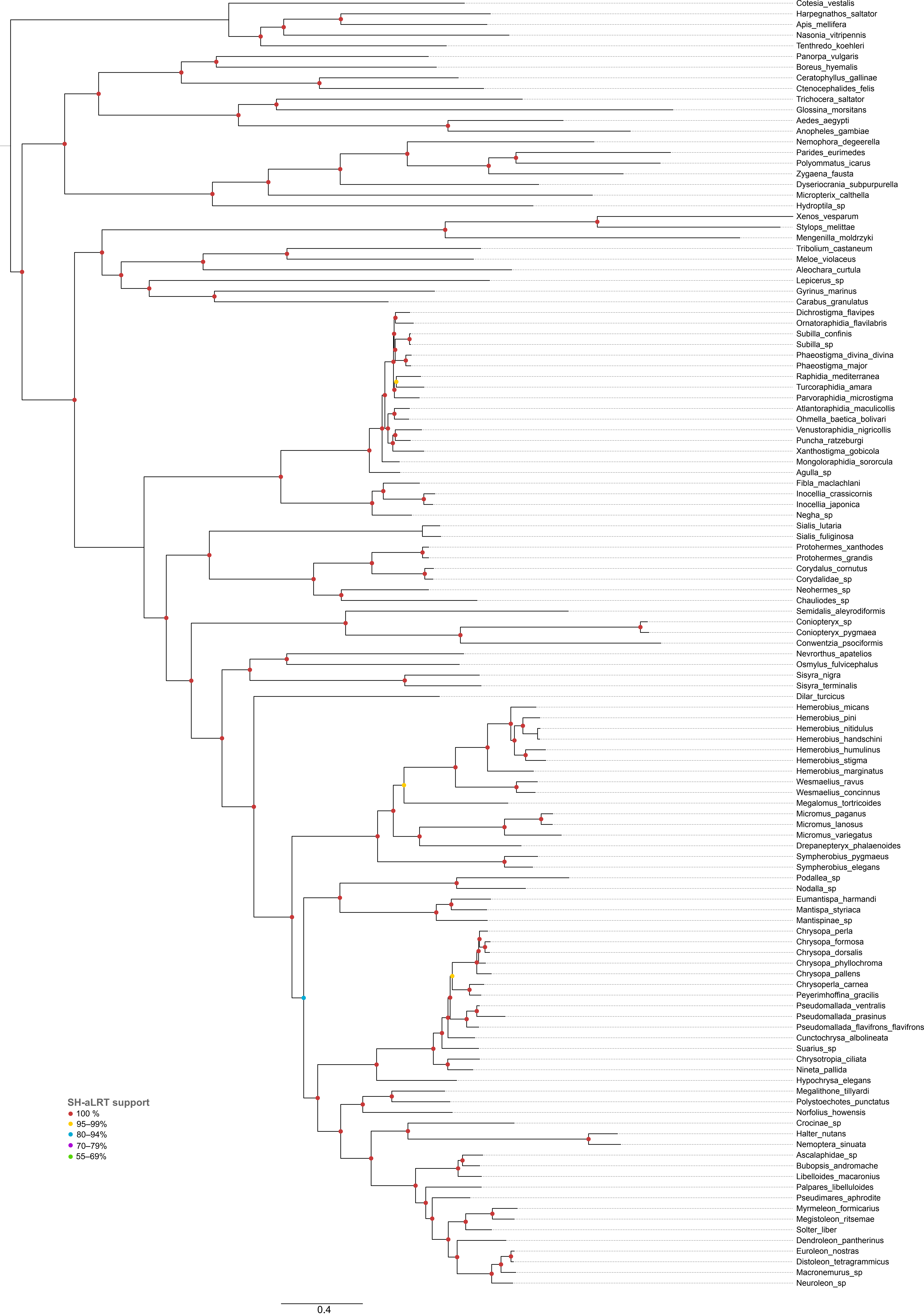

Figure S29: Phylogenetic tree with the highest log-likelihood score that resulted from the partitioned concatenated analysis of the nucleotide supermatrix H-nt . Colored circles indicate branch support based on 10,000 SH-aLRT replicates.

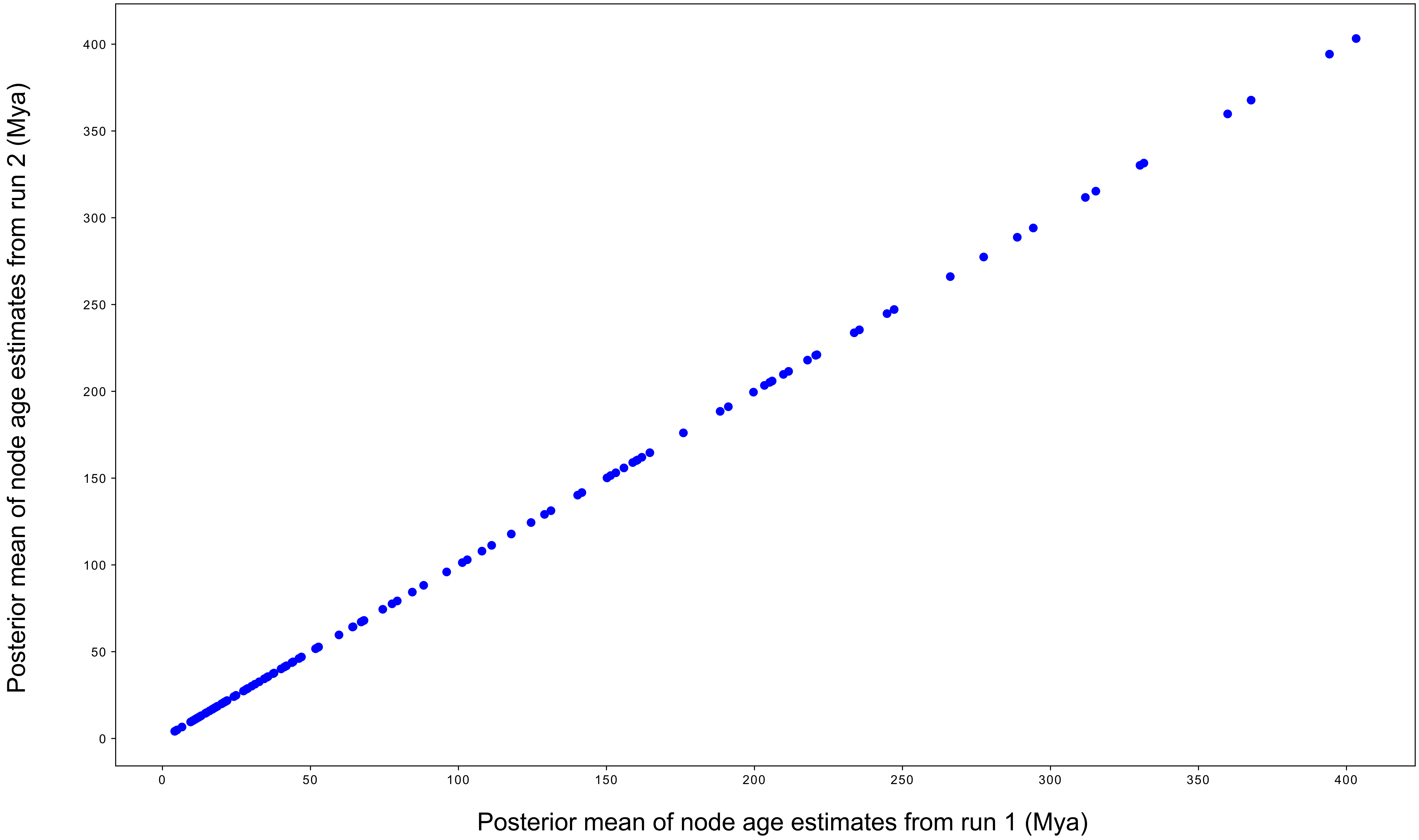

Figure S30: Scatter plot of the posterior mean node age estimates from run 1 plotted against the posterior mean node age estimates from run 2 when including the full set of fossil calibrations.

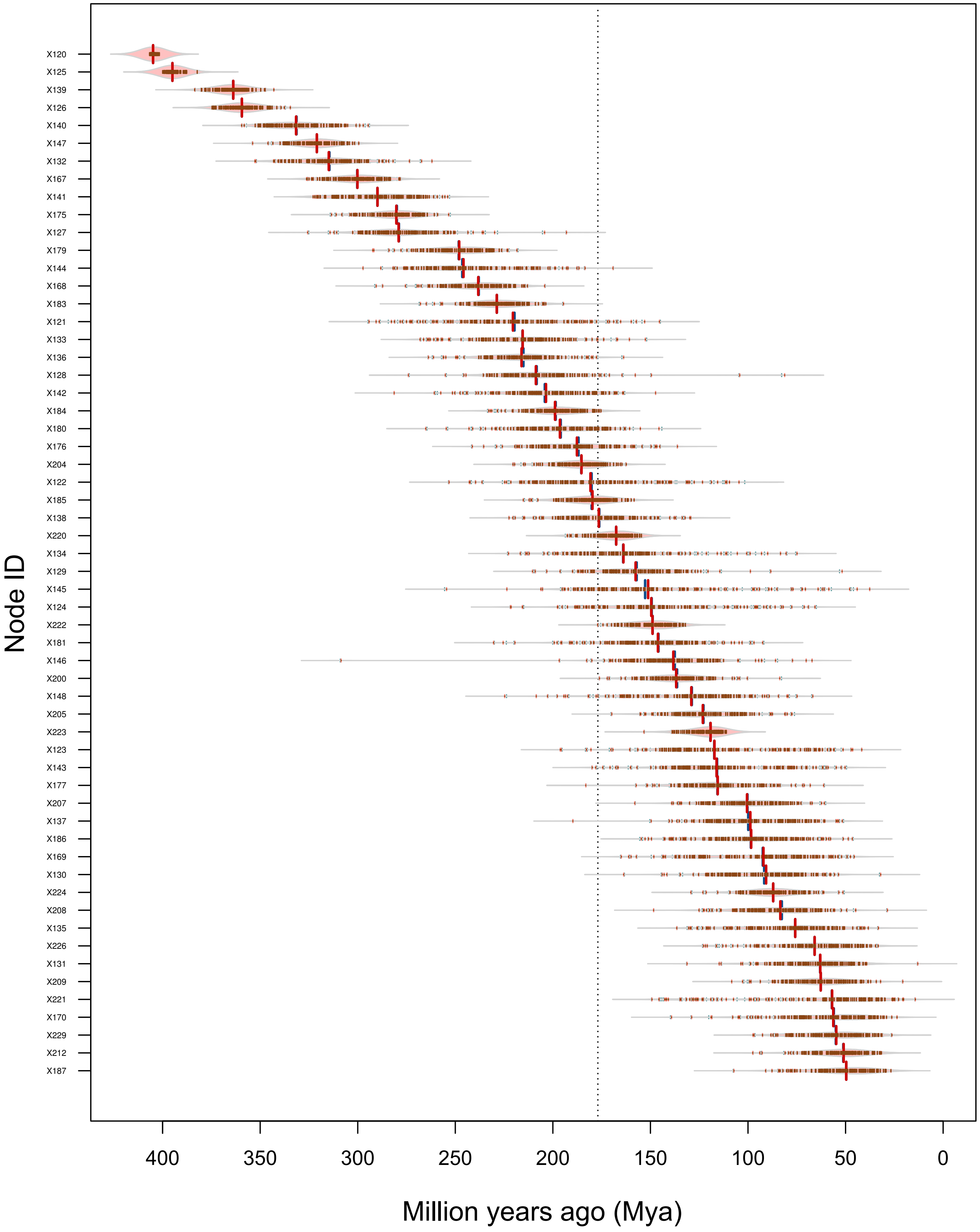

Figure S31: Beanplots of posterior median node age estimates of each meta-partition from run 1 and run 2 when using all fossil calibration points. The medians of posterior ages from run 2 are ploted in red whereas the medians of posterior ages from run 1 are plotted in blue. Median node age estimates from run 2 are almost completely overlapping with the medians from run 1.

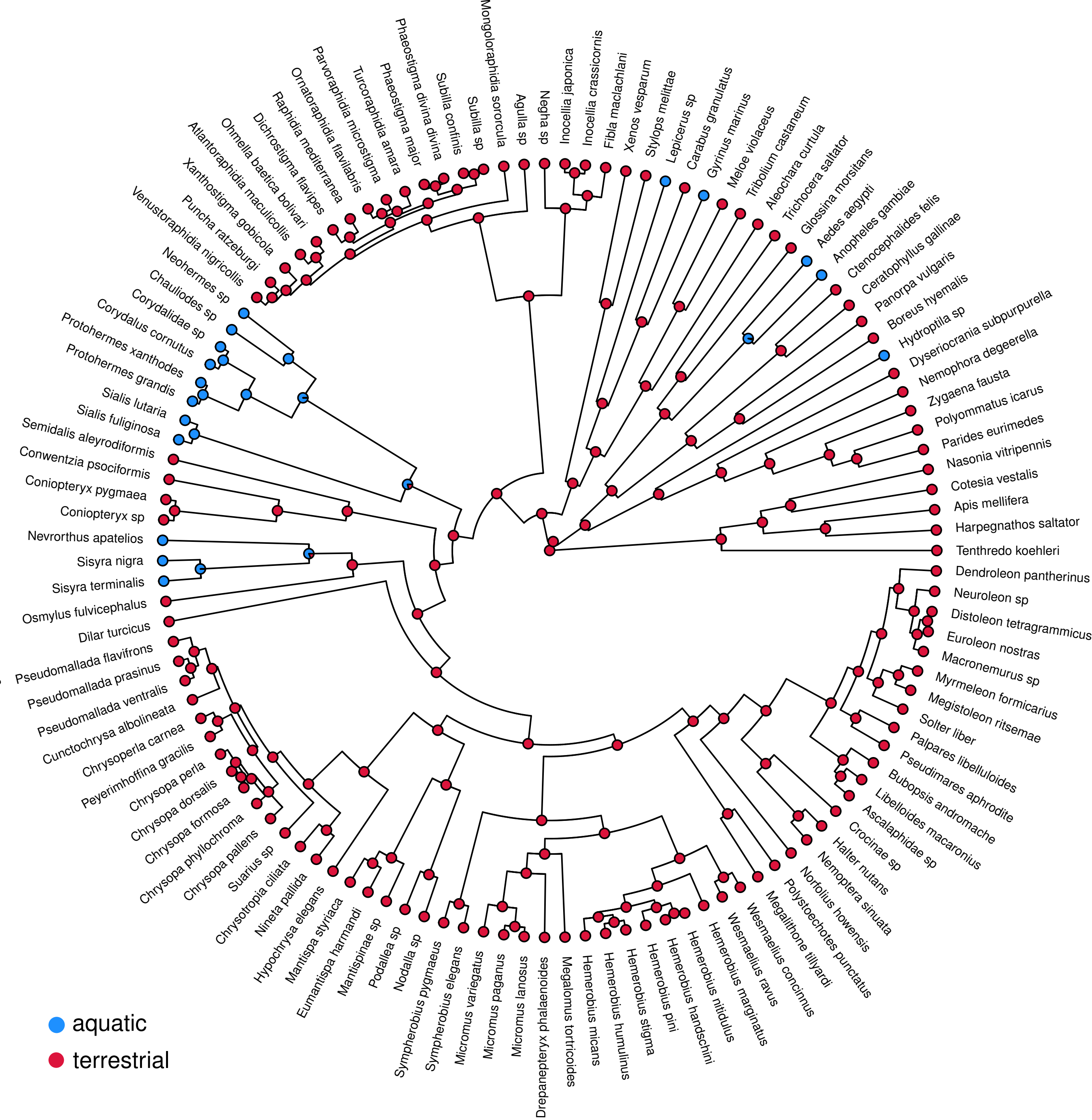

Figure S32: Summarized results of 10,000 stochastic character maps under the ARD model for the ultrametric tree of Fig. 1 (note that the larva of *Lepicerus* sp. was coded as aquatic in contrast to Fig. 4). Colored circles at the tips show the coded state for each species. Pie charts on internal tree nodes show posterior probabilities of states at the nodes.

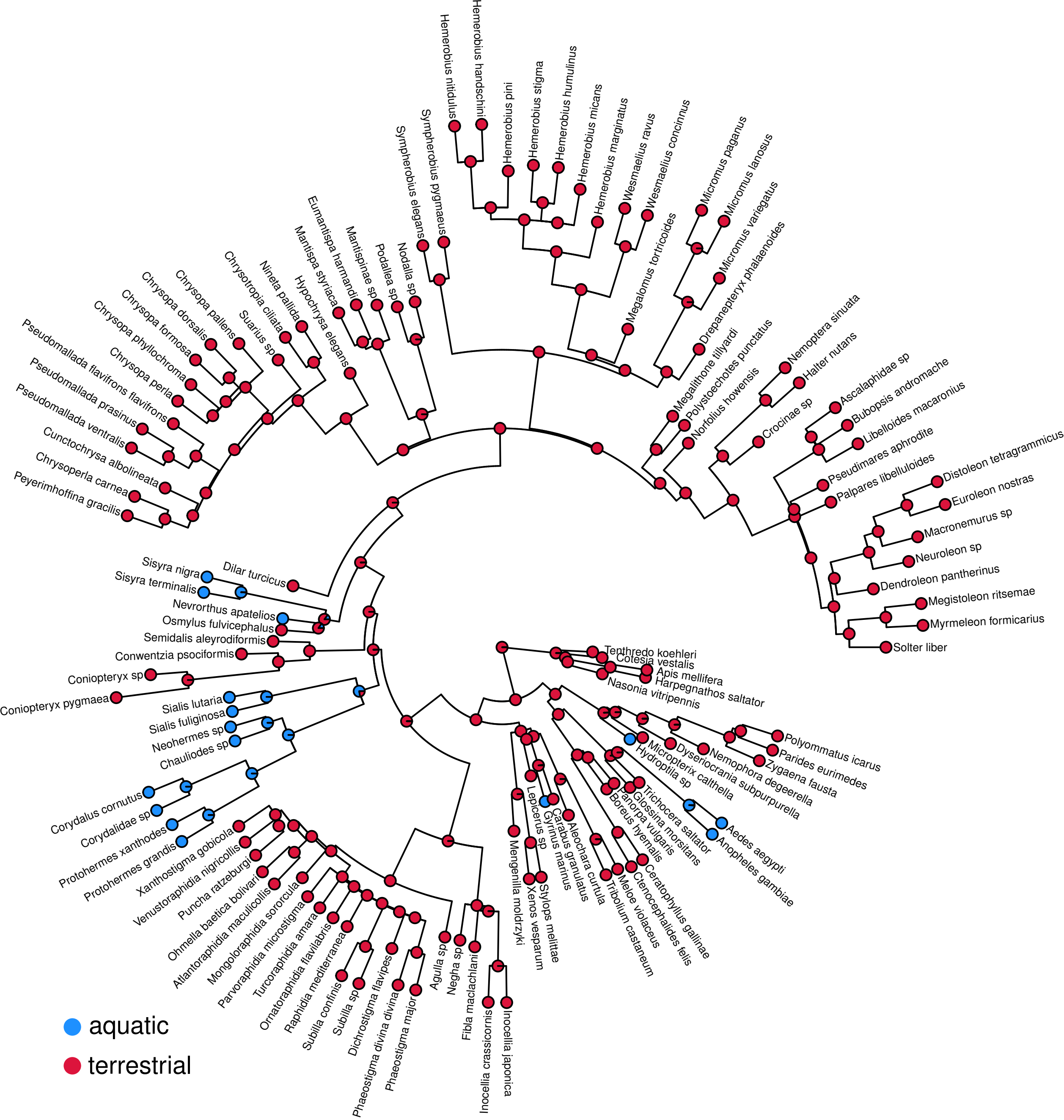

Figure S33: Summarized results of 10,000 stochastic character maps under the ER model for the topology and branch lenghts of the coalescent-based species tree of Fig. 2a (note that the larva of *Lepicerus* sp. was coded as terrestrial). Colored circles at the tips show the coded state for each species. Pie charts on internal tree nodes show posterior probabilities of states at the nodes.

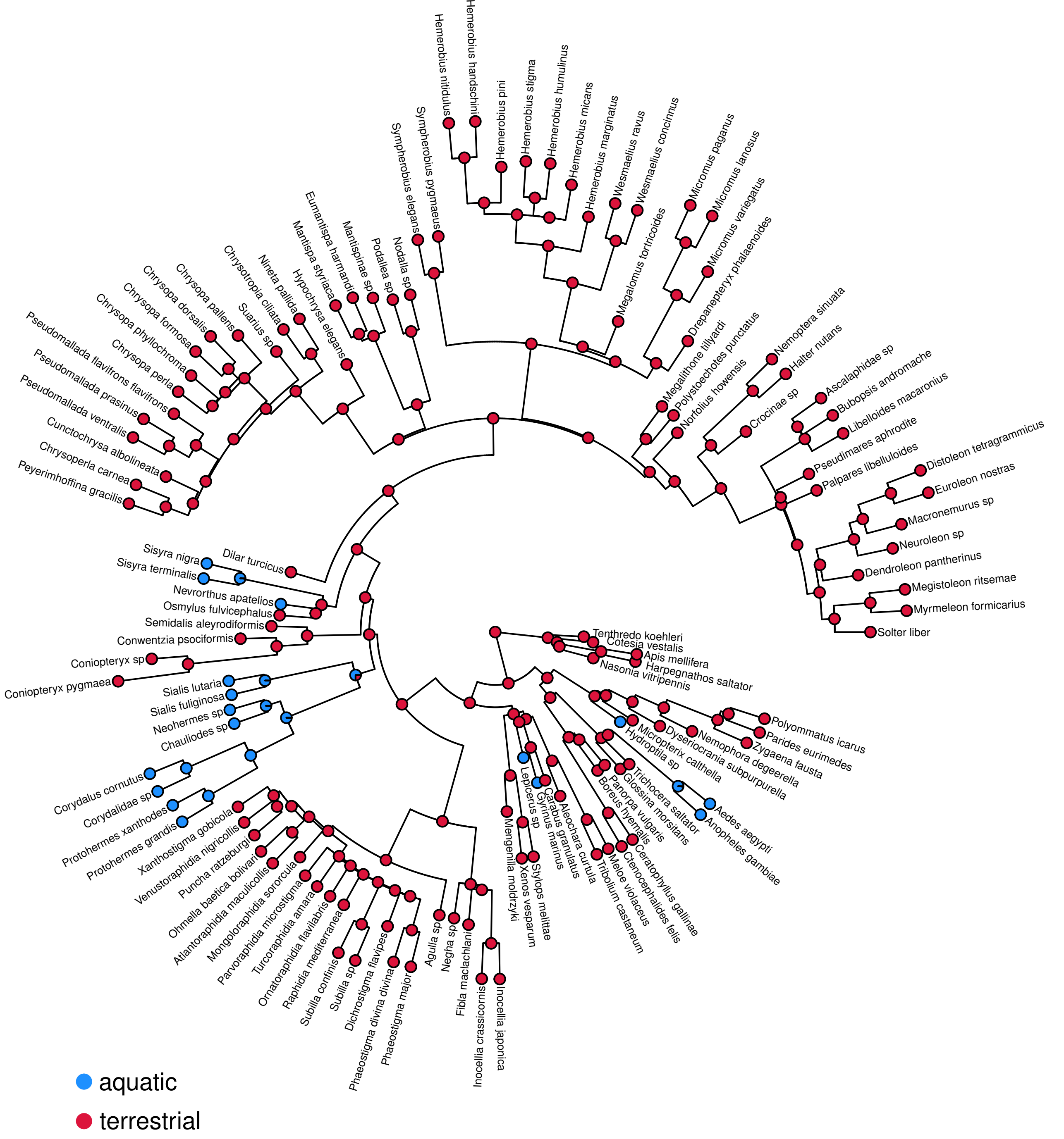

Figure S34: Summarized results of 10,000 stochastic character maps under the ARD model for the coalescent-based species tree of Fig. 2a (note that the larva of *Lepicerus* sp. was coded as aquatic). Colored circles at the tips show the coded state for each species. Pie charts on internal tree nodes show posterior probabilities of states at the nodes.

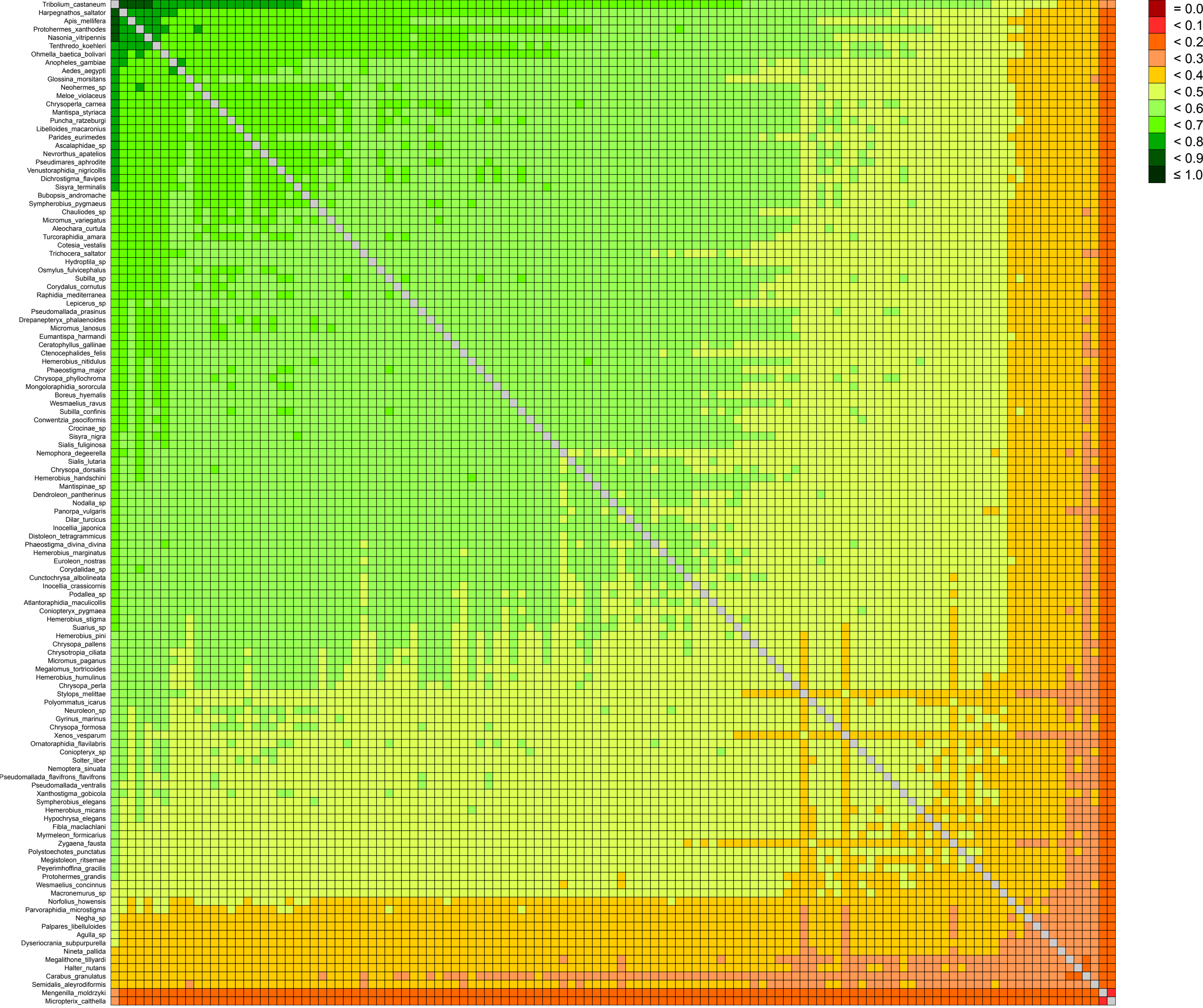

Figure S35: Rectangular Alistat heatmap showing pairwise alignment completeness scores for all species included in supermatrix A. Values closer to 1 indicate higher completeness scores for the pairwise sequence comparisons.

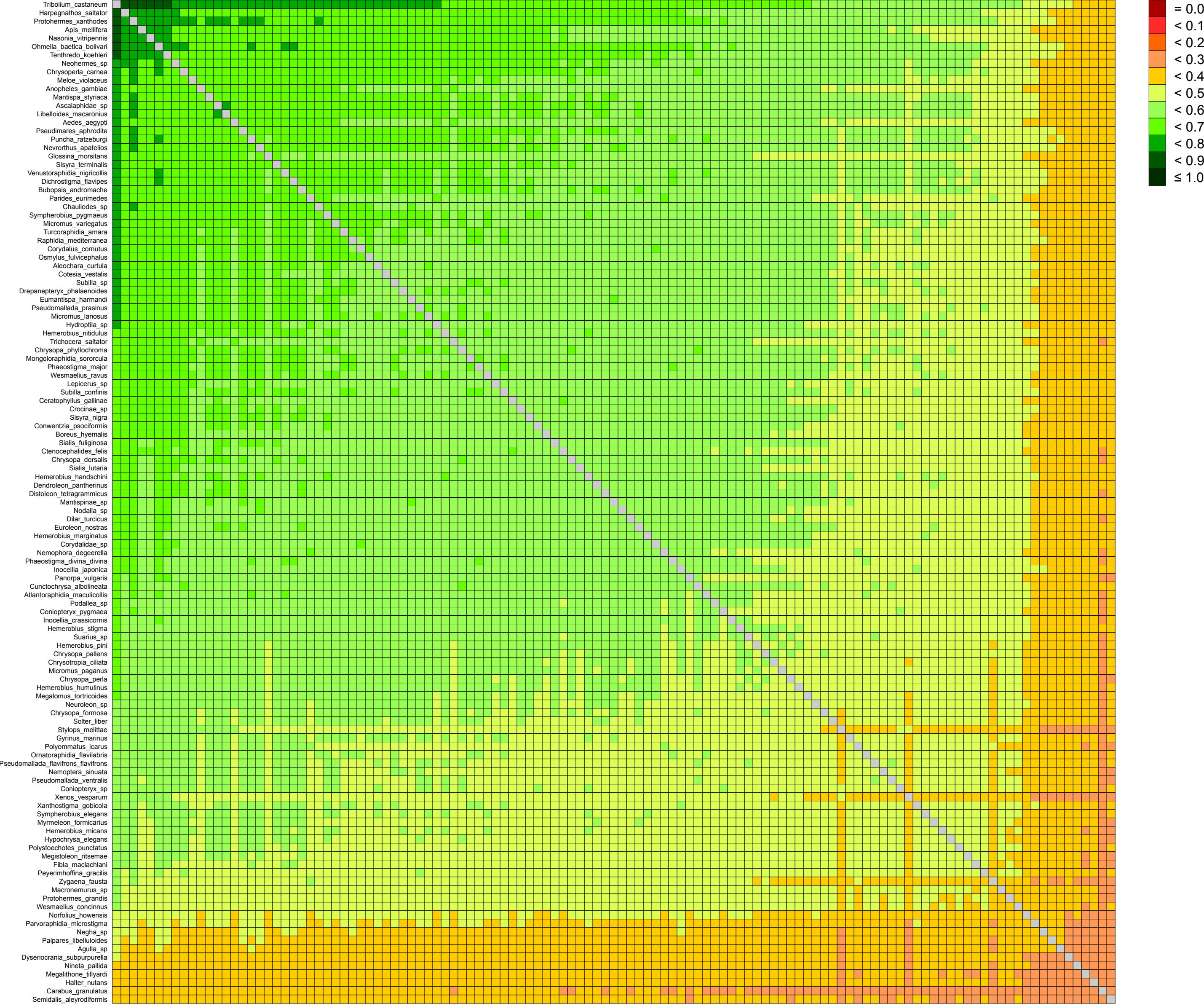

Figure S36: Rectangular Alistat heatmap showing pairwise alignment completeness scores for all species included in supermatrix B. Values closer to 1 indicate higher completeness scores for the pairwise sequence comparisons.

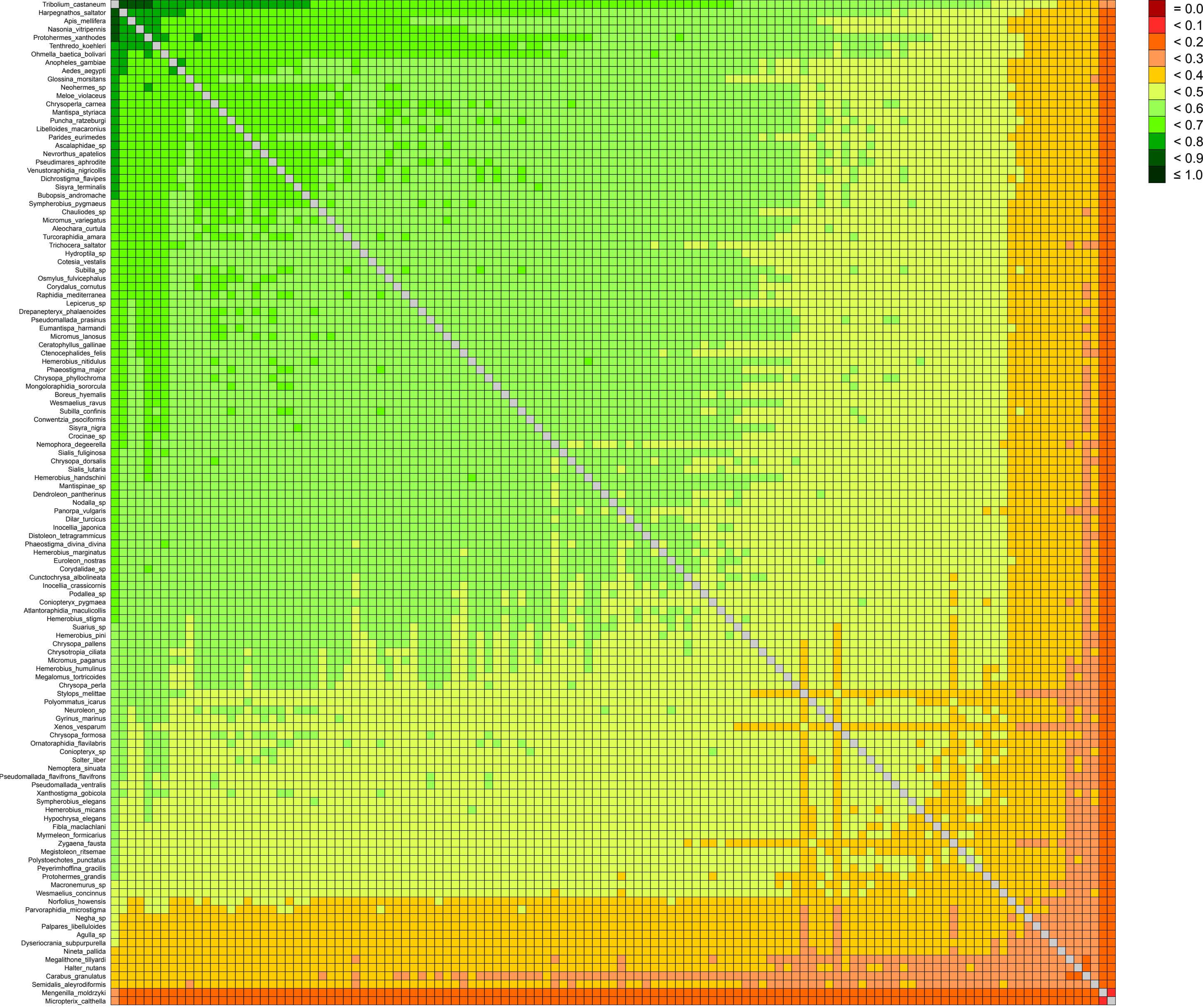

Figure S37: Rectangular Alistat heatmap showing pairwise alignment completeness scores for all species included in supermatrix C. Values closer to 1 indicate higher completeness scores for the pairwise sequence comparisons.

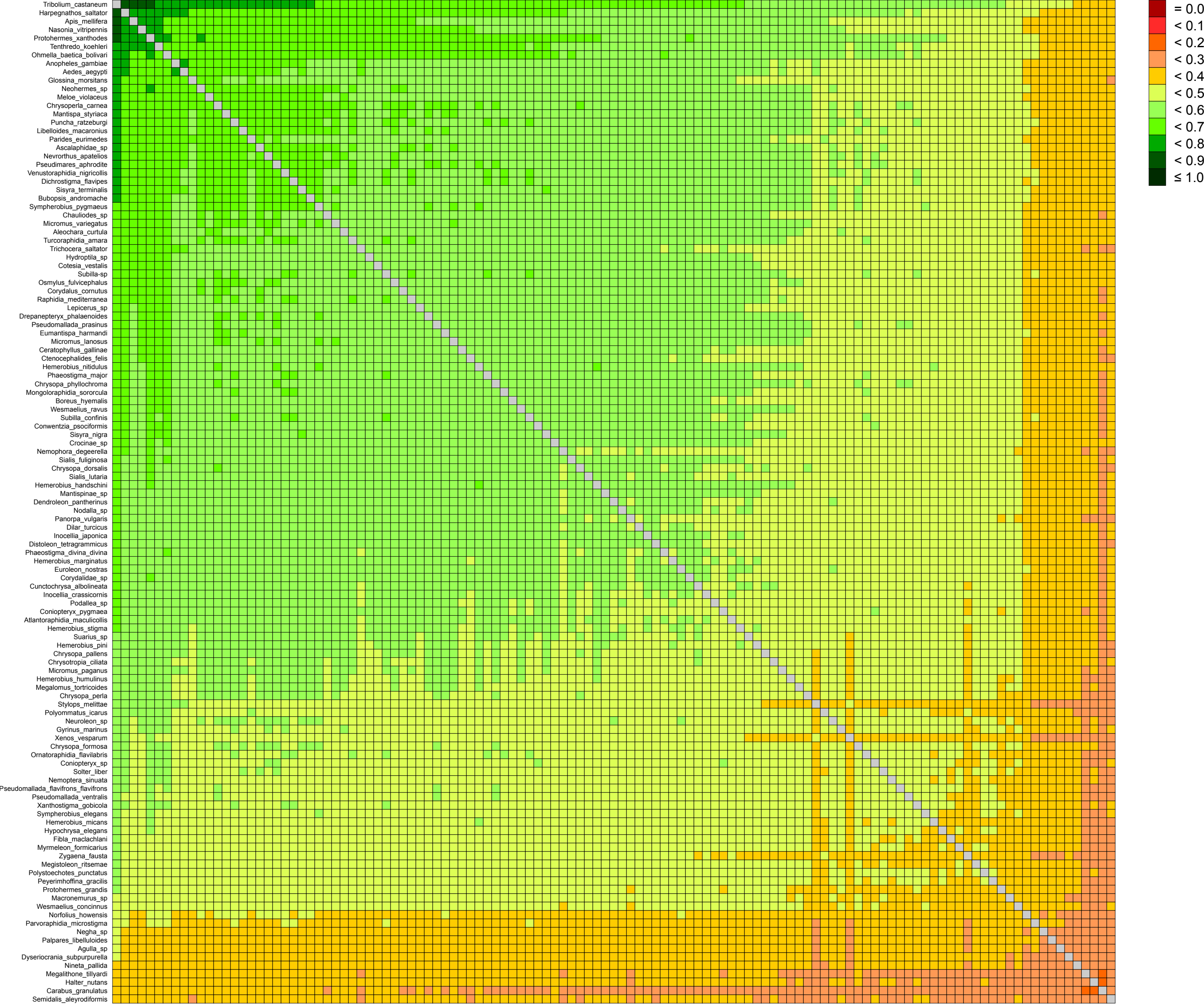

Figure S38: Rectangular Alistat heatmap showing pairwise alignment completeness scores for all species included in supermatrix D. Values closer to 1 indicate higher completeness scores for the pairwise sequence comparisons.

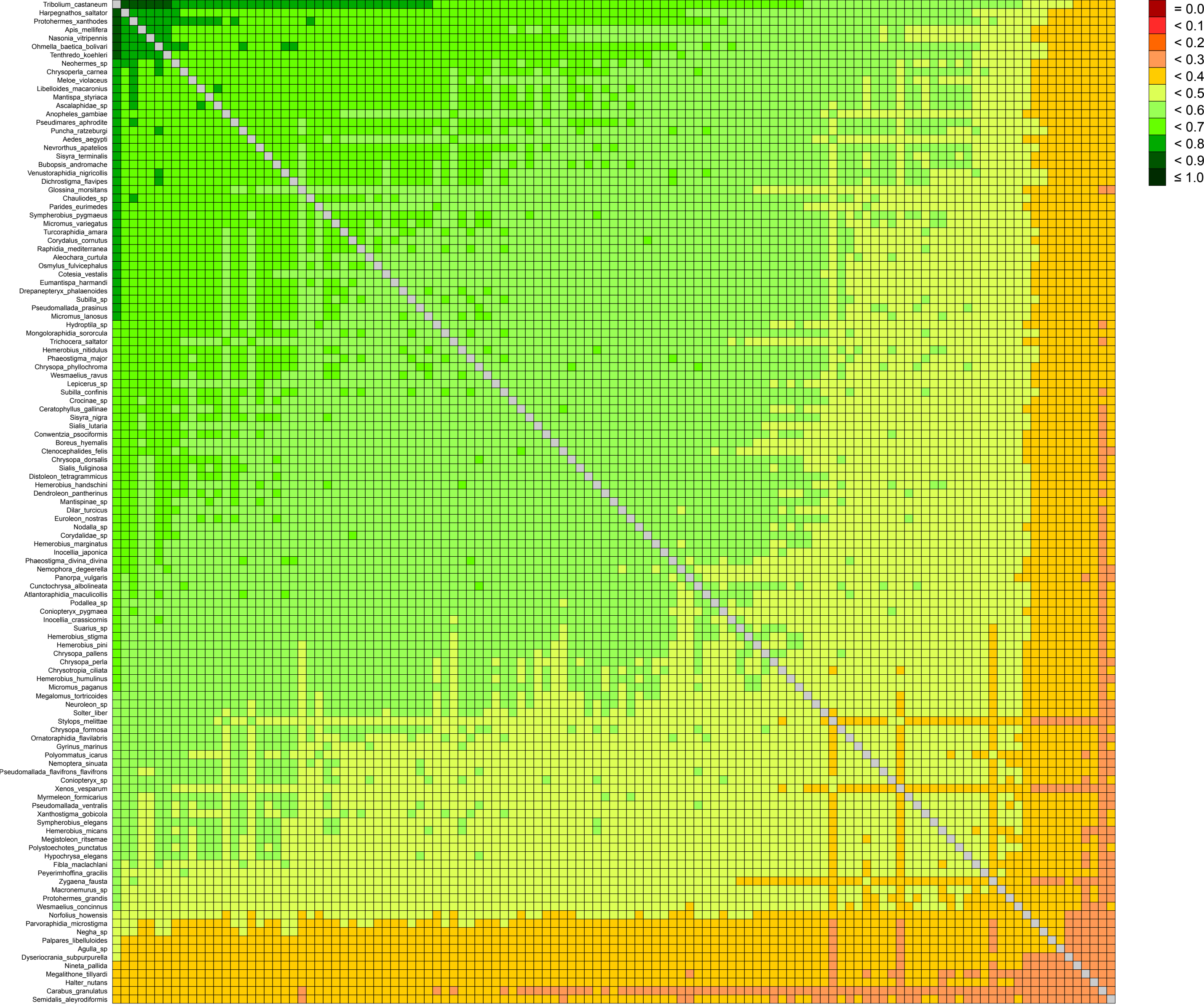

Figure S39: Rectangular Alistat heatmap showing pairwise alignment completeness scores for all species included in supermatrix E. Values closer to 1 indicate higher completeness scores for the pairwise sequence comparisons.

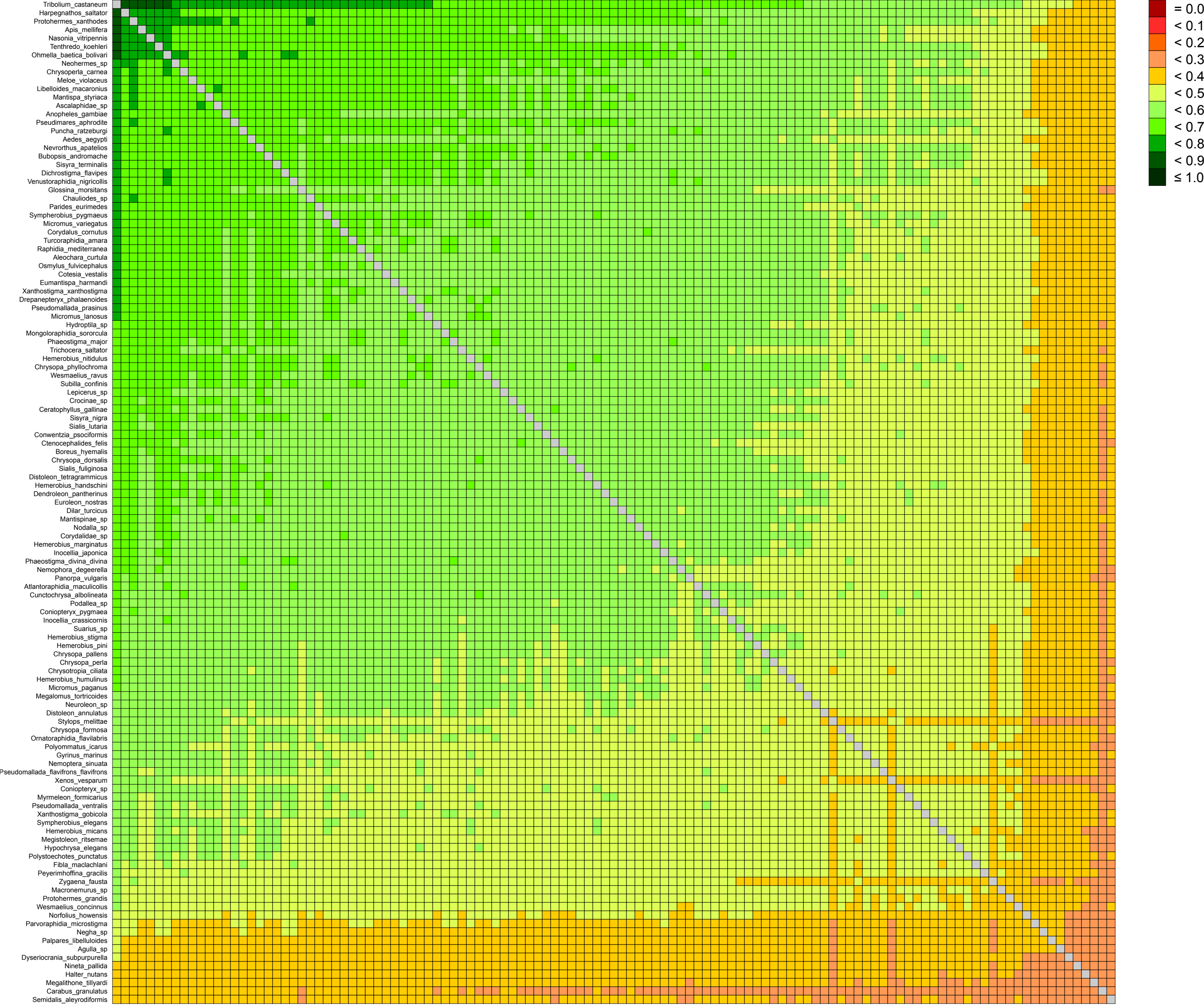

Figure S40: Rectangular Alistat heatmap showing pairwise alignment completeness scores for all species included in supermatrix F. Values closer to 1 indicate higher completeness scores for the pairwise sequence comparisons.

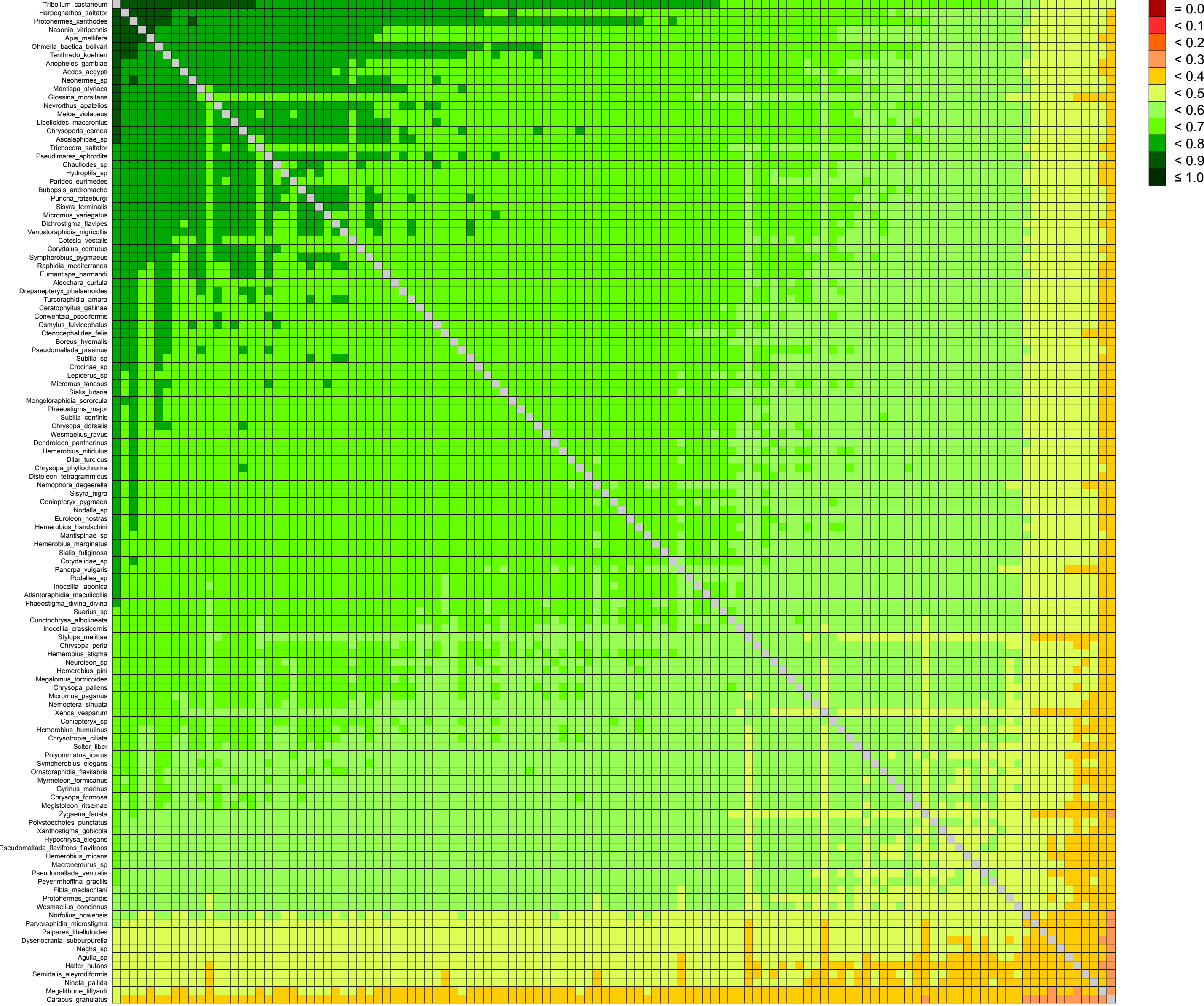

Figure S41: Rectangular Alistat heatmap showing pairwise alignment completeness scores for all species included in the RCFV-corrected version of supermatrix E. Values closer to 1 indicate higher completeness scores for the pairwise sequence comparisons.

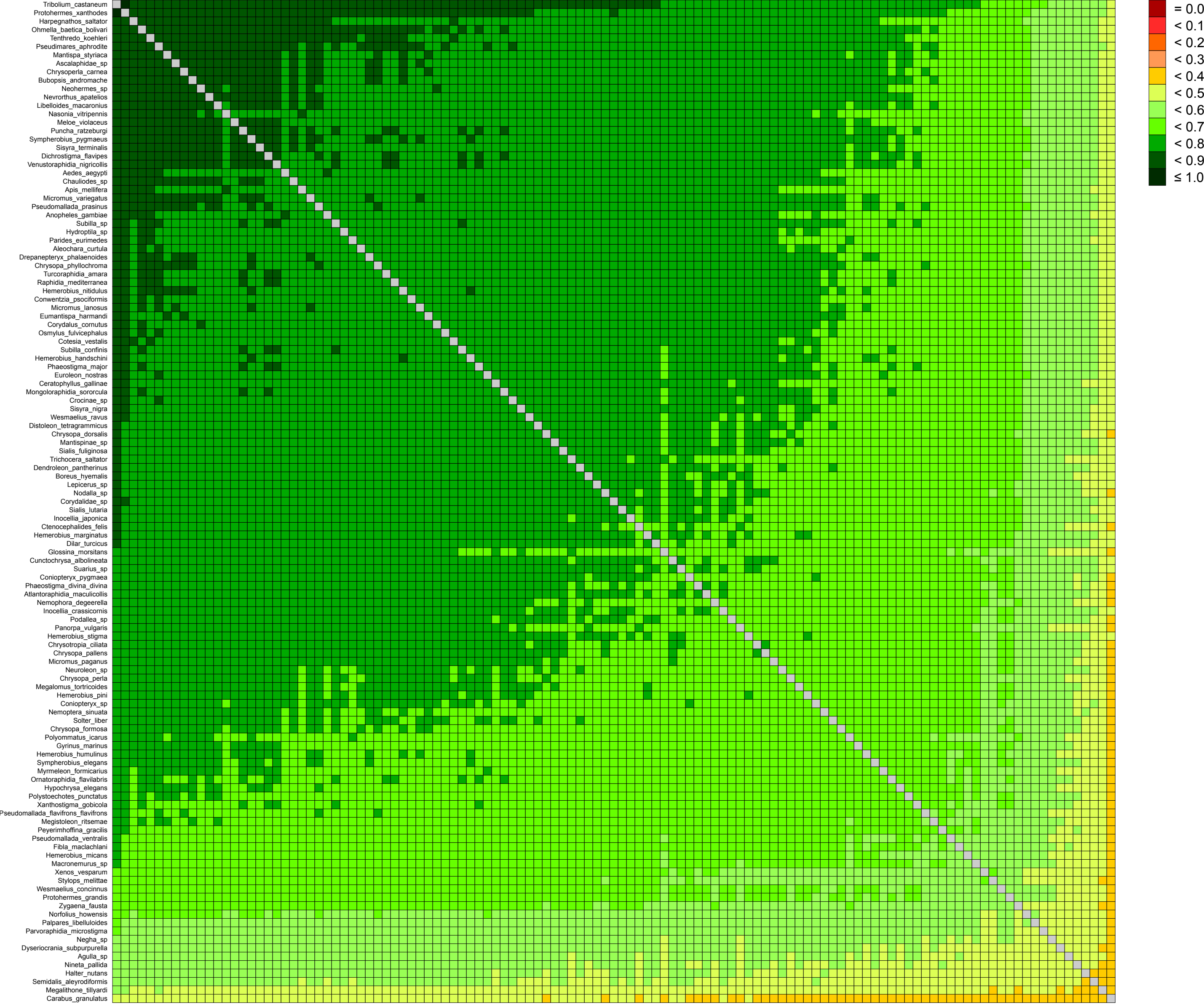

Figure S42: Alistat heatmap showing pairwise alignment completeness scores for all species included in the decisive version of supermatrix E. Values closer to 1 indicate higher completeness scores for the pairwise sequence comparisons.

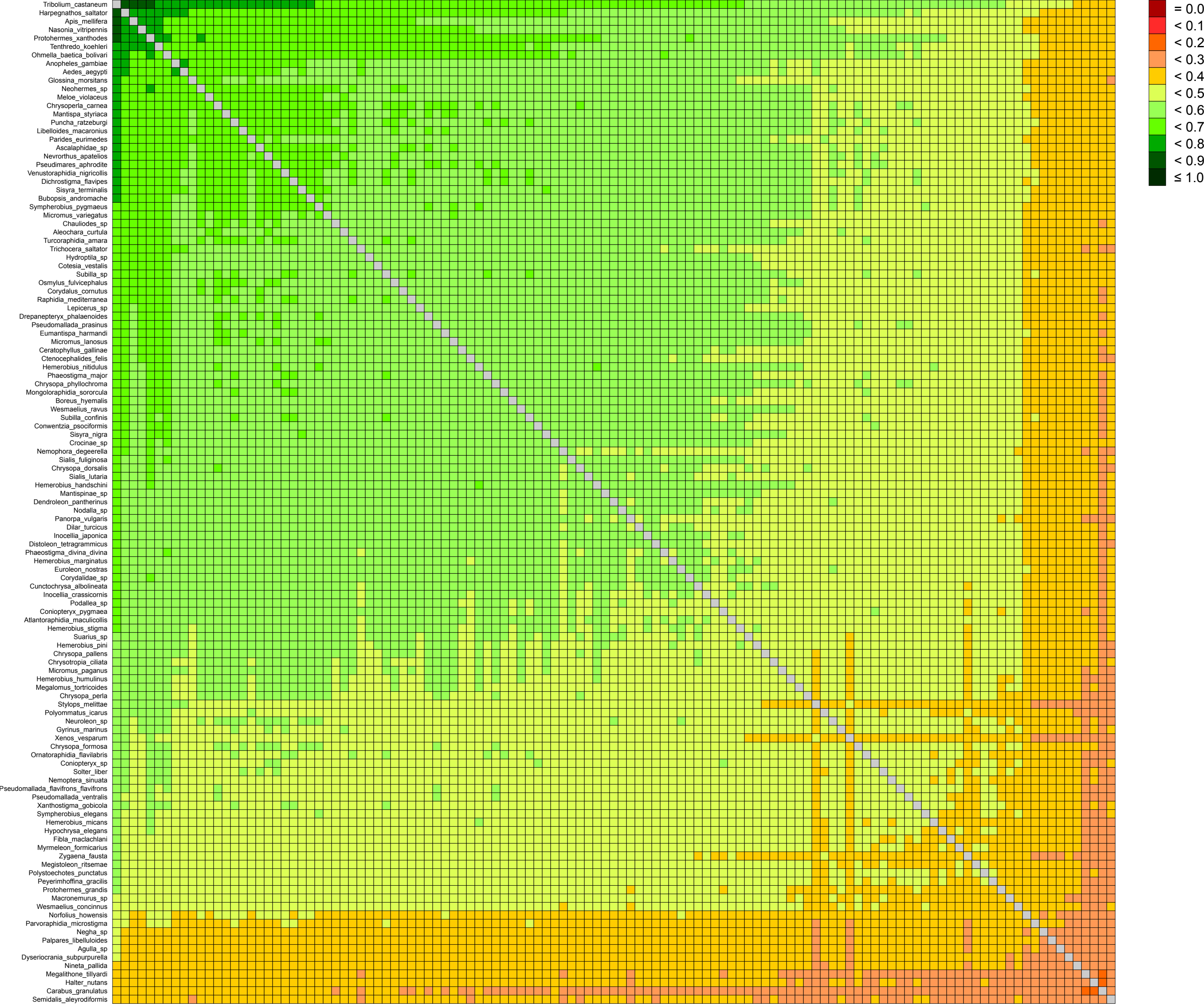

Figure S43: Rectangular Alistat heatmap showing pairwise alignment completeness scores for all species included in supermatrix D-nt. Values closer to 1 indicate higher completeness scores for the pairwise sequence comparisons.

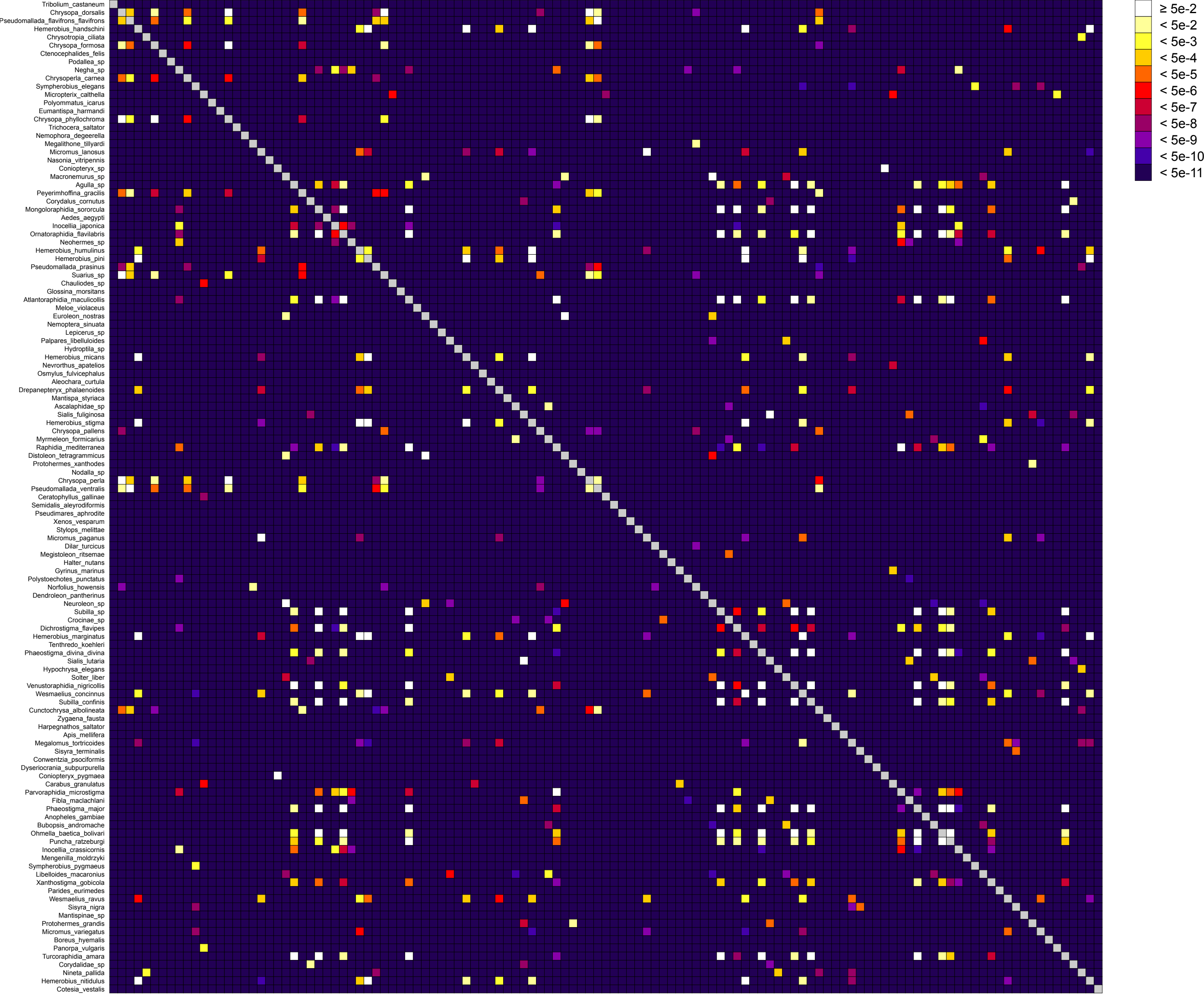

Figure S44: Full rectangular heat map calculated with SymTest showing p-values for the pairwise Bowker's tests in supermatrix A. Darker boxes indicate lower p-values and thus larger deviation from evolution under SRH conditions.

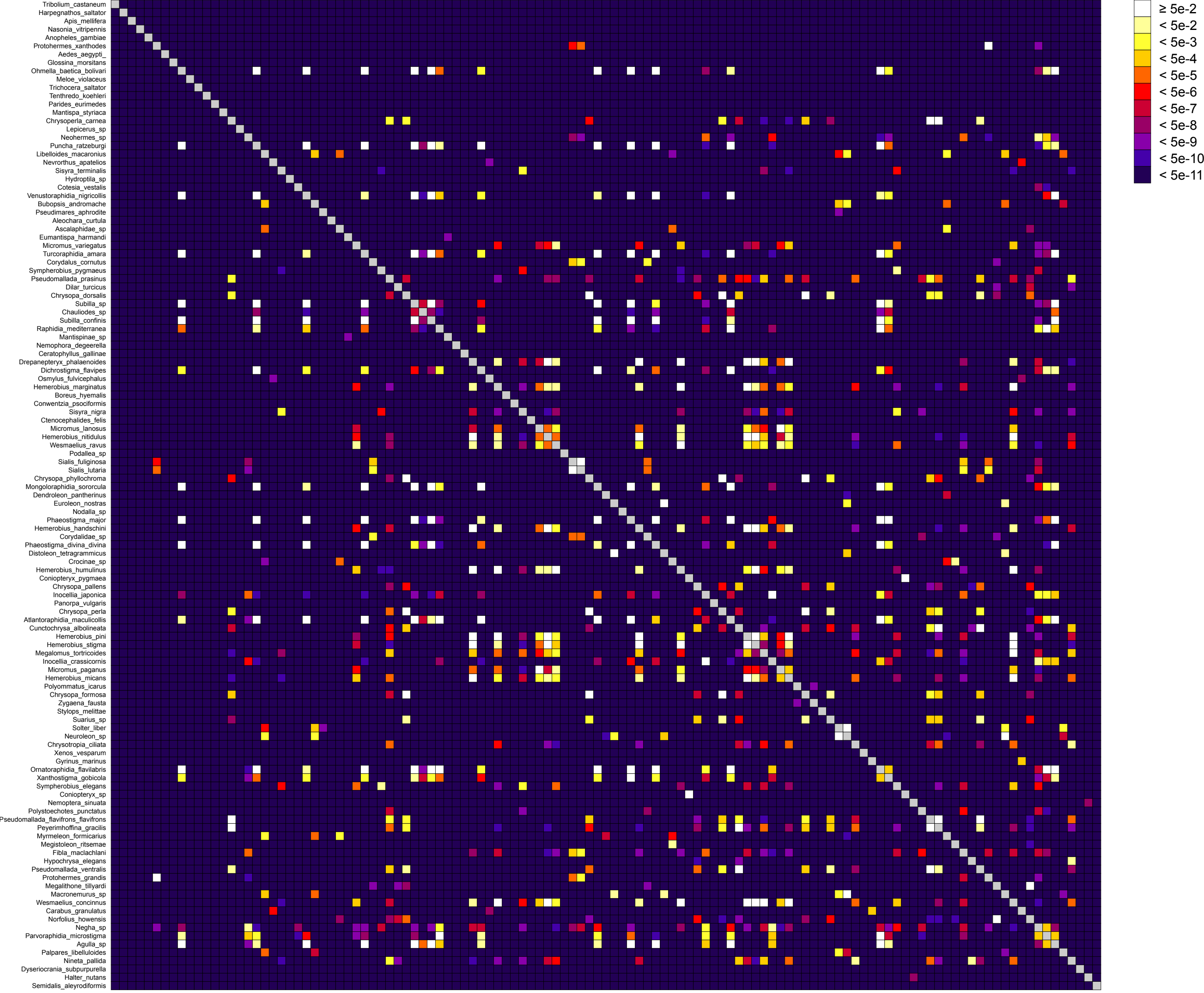

Figure S45: Full rectangular heat map calculated with SymTest showing p-values for the pairwise Bowker's tests in supermatrix B. Darker boxes indicate lower p-values and thus larger deviation from evolution under SRH conditions.

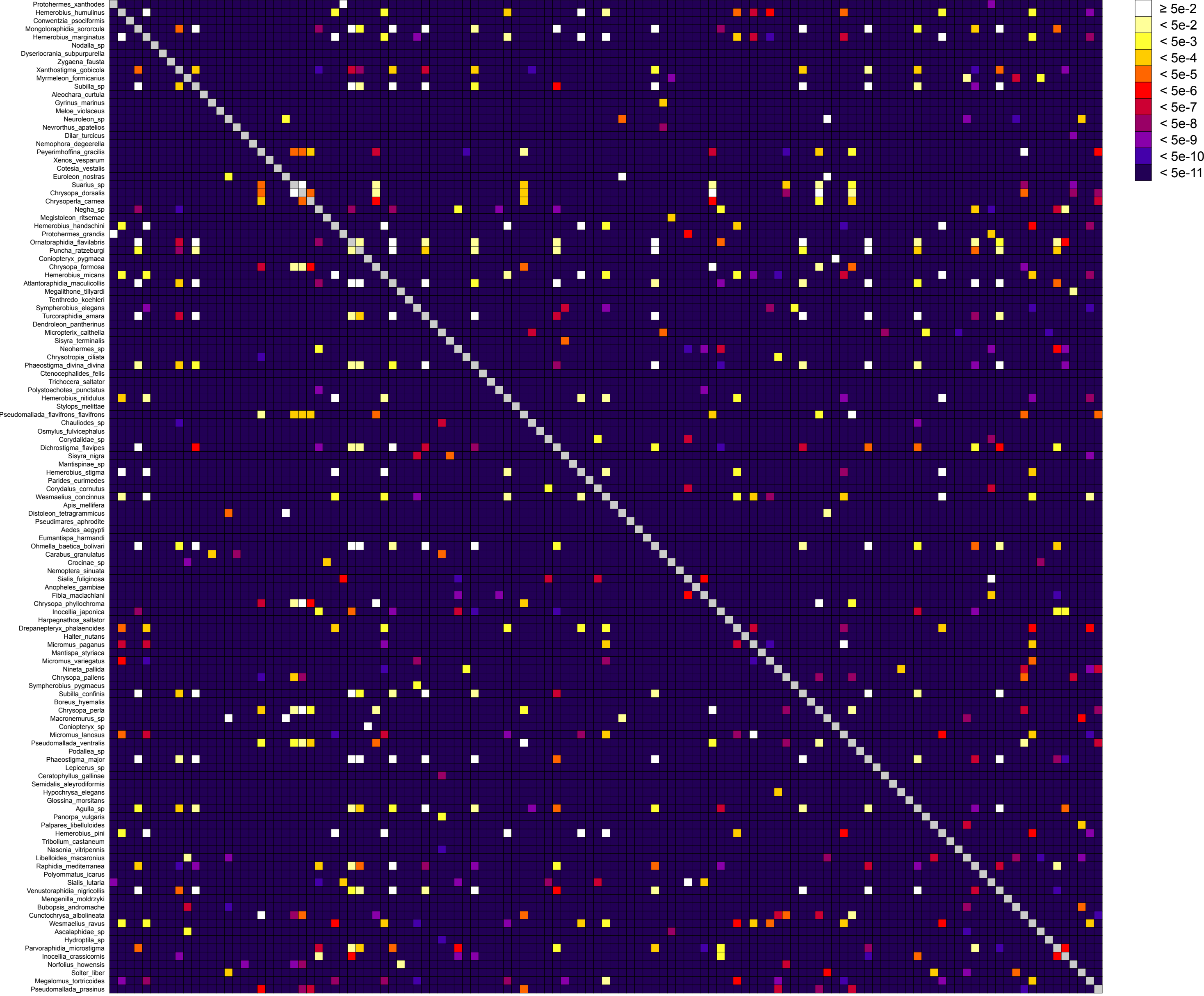

Figure S46: Full rectangular heat map calculated with SymTest showing p-values for the pairwise Bowker's tests in supermatrix C. Darker boxes indicate lower p-values and thus larger deviation from evolution under SRH conditions.

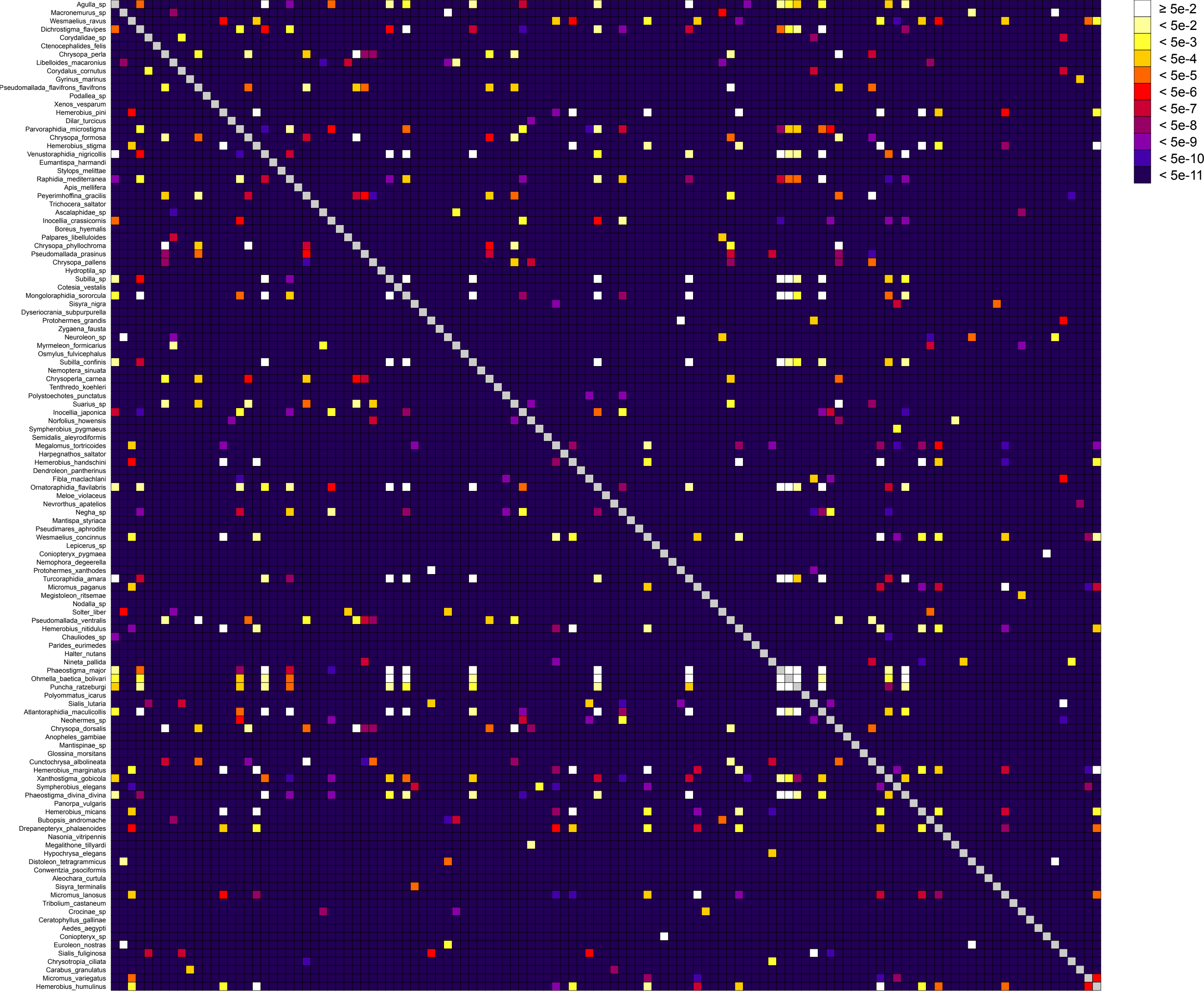

Figure S47: Full rectangular heat map calculated with SymTest showing p-values for the pairwise Bowker's tests in supermatrix D. Darker boxes indicate lower p-values and thus larger deviation from evolution under SRH conditions.

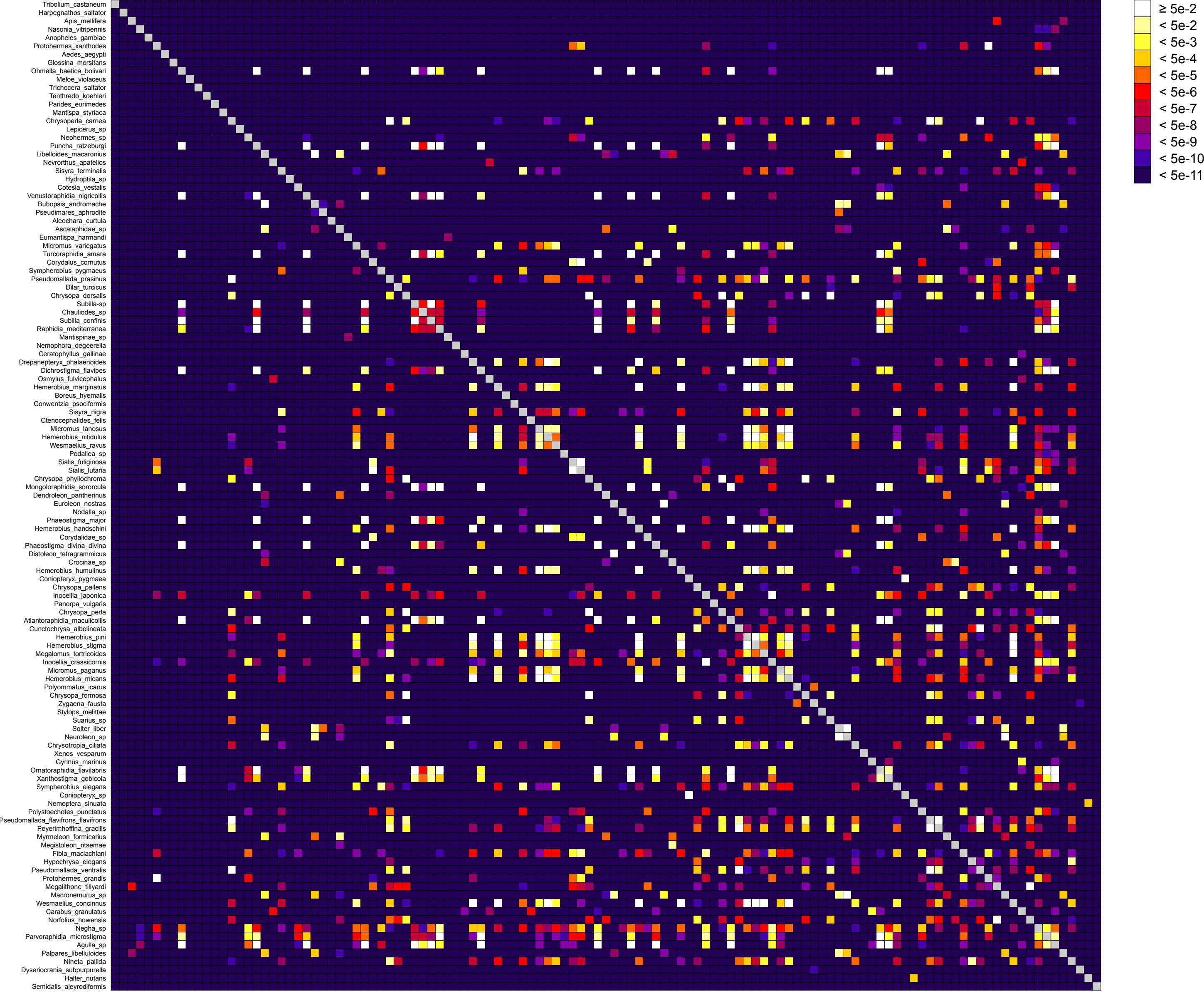

Figure S48: Full rectangular heat map calculated with SymTest showing p-values for the pairwise Bowker's tests in supermatrix E. Darker boxes indicate lower p-values and thus larger deviation from evolution under SRH conditions.

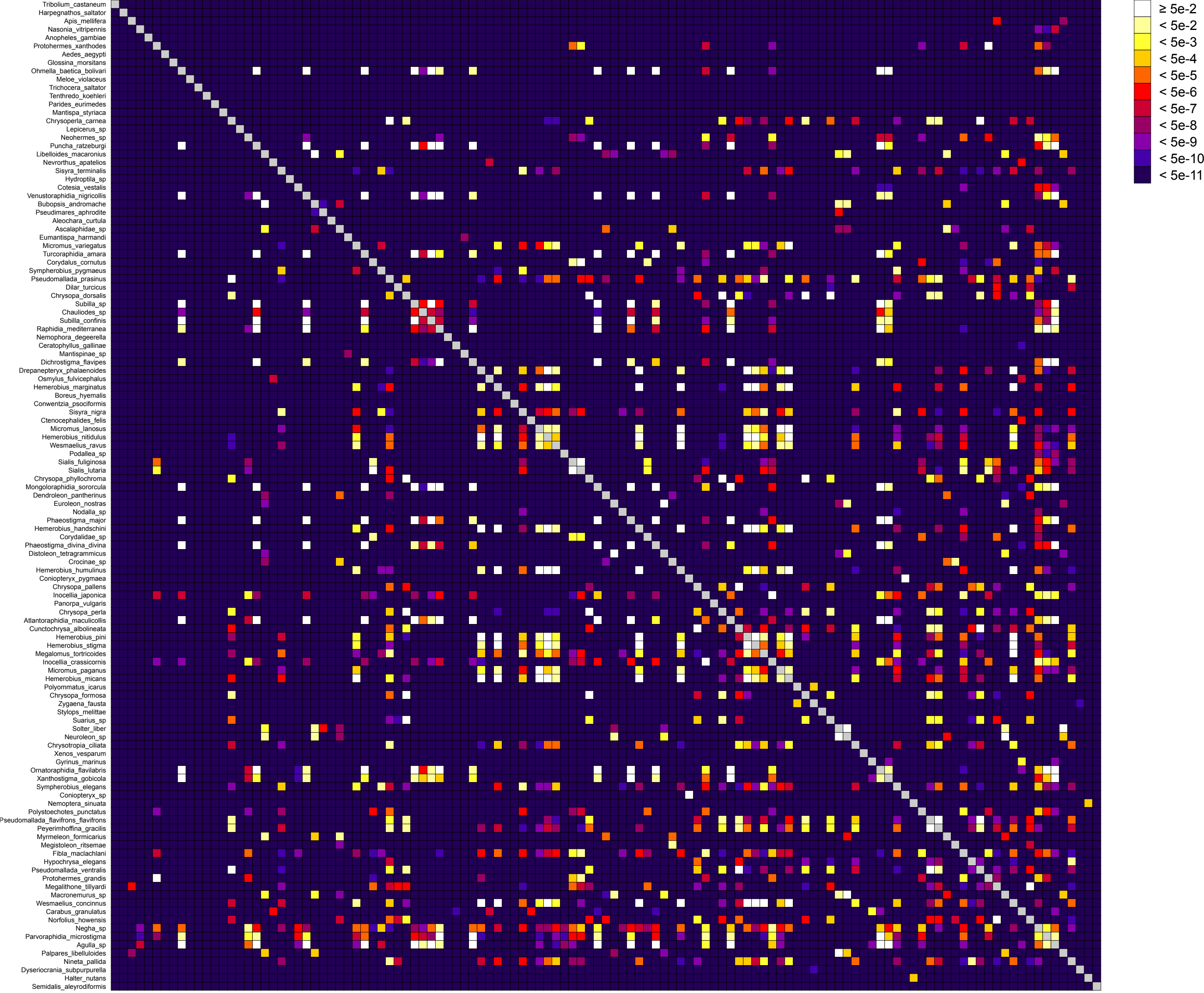

Figure S49: Full rectangular heat map calculated with SymTest showing p-values for the pairwise Bowker's tests in supermatrix F. Darker boxes indicate lower p-values and thus larger deviation from evolution under SRH conditions.

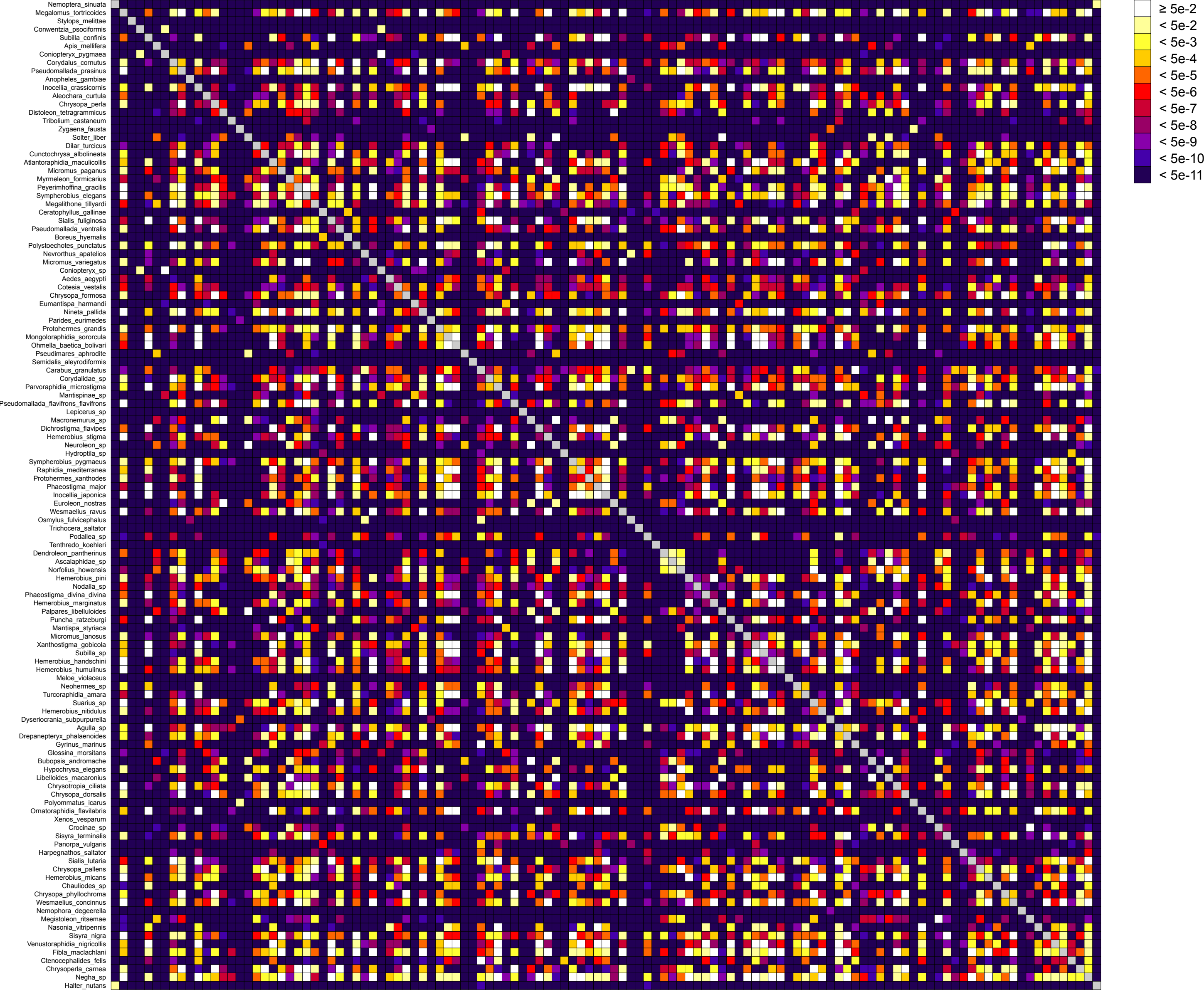

Figure S50: Full rectangular heat map calculated with SymTest showing p-values for the pairwise Bowker's tests in in the RCFV-corrected version of supermatrix E. Darker boxes indicate lower p-values and thus larger deviation from evolution under SRH conditions.

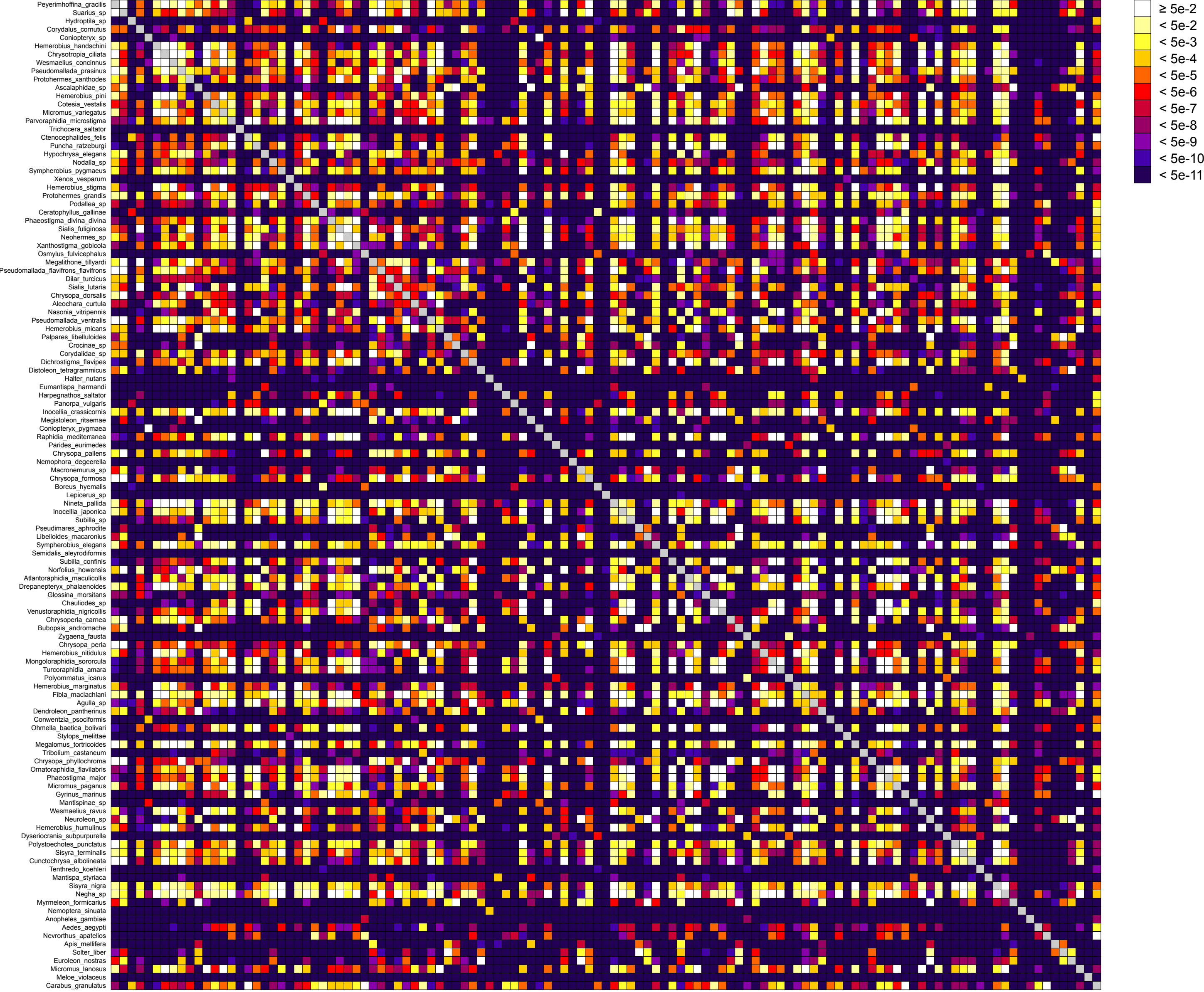

Figure S51: Full rectangular heat map calculated with SymTest showing p-values for the pairwise Bowker's tests in in the decisive version of supermatrix E (supermatrix E-decisive). Darker boxes indicate lower p-values and thus larger deviation from evolution under SRH conditions.

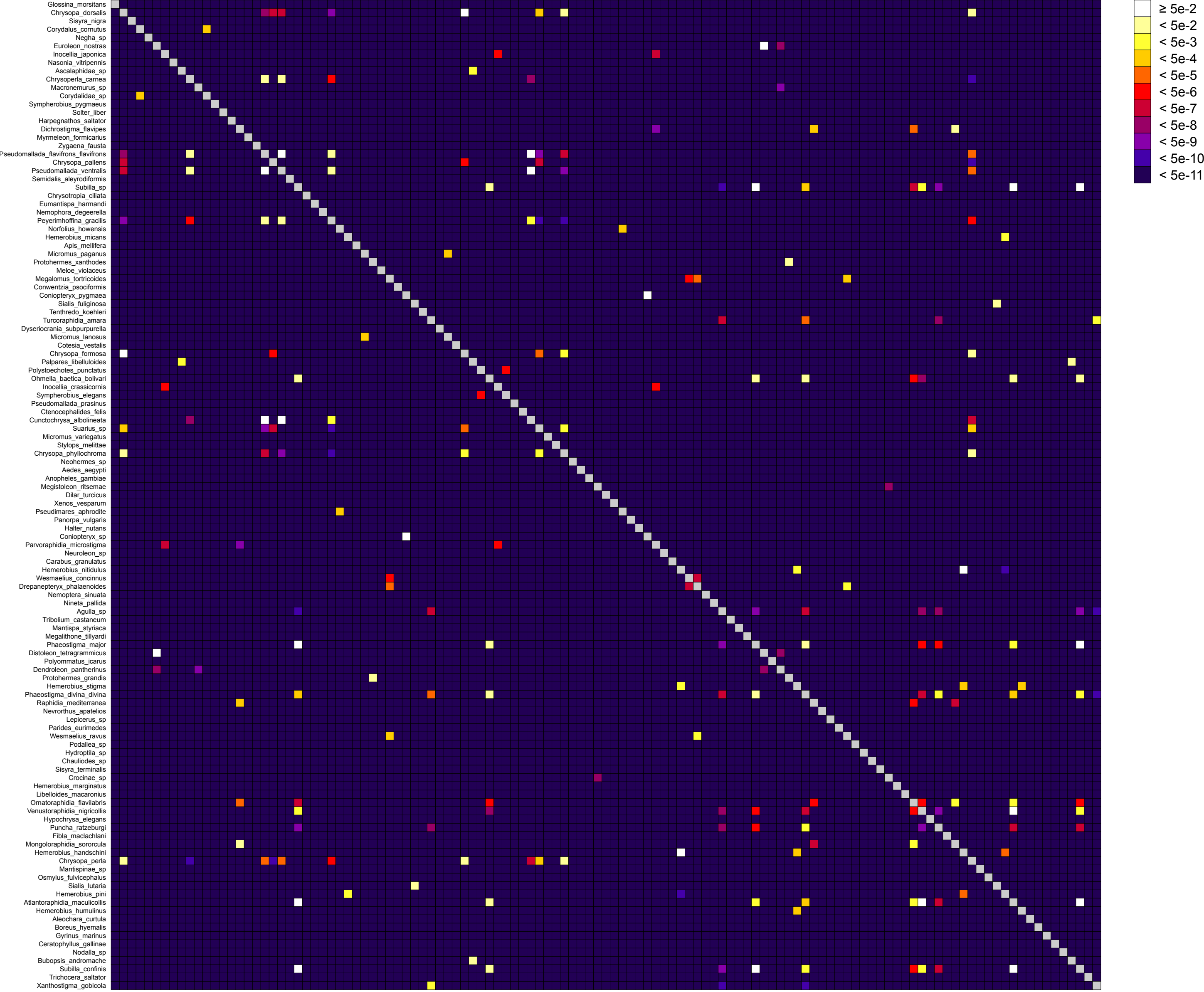

Figure S52: Full rectangular heat map calculated with SymTest showing p-values for the pairwise Bowker's tests when considering only the first codon positions in the corresponding nucleotide matrix of supermatrix D. Darker boxes indicate lower p-values and thus larger deviation from evolution under SRH conditions.

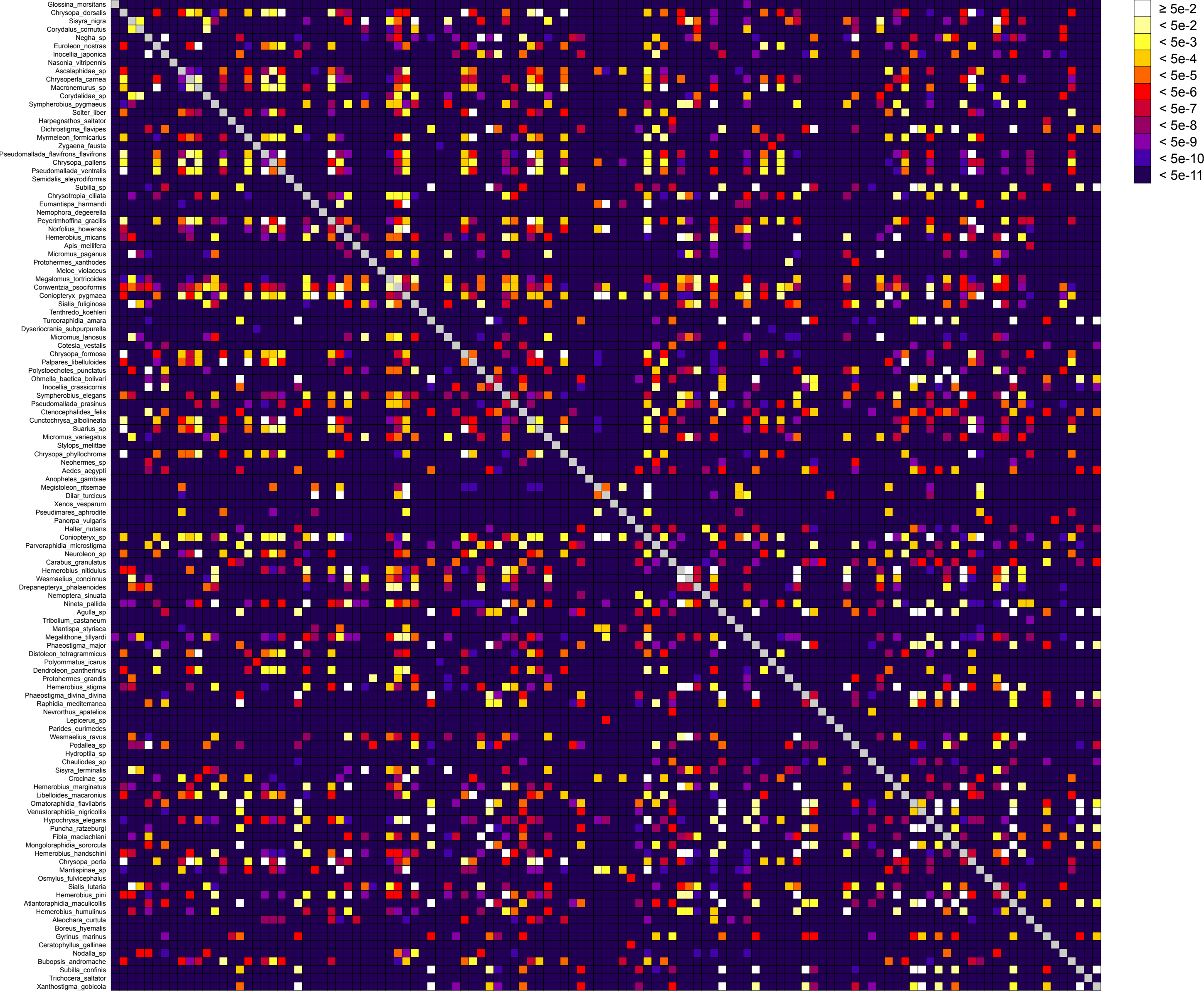

Figure S53: Full rectangular heat map calculated with SymTest showing p-values for the pairwise Bowker's tests when considering only the second codon positions in the corresponding nucleotide matrix of supermatrix D. Darker boxes indicate lower p-values and thus larger deviation from evolution under SRH conditions.

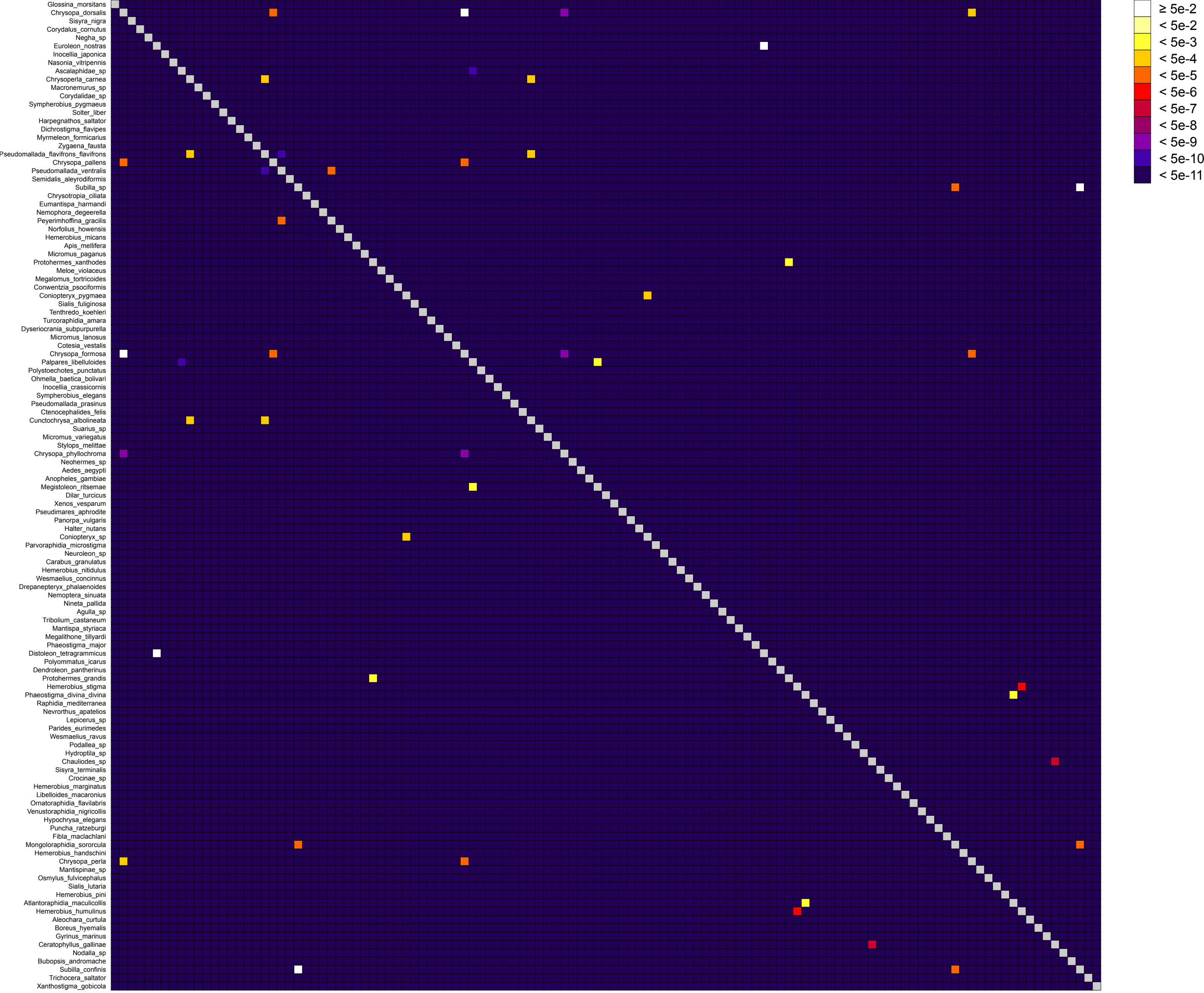

Figure S54: Full rectangular heat map calculated with SymTest showing p-values for the pairwise Bowker's tests when considering only the third codon positions in corresponding nucleotide matrix of supermatrix D. Darker boxes indicate lower p-values and thus larger deviation from evolution under SRH conditions.

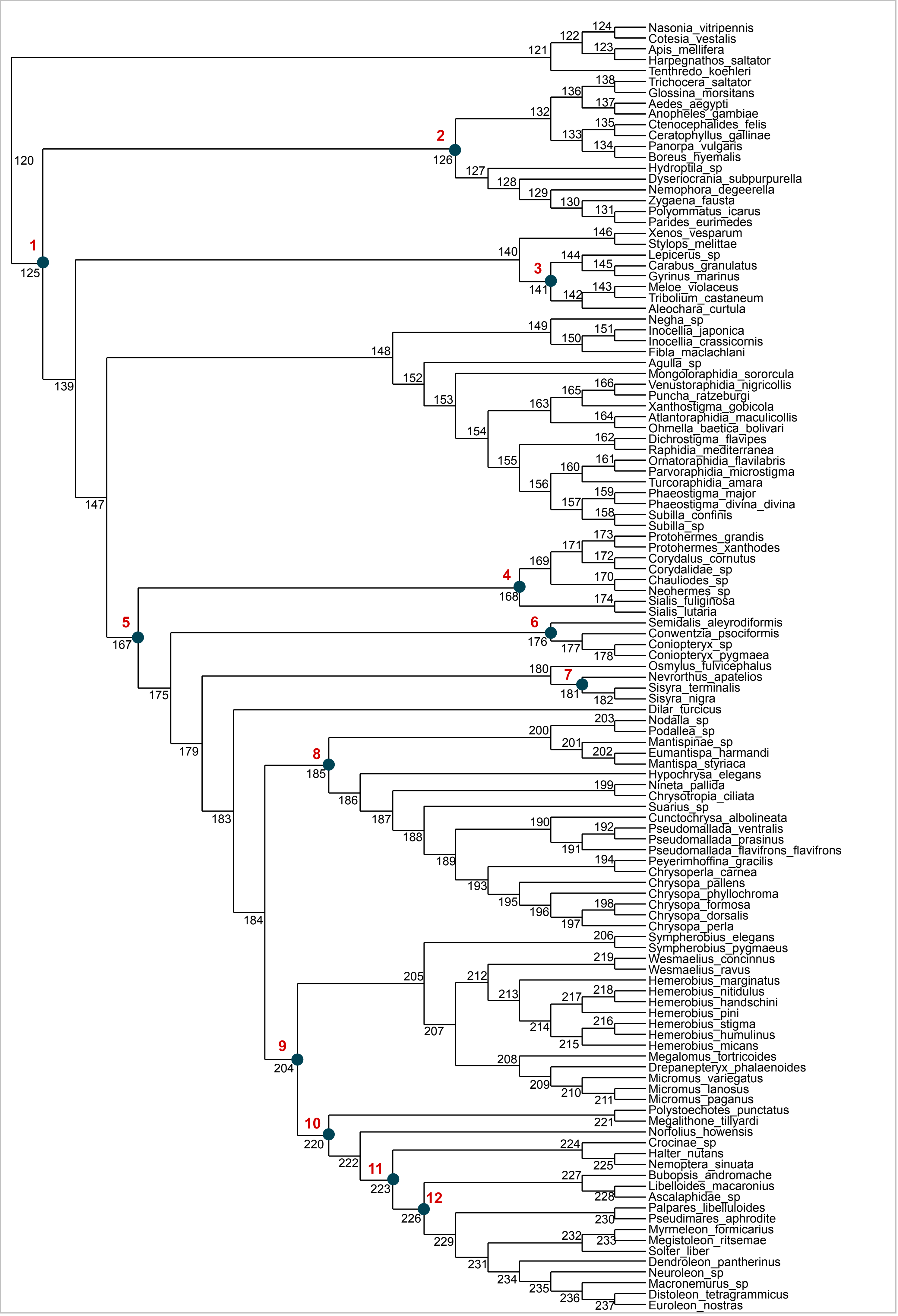

Figure S55: Cladogram showing the phylogenetic relationships that resulted from the analysis of supermatrix E. Values on nodes correspond to the node number IDs in Tables S11–S15.
Time calibrated nodes are shown in red. The numbers of the calibrated nodes correspond to the numbers of the fossils in Table S8 and in Figure 1 of the main text.

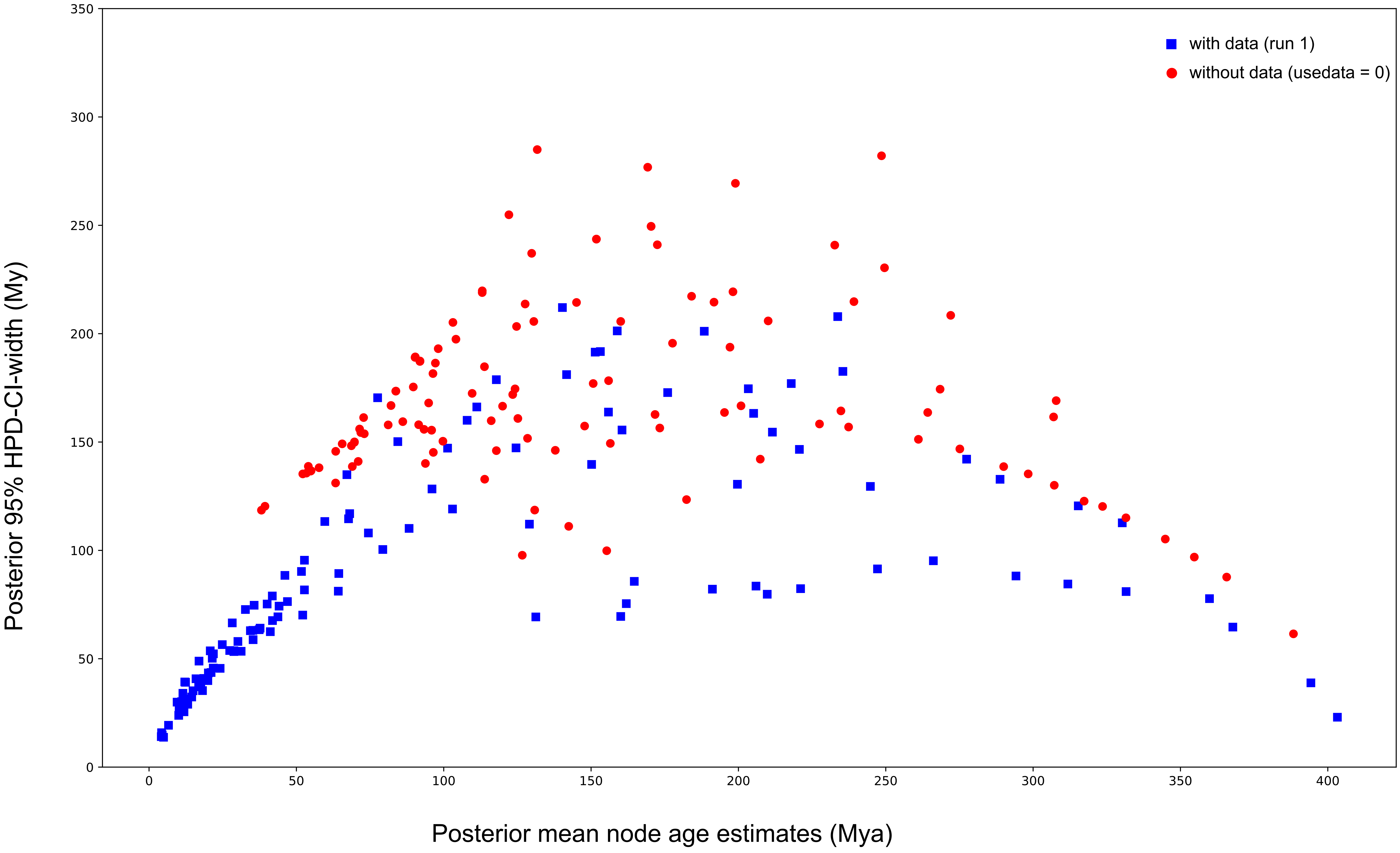

Figure S56: Scatter plot of the mean posterior node age estimates plotted against the 95 % higher posterior density CI-width of each node. The results from the first run with the approximate likelihood method and by including all fossil calibrations are plotted in blue. The results from the run without using the data (marginal prior) are plotted in red.
